# CENP-N promotes the compaction of centromeric chromatin

**DOI:** 10.1038/s41594-022-00758-y

**Published:** 2022-04-14

**Authors:** Keda Zhou, Magdalena Gebala, Dustin Woods, Kousik Sundararajan, Garrett Edwards, Dan Krzizike, Jeff Wereszczynski, Aaron F. Straight, Karolin Luger

**Affiliations:** 1 Department of Biochemistry, University of Colorado at Boulder, Boulder, CO USA; 2 Department of Biochemistry, Stanford University, Stanford, CA USA; 3 Department of Chemistry and the Center for Molecular Study of Condensed Soft Matter, Illinois Institute of Technology, Chicago, IL USA; 4 Department of Physics and the Center for Molecular Study of Condensed Soft Matter, Illinois Institute of Technology, Chicago, IL USA; 5 Howard Hughes Medical Institute, University of Colorado at Boulder, Boulder, CO USA

**Keywords:** Cryoelectron microscopy, Centromeres

## Abstract

The histone variant CENP-A is the epigenetic determinant for the centromere, where it is interspersed with canonical H3 to form a specialized chromatin structure that nucleates the kinetochore. How nucleosomes at the centromere arrange into higher order structures is unknown. Here we demonstrate that the human CENP-A-interacting protein CENP-N promotes the stacking of CENP-A-containing mononucleosomes and nucleosomal arrays through a previously undefined interaction between the α6 helix of CENP-N with the DNA of a neighboring nucleosome. We describe the cryo-EM structures and biophysical characterization of such CENP-N-mediated nucleosome stacks and nucleosomal arrays and demonstrate that this interaction is responsible for the formation of densely packed chromatin at the centromere in the cell. Our results provide first evidence that CENP-A, together with CENP-N, promotes specific chromatin higher order structure at the centromere.

## Abstract (web summary)

Cryo-EM and biochemical analyses reveal that centromere-associated protein CENP-N promotes centromere-specific nucleosome stacking and higher order structures in vitro and in the cell.

## Main

The three-dimensional arrangement of nucleosomes (each consisting of 2 copies of histones H3, H4, H2A, and H2B that wrap 147 base pairs (bp) of DNA^1^) determines local and global chromatin architecture in all eukaryotes. Although many in vitro studies provide evidence for a defined 30-nm fiber where nucleosomes are regularly packed through the interactions between n and n + 2 nucleosomes, this has not been observed in the cell^2–4^. Instead, chromatin fibers are folded irregularly and diversely, with much variability between cell states and genome loci. Molecular-dynamics (MD) simulations suggest that the energy barriers between different nucleosome arrangements are relatively low^5^. Variations of nucleosome composition, such as DNA sequence, length of linker DNA connecting individual nucleosomes, incorporation of histone variants, and post-translational modifications of histones all have the potential to affect chromatin condensation and thus DNA accessibility, either directly or through the recruitment of a plethora of interacting factors^6^.

The centromere is a specialized chromatin region onto which the kinetochore assembles. This megadalton complex of >100 proteins ultimately promotes faithful chromosome segregation by forming attachment points to the mitotic spindle. Nucleosomes containing the centromeric histone H3 variant CENP-A are interspersed among canonical nucleosomes (possibly in a clustered arrangement^7^), providing the sole epigenetic determinant of the centromere^8^. The crystal structure of CENP-A-containing nucleosomes shows that it stably binds only 121 bp of DNA, rather than wrapping the canonical 147 bp of DNA^9^. This results in the unique arrangement of CENP-A containing tri-nucleosomal arrays^10^, and renders the CENP-A nucleosome unable to bind linker histone H1 (refs. ^11–13^). The key function of CENP-A nucleosomes appears to be the recruitment of centromere-specific proteins, most notably CENP-N and CENP-C, both of which recognize unique features of nucleosomal CENP-A, and upon which the CCAN (constitutive centromere-associated network) complex, and ultimately the kinetochore, assemble (reviewed in ref. ^14^). Both CENP-N and CENP-C affect CENP-A nucleosome dynamics and structure in vitro at the mononucleosome level^15–20^, but their effect on chromatin higher order structure has not been investigated^21^.

## Results

### CENP-N promotes the stacking of CENP-A mononucleosomes

**Extended Data Fig. 1:**
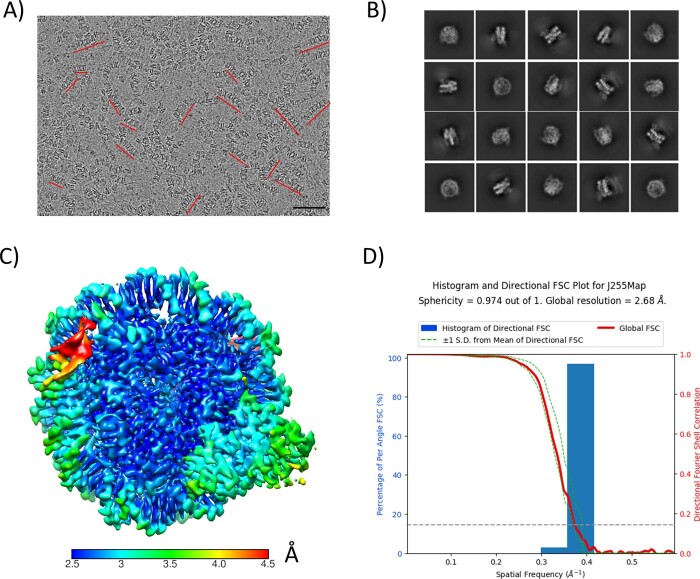
CryoEM analysis of CENP-N in complex with CENP-A mono-nucleosomes (reconstituted with 147 bp palindromic α satellite DNA). **a**) Raw cryoEM micrograph. Stacks are indicated by red lines. Size bar is 50 nm. **b)** 2D classifications of particles at the mono-nucleosome level. **c)** Local resolution of the 3D map for CENP-N in complex with CENP-A mono-nucleosomes. The color key indicates the resolution. **d)** 3DFSC analysis of reconstructed 3D map.

As one of two pillars for kinetochore assembly at the centromere, CENP-N recognizes the CENP-A nucleosome through its amino-terminal region, while forming a heterodimer with CENP-L through its carboxy-terminal region to recruit other CCAN proteins. Previously, we reported the cryogenic electron microscopy (cryo-EM) structure of a CENP-A nucleosome reconstituted with the 601 nucleosome-positioning DNA sequence in complex with the nucleosome-binding domain of CENP-N (CENP-N^1–289^)^16^. In this structure, CENP-N makes extensive contacts with DNA through the pyrin domain and the CNL-HD (CENP-N-CENP-L homology domain) and specifically recognizes the CENP-A histone through the α1 helix and β3–β4 loop of CENP-N^16^. However, α-satellite DNA is more typical of the DNA sequence at the centromere, and we therefore determined the cryo-EM structure of the CENP-A nucleosome, reconstituted onto palindromic α-satellite DNA^1^ and bound to CENP-N^1–289^, to a resolution of 2.7 Å (Extended Data Fig. 1). This DNA fragment was chosen because of its first use in the structure determination of the nucleosome^1^, where it was shown to exhibit precise positioning around the histone octamer. This structure is very similar to previously published structures of the CENP-N nucleosome, CENP-A complexes reconstituted with the 601 DNA sequence^16,17,22^, and a CENP-A nucleosome structure reconstituted onto native α-satellite DNA^23^ (see additional analysis and discussion in the Supplementary Information) (Extended Data Fig. 10). This demonstrates that DNA sequence has little effect on the overall configuration of CENP-A nucleosomes, although the dynamic behavior of the penultimate ~10 bp differs between different DNA sequences.

**Fig. 1:**
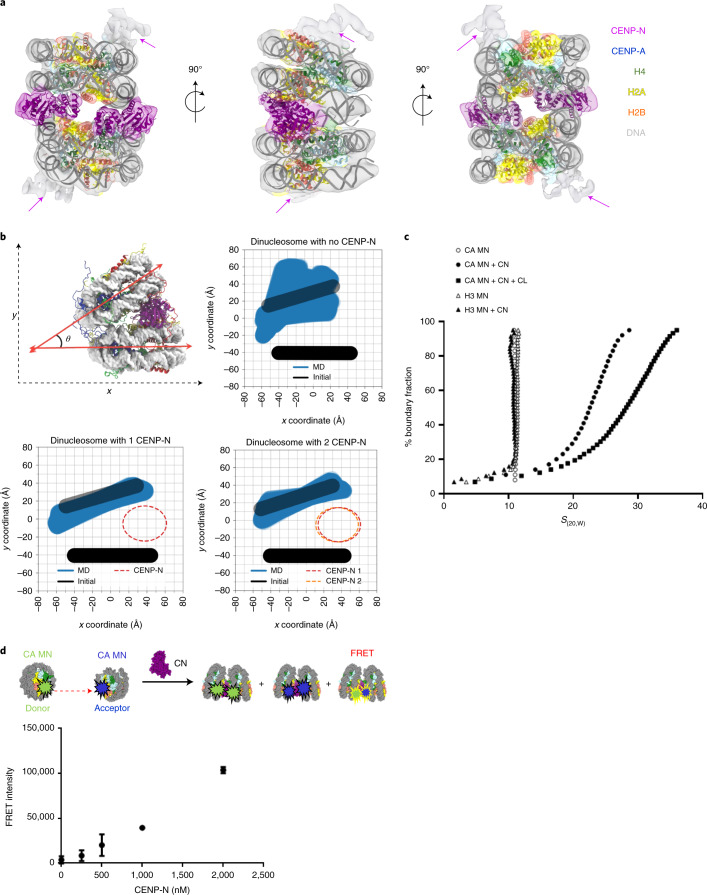
CENP-N mediates CENP-A nucleosome stacking *in vitro*. **a**, A model of two CENP-A nucleosomes (α satellite DNA) connected by two copies of CENP-N was fit into the density map. Protein identity is indicated by color codes. Arrows highlight the weak density attributed to a second CENP-N on the other side of the CENP-A nucleosomes. **b**, Simulation plots of nucleosome stacks in the absence or presence of CENP-N. This plot used two coordinate points from each nucleosome, namely the geometric center of the C1′ atoms from nucleotides located at the dyad and opposite of the dyad (Extended Data Fig. 4 for illustration). The C1’ atoms of every nucleotide in one nucleosome, pictured as the bottom nucleosome of each graph in Fig. 1b, were used to align each frame of the trajectory. The dyad and opposing points in the other nucleosome, pictured as the top nucleosome of each graph in Fig. 1b, were plotted to depict the relative sampling of each stacked-nucleosome system. **c**, SV-AUC (enhanced van Holde–Weischet plots) for CENP-A (CA) or H3 mononucleosomes (MN) in complex with CENP-N^1–289^ (CN) or full-length CENP-N/CENP-L (CN + CL). **d**, FRET analysis of CENP-A mononucleosome (CA MN) interactions in the absence or presence of CENP-N. The donor is CENP-A mononucleosomes containing Alexa 488-labeled H2B; the acceptor is a CENP-A mononucleosome containing Atto N 647-labeled H2B (250 nM donor and acceptor nucleosome concentrations were used). FRET intensity changes in dependence of [CENP-N]. The final NaCl concentration was 70 mM. Error bars are from four independent measurements of two biological replicates. Data are presented as mean values ± s.d. Source data

**Extended Data Fig. 2:**
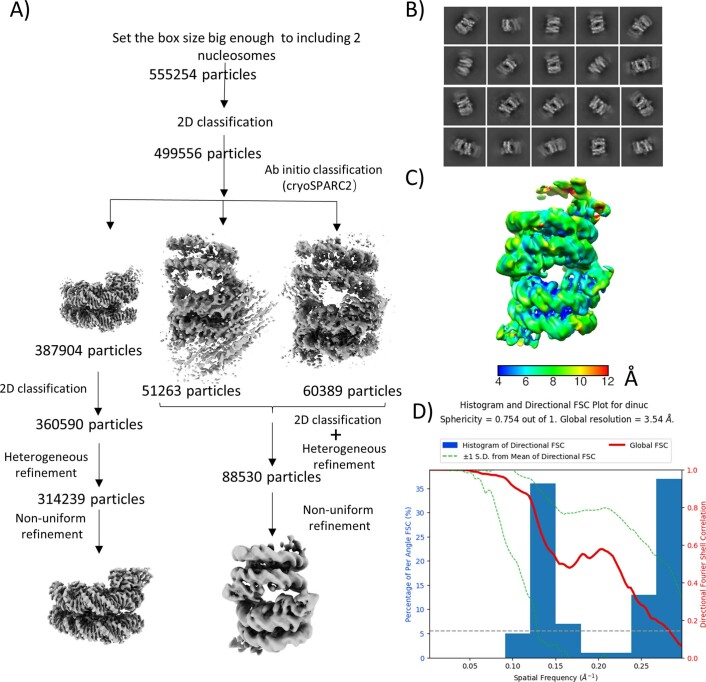
CryoEM analysis of CENP-N mediated stacks of CENP-A mono-nucleosomes reconstituted with palindromic α-satellite DNA. **a**) Flow-chart of the analysis of di-nucleosomes in the stacks. **b)** 2D classification of di-nucleosomes with CENP-N. **c)** Local resolution of the 3D map for stacked CENP-A nucleosomes in complex with CENP-N. **d)** 3DFSC analysis of the reconstructed 3D map. The plot reveals significant orientation bias in the map reconstruction. Therefore, the estimated global resolution of 3.54 Å does not represent the overall map quality. In some orientations, the resolution is closer to 10 Å.

**Extended Data Fig. 3:**
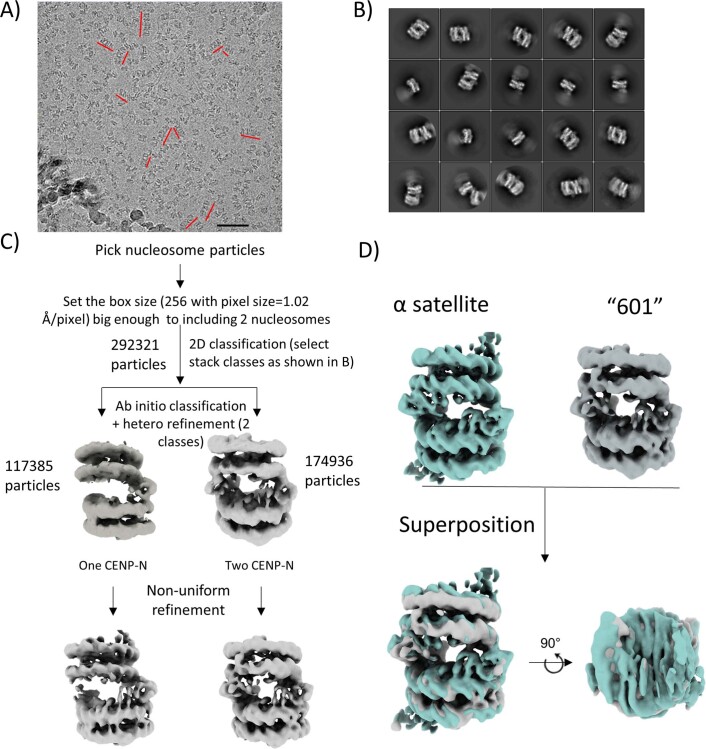
CryoEM analysis of CENP-N mediated stacks of CENP-A mono-nucleosomes reconstituted with 601 DNA. **a)** Representative cryoEM micrograph. Stacks are highlighted by red lines. Size bar is 50 nm**. b)** 2D classification of the di-nucleosome with CENP-N in the stacks. **c)** Flow-chart on the analysis of di-nucleosomes in the stacks. **d)** Comparison of di-nucleosome maps derived for α satellite and 601 CENP-A nucleosomes.

In the cryo-EM images, we consistently observed that ~30% of CENP-A nucleosomes form ordered stacks on the grid in the presence of CENP-N, ranging from 2–10 nucleosomes (Extended Data Figs. 1a and 2). CENP-A nucleosomes reconstituted with the 601 nucleosome-positioning sequence^24^ exhibit the same behavior in the presence of CENP-N (Extended Data Fig. 3a). By focusing single-particle analysis on nucleosome pairs contained in these stacks, defined density for CENP-N was observed between two nucleosomes in the two-dimensional (2D) class averages for nucleosomes reconstituted on either DNA fragment (Extended Data Figs. 2b and 3b). After three-dimensional (3D) reconstruction and refinement (Extended Data Figs. 2a and 3c), we obtained cryo-EM maps in which one or two CENP-N^1–289^ could be unambiguously docked between two CENP-A nucleosomes (Table 1; Fig. 1a, shown for α-satellite nucleosomes). Superposition of 3D maps (Extended Data Fig. 3d) showed the exact same nucleosome stacks in cryo-EM datasets of CENP-N with CENP-A nucleosomes reconstituted onto the two DNA sequences, suggesting that the DNA-sequence context does not affect nucleosome stacking. As such, all of the following experiments were performed with 601 nucleosomes.

**Table 1:** Cryo-EM data collection, refinement and validation statistics

	AN mono (α sat) (EMDB-26330) (PDB 7U46)	AN stack (α sat) (EMDB-26331) (PDB 7U47)	AN stack (601) (EMDB-26332) (PDB 7U4D)	AN 12-mer (601) (EMDB-26333)
**Data collection and processing**				
Magnification	29,000		29,000	22,500
Voltage (kV)	300		300	300
Electron exposure (e^–^/Å^2^)	70		80	100
Defocus range (μm)	0.8–2.0		1.0–2.5	1.3–2.5
Pixel size (Å)	0.8211		1.02	0.655
Symmetry imposed	*C*_1_		*C*_1_	*C*_1_
Initial particle images (no.)	555,254		292,321	13,356
Final particle images (no.)	314,239	88,530	174,936	9,305
Map resolution (Å)	2.68	3.54	5.30	12.70
FSC threshold	0.143	0.143	0.143	0.143
Map resolution range (Å)	2.5–4.5	3.5–10	5.0–12.0	12.5–28.0
**Refinement**				
Initial model used (PDB code)	1KX5, 6C0W		6C0W	
Model resolution (Å)	2.7	3.8^a^	5.9^a^	
FSC threshold	0.143	0.143	0.143	
Model resolution range (Å)	2.5–4.5	3.5–9.4	5.9–11.9	
Map sharpening *B* factor (Å^2^)				
Model composition				
Non-hydrogen atoms	13,362	26,724	262,444	
Protein residues	925	1,850	1,850	
Nucleotide	290	580	556	
Ligands	0	0	0	
*B* factors (Å^2^)				
Protein	77.09	77.09	65.96	
Nucleotide	108.23	108.23	121.56	
Ligand	n/a	n/a	n/a	
R.m.s. deviations				
Bond lengths (Å)	0.011	0.011	0.006	
Bond angles (°)	0.781	0.913	0.972	
Validation				
MolProbity score	1.38	1.4	1.58	
Clashscore	4.10	4.41	4.65	
Poor rotamers (%)	0.00	0.00	0.00	
Ramachandran plot				
Favored (%)	96.91	96.91	95.08	
Allowed (%)	3.09	3.09	4.92	
Disallowed (%)	0.00	0.00	0.00	

^a^Due to severe orientation issues, the reported resolution is not an accurate reflection of the map quality.

**Extended Data Fig. 4:**
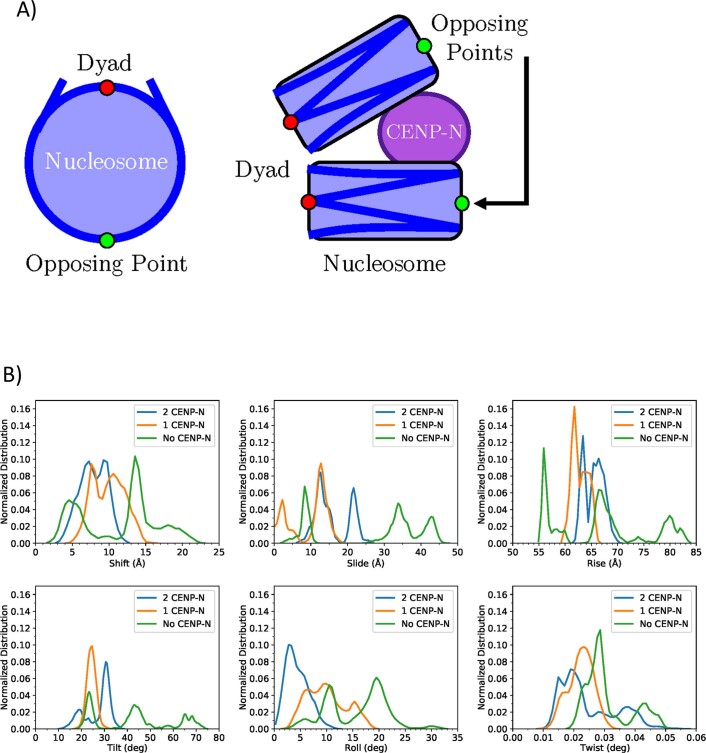
MD simulations of stacked nucleosomes with 0, 1, or 2 CENP-N. **a)** Diagram of the points used to construct stacked-nucleosome sampling graphs as depicted in Fig. 1c, in addition to nucleosome parameters calculated for stacked-nucleosome simulations. Nucleosomes (blue) are shown in face-on (left) and profile (right) viewpoints, with DNA represented in a darker blue. Dyad points and their opposing points are represented as small red and green circles, respectively. CENP-N is shown in purple to provide a point-of-reference. **b)** From left to right, six histograms of stacking parameters for di-nucleosome systems: Shift, Slide, Rise (top), and Tilt, Roll, Twist (bottom), in analogy to the parameters used to describe the geometry of the DNA double helix. Histograms do not contain the first 100 ns of simulation time which was allotted for each system to achieve equilibration.

MD simulations were performed to evaluate the stability of CENP-N binding to nucleosomes in silico and how it influences the dynamics of dinucleosomal stacking (Supplementary Movies 1–3). By plotting a cross-sectional view of the nucleosome coordinates, as seen in Fig. 1b, we were able to determine the relative stabilizing effect provided by each CENP-N to stacked nucleosomes. The stacking of two mononucleosomes is rather unstable in simulations without CENP-N, in which the nucleosomes explore a wide range of relative orientations. Stacked nucleosomes exhibit a similar amount of sampling whether they are in complex with one or two copies of CENP-N (Fig. 1b). Other metrics for the inter-nucleosomal interactions, including the relative rise, shift, and tilt, exhibited similar trends (Extended Data Fig. 4), suggesting that a singular CENP-N is sufficient to stably maintain stacking between two CENP-A nucleosomes.

**Extended Data Fig. 5:**
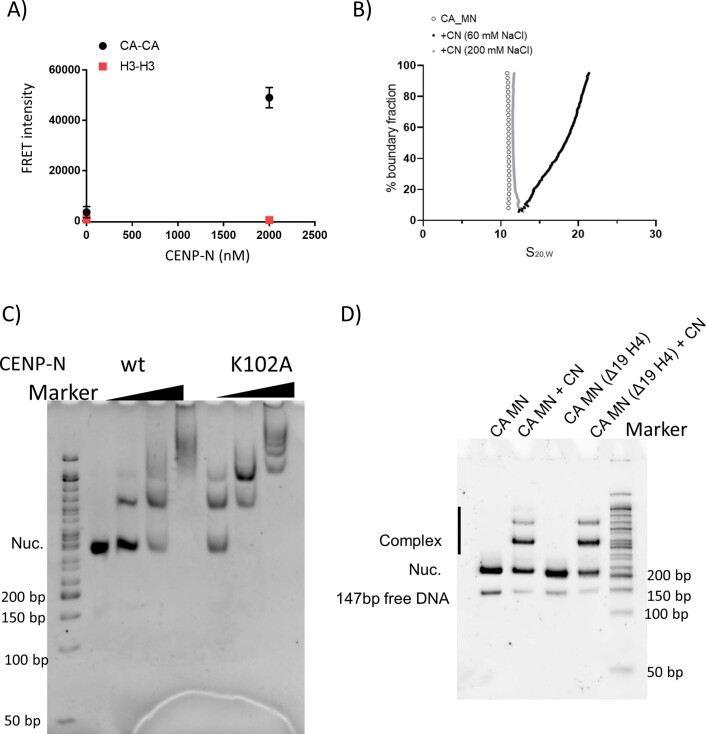
Solution assays for CENP-N induced CENP-A nucleosome stacking. **a)** FRET analysis of homotypic CENP-A mono-nucleosome (CA) or H3 mono-nucleosome (H3) interactions in the absence or presence of CENP-N. Donor is a mono-nucleosomes containing Alexa 488 labeled H2B; Acceptor is a mono-nucleosome containing Atto N 647 labeled H2B. 250 nM donor and acceptor nucleosome; FRET intensity in dependence of [CENP-N]. Final concentration for NaCl is around 100 mM. Error bars from three independent measurements. Data are presented as mean values + /- SD. **b)** Salt concentration affects nucleosome stack formation. AUC analysis (van Holde-Weischet plots) of CENP-N in complex with CENP-A mono-nucleosomes at 60 and 200 mM NaCl). CN: CENP-N^1–289^ in complex with CENP-A mono-nucleosome. CA_MN: CENP-A mono-nucleosome. **c)** CENP-N mutant (K102A) binds to CENP-A nucleosomes as well as wild-type CENP-N (5% native PAGE). 250 nM CENP-A nucleosome was combined with CENP-N at ratios of 2:1, 4:1 and 8:1 in buffer containing 50 mM NaCl, 20 mM Tris-HCl (pH 7.8), 1 mM EDTA, 1 mM DTT. **d)** deletion of the H4 N-terminal tail (∆19) does not affect the specific interaction between CENP-N and CENP-A nucleosomes. CENP-N was mixed with CENP-A nucleosome containing full length H4 or (∆19) H4 at a 2:1 ratio in the same buffer as in A). Source data

To confirm that nucleosome stacks are not artifacts of cryo-EM grid preparation, we analyzed CENP-A nucleosomes in the absence and presence of CENP-N by sedimentation-velocity analytical ultracentrifugation (SV-AUC) under the buffer conditions used for cryo-EM, but at much lower nucleosome concentrations (250 nM, compared with the µM concentrations required for cryo-EM). In the absence of CENP-N, CENP-A mononucleosomes sediment homogeneously with a sedimentation coefficient (*S*_(20,W)_) of ~10.5 S (Fig. 1c), consistent with reported values for canonical nucleosomes^25^. In the presence of CENP-N^1–289^, CENP-A nucleosomes assemble into much larger and more heterogeneous species, as evident by a *S*_(20,W)_ value ranging from 13 S to 30 S. For reference, dinucleosomes and 12mer nucleosomal arrays (containing 12 repeats of nucleosomes on one DNA template) sediment at 13 S and 30 S, respectively (unpublished data and ref. ^26^). When CENP-N was combined with nucleosomes containing H3, no larger species were observed upon addition of CENP-N (Fig. 1c). To analyze the effect of CENP-N in a more physiologically relevant context, we showed that full-length CENP-N in complex with CENP-L bound to a CENP-A nucleosome under the same conditions also promotes the oligomerization of the CENP-A nucleosome (Fig. 1c). To further confirm that nucleosomes indeed come in close contact in the presence of CENP-N, we designed a Foerster resonance energy transfer (FRET) assay. CENP-A nucleosome containing Alexa 488-labeled H2B was the donor, and CENP-A nucleosome containing Atto 647N-labeled H2B was the acceptor. Nucleosome-nucleosome interactions should result in a strong FRET signal, and indeed we observed an increase in FRET upon titrating CENP-N into an equimolar mixture of donor and acceptor nucleosomes (Fig. 1d). No FRET signal was observed withH3 nucleosomes and CENP-N (Extended Data Fig. 5a). Collectively, our data show that CENP-N mediates the stacking of mononucleosomes by engaging simultaneously with two CENP-A nucleosomes.

### CENP-N α6 interacts with the DNA of a neighboring nucleosome to promote nucleosome stacking in vitro

**Fig. 2:**
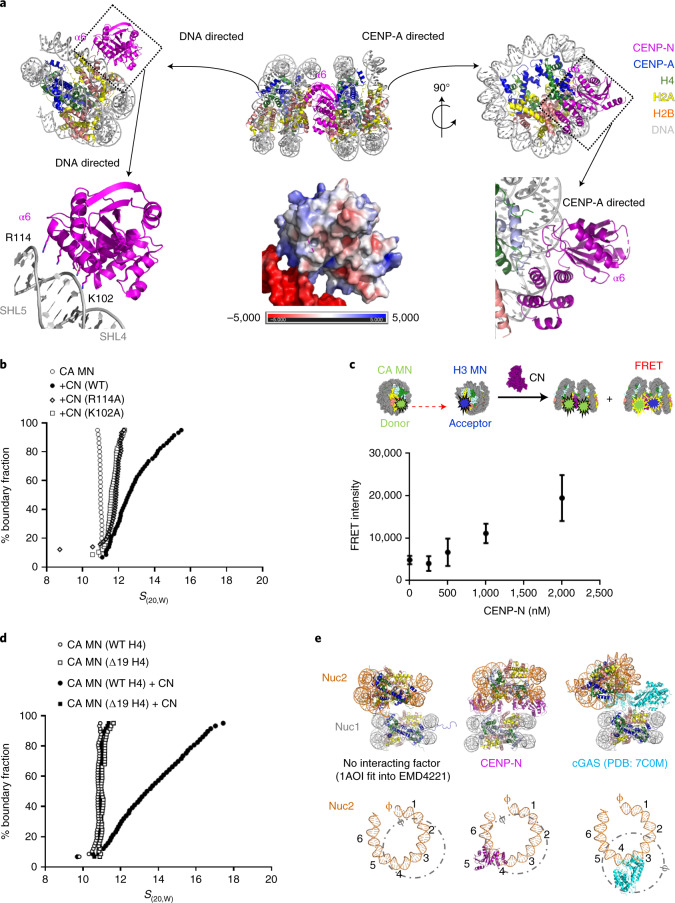
Structural basis for CENP-N-dependent nucleosome-nucleosome interaction. **a**, The CENP-N α6 helix interacts with nucleosomal DNA of a second nucleosome without contacting histones (nonspecific nucleosome). Top, overview of interactions. Lower left, the interface between CENP-N α6 and DNA is highlighted. Lower middle, a surface charge representation (unit, kT e^−1^) in the same orientation. Lower right, the specific interaction on the other side of CENP-N with the CENP-A RG loop. **b**, Single mutations on CENP-N α6 affect nucleosome-nucleosome interactions, as shown by AUC. **c**, FRET analysis of the interaction between CENP-A mononucleosome (CA MN) and H3 mononucleosome (H3 MN) in the absence or presence of CENP-N. The donor is CENP-A mononucleosomes containing Alexa 488-labeled H2B; the acceptor is a H3 mononucleosome containing Atto N 647-labeled H2B (250 nM donor and acceptor nucleosome were used); FRET intensity changes in dependence of [CENP-N]. Error bars are from five independent measurements of two biological replicates. Data are presented as mean values ± s.d. **d**, The H4 N-terminal tail is essential for CENP-N-mediated nucleosome stacking (van Holde–Weischet plots of sedimentation). ∆19 indicates H4 tail deletion (amino acids 1–18). **e**, Comparison of different modes of nucleosome stacking. ‘Nuc1’ represents the reference nucleosome that interacts specifically with the indicated factor. ‘Nuc2’ is the neighboring nucleosome which interacts with Nuc1 or its binding factors non-specifically. Top, models for stacked mononucleosomes. 1AOI is the PDB code for a previously published nucleosome structure. Bottom, ‘superhelix locations’ (SHLs) (1-6) and the nucleosomal dyad axis (SHL 0; ɸ) of nuc2 (brown color, DNA only), are indicated, with nuc1 shown in a dotted circle (gray color), depicts the relative orientations of nuc1 and nuc2 and CENP-N or cGAS, respectively. Only half of the nucleosomal DNA is shown for clarity. Source data

**Extended Data Fig. 6:**
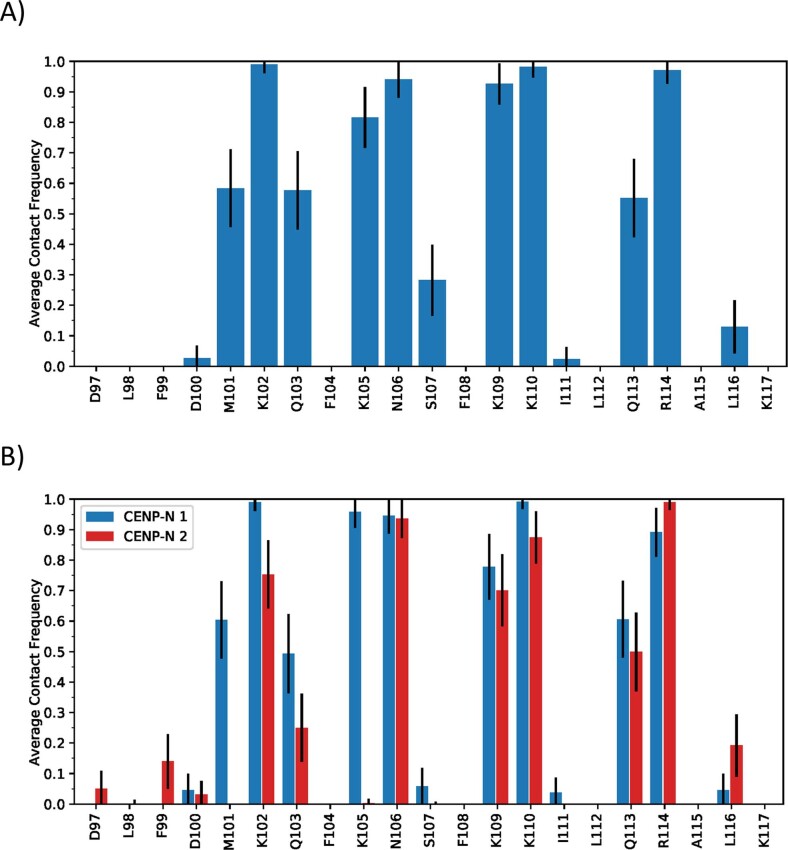
Residue contacts of the CENP-N α6-helix with DNA of the DNA-directed nucleosome from simulations containing one (A) and two CENP-N (B). CENP-N 1 and CENP-N 2 are distinguished by the binding orientation of the α6-helix with the DNA grooves. CENP-N 1 (blue) binds directly into the DNA minor groove while CENP-2 (red) does not. Protein-DNA contacts were defined between the heavy atoms of residues within 4.0 Å of one another. Standard errors were derived using n = 15, where n is the number of statistically independent data points in each window as was determined by calculating the statistical inefficiency.

CENP-N specifically binds to the CENP-A nucleosome through recognizing the RG loop on CENP-A by its α1 helix and β3–β4 loop (Fig. 2a). How does CENP-N interact with a second nucleosome? Our structures reveal a previously unidentified interface between CENP-N and nucleosomal DNA, consisting of a series of positively charged amino acids (K102, K105, K109, K110, R114, and K117) that are all located on the same face of the α6 helix of CENP-N, on the opposite side of the main CENP-A decoding interface on CENP-N. These side chains allow α6 to dock onto super helical location (SHL) 4–5 of the second nucleosome in the stack (Fig. 2a). Consistent with the electrostatic nature of this interface, nucleosome-stack formation is strongly affected by ionic strength (Extended Data Fig. 5b). When the salt concentration is elevated to 200 mM, CENP-N is still able to interact with the CENP-A nucleosome, but no stack formation is observed. Point mutation of individual side chains (K102A or R114A) resulted in reduced levels of nucleosome stacking (Fig. 2b). Neither of these side chains is in the interface involved in specific recognition of CENP-A, and as expected, a gel shift assay showed no difference in binding to CENP-A mono-nucleosomes made with CENP-N containing the K102A mutation (Extended Data Fig. 5c). This is consistent with our MD simulations, which demonstrate that the α6 helix (in particular the amino acids listed above) form strong contacts with the neighboring DNA (Extended Data Fig. 6). The charged face of the α6 helix is not conserved in CENP-N from fungi with point centromeres, and this may reflect the dispensability of an additional bridging interface between nucleosomes in a point centromere compared with a regional centromere^27,28^.

Since one CENP-N is sufficient to mediate nucleosome-stack formation, and since the second nucleosome interacts with CENP-N through its DNA, it could, in theory, also promote stacking between CENP-A and H3 nucleosomes. To test this, we performed FRET experiments with CENP-A and H3 nucleosomes labeled with fluorescence donor and acceptor, respectively. Pronounced FRET signal was observed between the CENP-A nucleosome and H3 nucleosome with increasing CENP-N concentrations, confirming our prediction (Fig. 2c). Since only one CENP-N can bind between a CENP-A nucleosome and an H3 nucleosome (whereas two CENP-N can be placed between two CENP-A nucleosomes; Fig. 1a), the FRET signal is weaker than that observed for two CENP-A nucleosomes.

Histone tails, especially the H4 N-terminal tail, contribute to chromatin compaction (for example, refs. ^29–32^). Because CENP-N appears to redirect the H4 tail^16^, we tested by AUC whether the H4 tail contributes to CENP-N-mediated nucleosome stacking. We prepared CENP-A-containing nucleosomes in which the H4 N-terminal tail was deleted (Δ19H4). No CENP-N-dependent oligomerization was observed for these nucleosomes (Fig. 2d), even though they bind to CENP-N as well as CENP-A nucleosomes containing full-length H4 tails (Extended Data Fig. 5d).

Nucleosome-nucleosome interactions have been observed previously. For example, major-type nucleosomes form several types of dinucleosomes on cryo-EM grids in the absence of any interacting protein^33^ (Fig. 2e), likely mediated through histone tails. Recent cryo-EM structures of cGAS (a protein that senses the presence of cytoplasmic DNA during the innate immunity response) in complex with nucleosomes show that it bridges two mononucleosomes. cGAS binds to one nucleosome by interacting with the surface of histones H2A-H2B and nearby DNA, while a positively charged α-helix interacts with DNA of the second nucleosome (Fig. 2e)^34–36^. A caveat here is that the existence of cGAS-mediated nucleosome stacks was not verified in solution. Of note, the relative orientation of the nucleosomes is quite different in the three arrangements (the second nucleosome indicated by dashed circles in Fig. 2e), enforcing the concept gained from nucleosome crystallography that there are many ways to pack nucleosomes in an energetically favorable way (for example, ref. ^37^).

### CENP-N folds and twists CENP-A-containing chromatin arrays

**Fig. 3:**
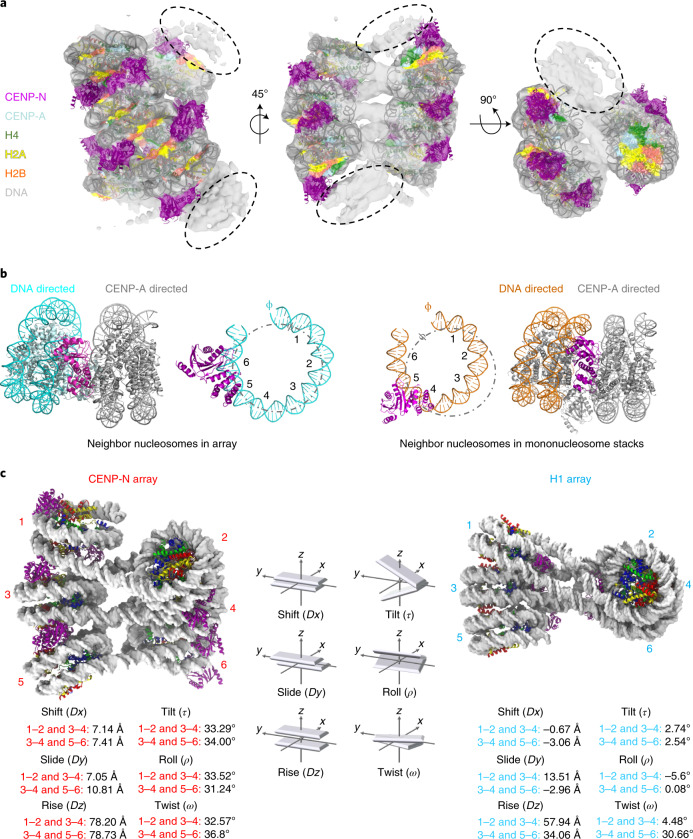
CENP-N folds CENP-A chromatin into a regular fiber. **a**, A nucleosomal array consisting of six CENP-A nucleosomes with CENP-N was fit into the density map. Dashed circles indicate weak densities representing more disordered nucleosomes at both ends. **b**, CENP-N interacts with nucleosomal DNA of a neighboring nucleosome (CENP-N α6 binds between SHL6 and 7 (left)). This is in contrast to mononucleosome stacks, where CENP-N α6 binds between SHL4 to 5 (right). **c**, The relative arrangement of nucleosomes in the arrays was expressed in analogy to the arrangement of DNA bases in a double helix. Parameters for CENP-N fibers (left, EMD-26333) and H1 fibers^32^ are shown. The vectors in the middle show the parameters of the analysis (in red for CENP-A arrays, and cyan for H1 arrays).

**Extended Data Fig. 7:**
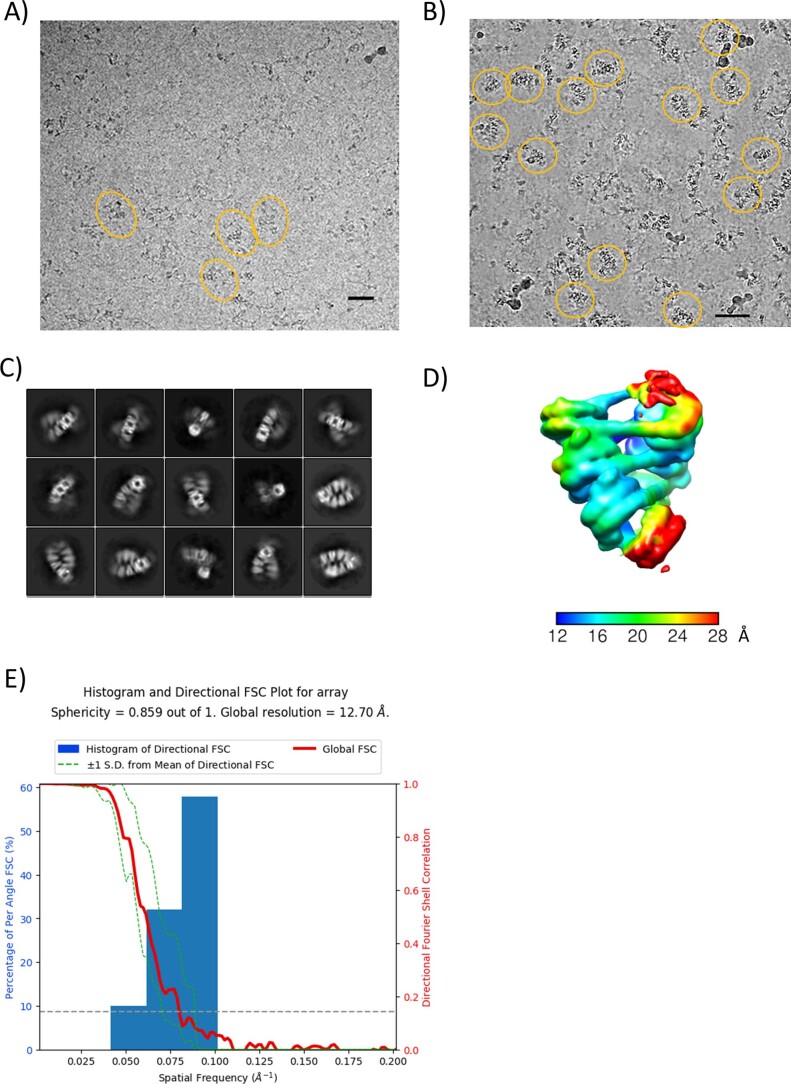
CryoEM analysis of 12mer nucleosomal arrays in presence of CENP-N. **a)** raw cryoEM image of CENP-N in complex with 12-207mer 601 array. Size bar is 50 nm. **b)** raw cryoEM image of CENP-N in complex with 12-167mer array. Size bar is 50 nm. **c**) 2D classification of CENP-N in complex with 12-167mer array. **d)** Local resolution map of chromatin fiber. Color key indicates the resolution. **e)** 3DFSC analysis of cryoEM electron map.

The interactions between mononucleosomes observed in vitro might reflect how nucleosomes form long-range interactions in vivo without constraints from connecting DNA. We next asked whether CENP-N promotes the short-range interactions required to form chromatin fibers from a linear nucleosomal array. CENP-N^1–289^ was mixed with CENP-A-containing arrays assembled onto 12 tandem repeats of 207 or 167 bp 601 DNA (12–207 and 12–167, respectively), at a ratio of 5 CENP-N^1–289^ per nucleosome to reach saturation. Cryo-EM images show that both chromatin arrays fold into twisted zig-zag chromatin fibers (Extended Data Fig. 7a,b). This type of folding is usually observed only in the presence of divalent cations or upon addition of linker histone to canonical H3 arrays^32,38^. Whereas the longer linker segments in 12–207 nucleosomal arrays introduced too much variability to allow structure determination, we were able to determine the ~12.7-Å structure of the more constrained CENP-A 12–167 array in complex with CENP-N (Fig. 3a). Of note, the average linker length at the centromere is ~25 bp^39^, close to the linker length of ~20 bp used here. It bears pointing out that, although linker length affects fiber geometry, the relative orientation of the n and n + 2 nucleosomes in a two-start helix is not expected to be affected in a major way by DNA linker length^40^. Eight nucleosomes, each bound by two CENP-N molecules, were observed in the density map; the two terminal nucleosomes on either end were too flexible to be described with any certainty.

The nucleosome arrangement takes the form of a two-start twisted double helix with two CENP-N bridging the n and n + 2 nucleosomes. CENP-N is in its previously described location on the CENP-A nucleosome but binds SHL 6 and 7 of the n + 2 nucleosome, rather than SHL 4 and 5 as observed in mononucleosome stacks (Fig. 3b). This results in a different relative orientation of the n and n + 2 nucleosome stack compared with that formed from mononucleosomes, and provides evidence for the plasticity of the interaction between α6 and nucleosomal DNA.

**Extended Data Fig. 8:**
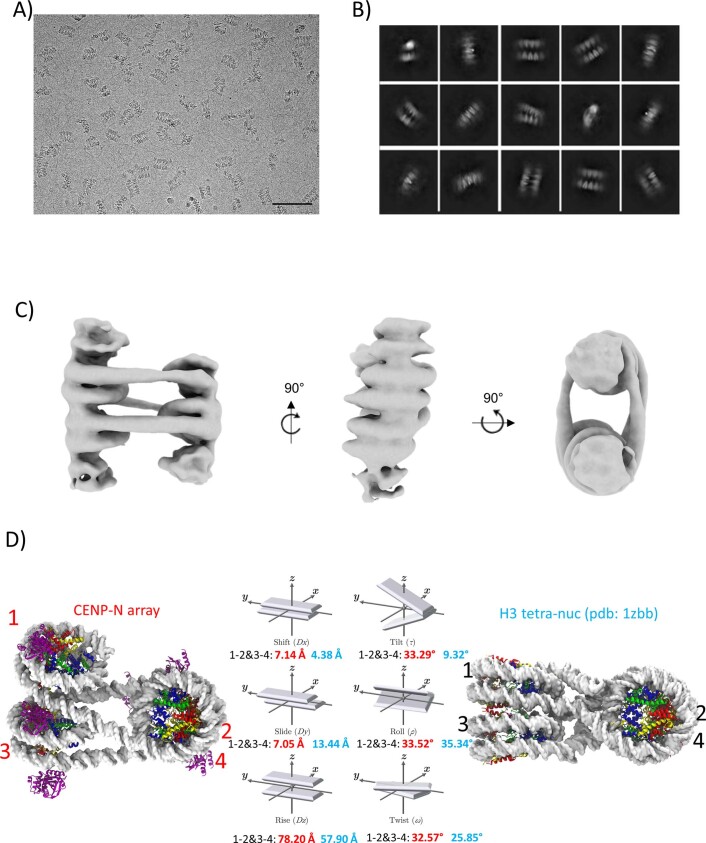
CENP-N affects the folding of chromatin arrays. **a)** Comparison between tetra-nucleosomes in a chromatin fiber with CENP-N, and of a canonical tetra-nucleosome. Analysis of the relative orientation of the nucleosomes, in analogy to DNA base pair analysis, is shown in the middle panel, with numbers for the CENP-N array in red, and tetranucleosome array in black. **b)** CryoEM analysis of crosslinked chromatin arrays without CENP-N, raw cryoEM image. Size bar is 100 nm **c)** 2D classification of CENP-N in complex with 12-167mer array. **d)** Low resolution 3D cryoEM map illustrates ladder-like arrangement of nucleosomes.

Linker histone H1 (which binds to the nucleosomal dyad and linker DNA of canonical nucleosomes^41^) stabilizes compact chromatin states^42^. Although the manners in which CENP-N and H1 interact with nucleosomes are completely different, they both promote chromatin fibers with superficially similar two-start zig-zag architectures held together by the stacking of n and n + 2 nucleosomes (Fig. 3c). However, the CENP-N–CENP-A chromatin fiber exhibits features that distinguish it from the H1-induced fiber. A larger distance and angle between the n and n + 2 nucleosomes are required to accommodate CENP-N. This leads to a steeper twist of the fiber (Fig. 3c). Additionally, the chromatin fiber formed with H1 exhibits a discrete tetra-nucleosomal structural unit, a repeat of four nucleosomes^32^, while the organization of CENP-A chromatin fibers with CENP-N is continuous. Of note, the packing of n and n + 2 CENP-A nucleosomes in the presence of CENP-N also differs from the nucleosome interactions observed in the crystal structure of a canonical tetranucleosome stack^38^ (Extended Data Fig. 8a).

CENP-A nucleosomes are characterized by less-tightly-bound DNA ends, which affects the geometry of CENP-A-containing chromatin^10^. In the presence of CENP-N, all CENP-A nucleosomes (both in mononucleosome stacks and in folded chromatin arrays) exhibit tightly bound DNA ends, similar to what is observed for canonical nucleosomes (this study and ref. ^16^), and in this stabilization of the terminal turns of nucleosomal DNA, CENP-N also functionally resembles linker histone H1. Overall, our data suggest that CENP-N, as one of the key proteins of the inner kinetochore, stabilizes, organizes, and compacts centromeric chromatin in a way that depends on its specific interaction with CENP-A nucleosomes and on its DNA-directed interactions with a neighboring nucleosome.

We observed a structural change in nucleosomal arrays by cryo-EM when CENP-N was lost in a buffer containing 200 mM NaCl during overnight sucrose gradient centrifugation (Extended Data Fig. 8b–d). In MD simulations, chromatin converts to a parallel ladder-like structure when H1 is removed from the simulation^43^. Ladder-like structures of CENP-A chromatin arrays have also been observed under certain conditions, for example in the presence of divalent ions^44^, illustrating the ability of chromatin arrays to assume different arrangements depending on conditions. As such, the structural transition caused by CENP-N in CENP-A arrays is similar (although distinct) from that caused by H1 on canonical chromatin. Intriguingly, H1 is unable to bind to CENP-A nucleosomes in vitro and in vivo^11,12^, and we speculate that CENP-N might take over the role of H1 in closely packing CENP-A nucleosomes with surrounding nucleosomes.

### CENP-N promotes the compaction of centromeric chromatin in vivo

To explore the role of CENP-N in the compaction of centromeric chromatin in vivo, we used sucrose gradient ultracentrifugation to fractionate and separate mechanically sheared cross-linked chromatin isolated from cells. As shown previously, chromatin domains with higher levels of compaction (for example, heterochromatin) are more resistant to sonication than is the more open euchromatin, and thus sucrose gradient ultracentrifugation enables separation of these different chromatin states. The sonication-resistant, more compact chromatin migrates faster and sediments in fractions of high sucrose density^45,46^, whereas more open chromatin migrates slower and fractionates at lower sucrose density. It has been observed that active promoters are enriched in chromatin fractions of low resistance to sonication, which is the basis of the techniques Sono-seq^46^ and formaldehyde-assisted isolation of regulatory elements (FAIRE)^47^. The method has also been used for mapping of heterochromatic regions across the genome^45^. This approach provides a unique assay for measuring the compaction of centromeric chromatin.

**Fig. 4:**
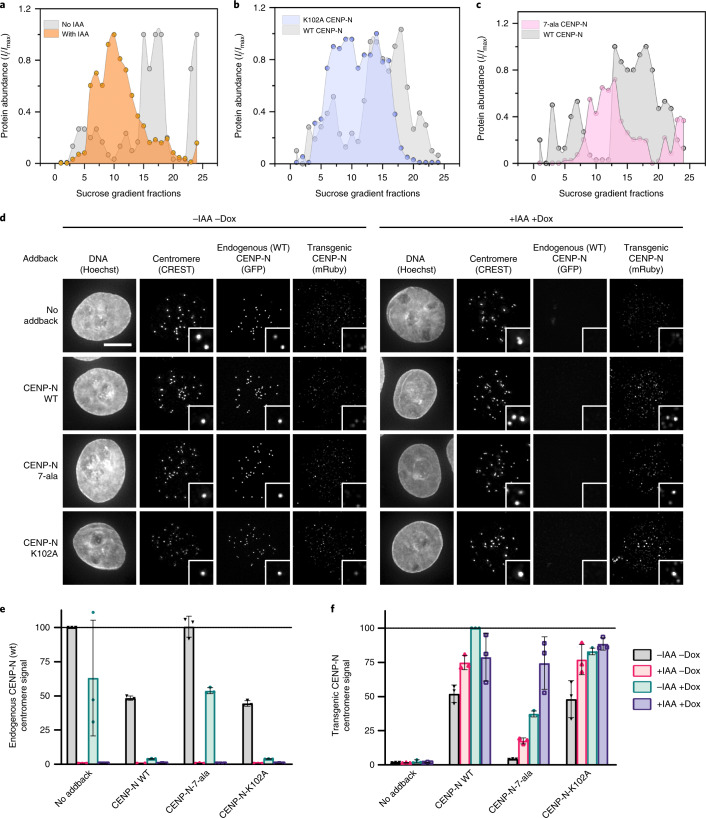
CENP-N promotes the compaction of centromeric chromatin in vivo. **a**, CENP-A distribution in sucrose gradient in the presence of WT CENP-N (gray) and after IAA-induced degradation of CENP-N (orange). An equal amount of chromatin was loaded per well on an SDS gel, resolved by blotting against H4 histone (Extended Data Fig. 9l). **b**,**c**, Comparison of CENP-A distribution in the presence of transiently expressed CENP-N variants, WT CENP-N (gray), K102A CENP-N (lilac), and 7-ala CENP-N (pink, 7 positively charged amino acids on α6 are mutated to alanine). Dots in **a**–**c** represent a mean of two biological replicates. I_max_ and I_i_ in **a**–**c** represent a maximum signal intensity from all fractions and the signal intensity in a given fraction, respectively. Solid lines represent interpolation between experimental data points (using Akima spline). **d**, Representative images of nuclei (Hoechst stain) showing endogenous WT CENP-N (GFP) or transgenic CENP-N variant (anti-Ruby antibody) localization at centromeres (CREST signal). Scale bar, 5 µm. Insets show magnified images of example centromeres (CREST foci). Cells were treated for 24 hours with doxycycline (dox) to induce expression of mRuby2-3×FLAG-tagged CENP-N (WT or mutant) and/or with indole acetic acid (IAA) to deplete endogenous auxin-inducible degron (AID)-tagged CENP-N-GFP as indicated. **e**,**f**, Normalized centromeric fluorescence signal corresponding to endogenous WT CENP-N (GFP) (**e**) or transgenic WT or CENP-N variant (anti-mRuby antibody) (**f**). Each dot represents the median centromeric CENP-N signal from >100 cells from 1 biological replicate. Line and error bars are mean of three biological replicates ± s.d. Source data

**Extended Data Fig. 9:**
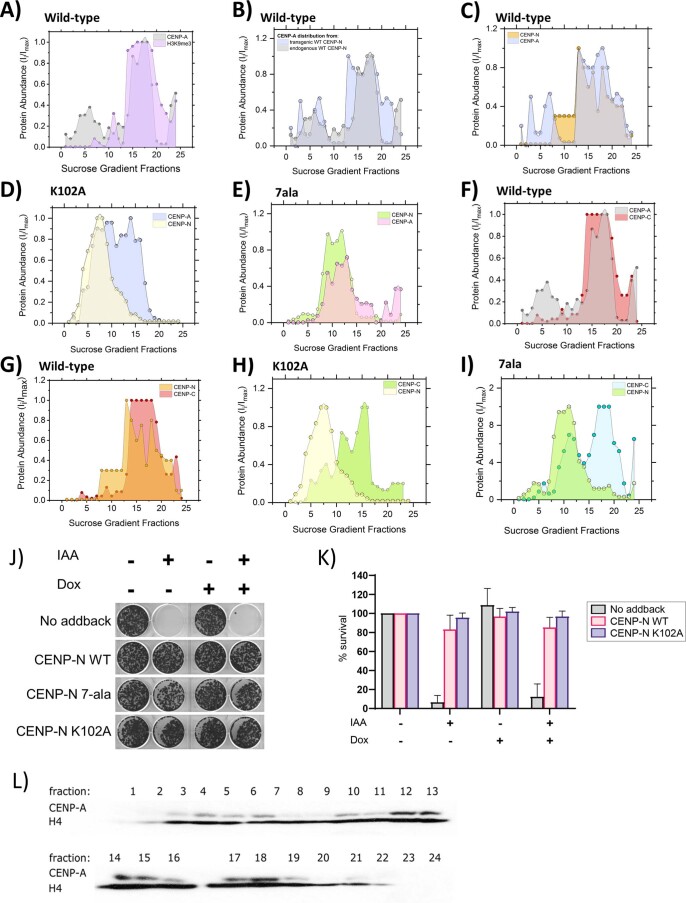
Western blot detection of centromeric proteins across 5-40% sucrose gradients and clonogenic survival assay. **a)** Comparison of CENP-A (gray) and H3K9me3 (lilac) distribution in the presence of endogenous WT-CENP-N. **b)** CENP-A distribution in the presence of endogenous (gray) and transgenic (blue) WT-CENP-N. **c)** Comparison of CENP-A (blue) and CENP-N (orange) in the presence of WT CENP-N. **d)** Comparison of CENP-A (blue) and CENP-N (yellow) in the presence of K102A CENP-N. **e)**. Comparison of CENP-A (pink) and CENP-N (green) distribution in the presence of 7ala CENP-N. **f)** Comparison of CENP-A (gray) and CENP-C (red) distribution in the presence of endogenous WT-CENP-N. **g)** CENP-N (orange) and CENP-C (red) distribution in the presence of endogenous WT-CENP-N **h**) CENP-N (yellow) and CENP-C (green) distribution in the presence of K102A CENP-N. **i**) CENP-N (yellow) and CENP-C (green) distribution in the presence of 7ala CENP-N. **j)** Representative crystal violet-stained colonies from clonogenic survival assay showing viable CENP-N AID cells with the indicated transgenic CENP-N variant treated with or without 0.1 mM IAA and doxycycline for 14 days. After treatment of 1000 seeded cells/well, surviving colonies were fixed and stained with crystal violet stain. **k)** Quantification of average percentage survival (average crystal violet stain intensity) of CENP-N AID cells (except CENP-N 7-ala) from 3 biological replicates normalized to untreated cells. (CENP-N 7-ala survival data is from single replicate). Data are presented as mean values± SD. **l)** Western blot of CENP-A and H4 histone distribution across 5–40 % sucrose gradient. Presence of CENP-A signal indicates fractions containing the centromeric chromatin whereas H4 signal represents the overall amount of chromatin loaded onto a SDS gel. Source data

**Extended Data Fig. 10:**
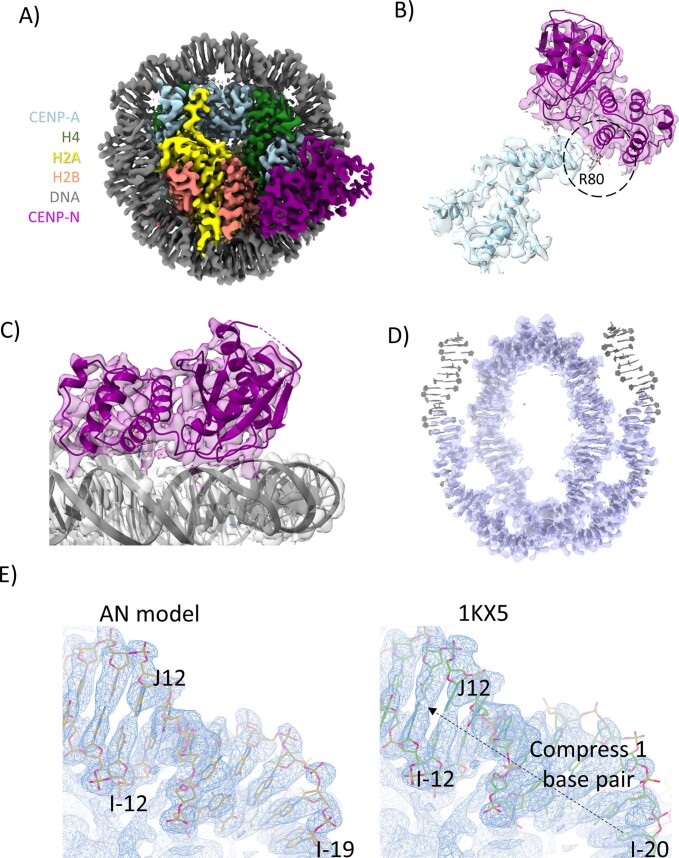
The structure of CENP-A α satellite nucleosome in complex with CENP-N^NT^. **a)** 2.65 Å cryoEM map of CENP-A α satellite nucleosome in complex with CENP-N^NT^. The components are colored as indicated. **b) D**ensity (with model) of the interface between CENP-N and histones. **c)** Density (with model) of the interface between CENP-N and nearby DNA. **d)** The model to density of DNA. **e)** α satellite DNA is compressed by one base pair in the crystal structure of the nucleosome (pdb 1KX5), compared to our cryoEM structure where DNA ends are unconstrained.

We used a 5–40% sucrose gradient and identified the fractions containing centromeric chromatin with antibodies against CENP-A, CENP-N, and CENP-C in western blots. Centromeric chromatin (anti hCENP-A antibody signal) sediments in high-density sucrose fractions (for example, 12–20) (Fig. 4a, shown in gray). These same fractions also contain highly compacted heterochromatin, as they also stain with antibodies against H3 trimethylated at K9 (H3K9me3), a marker for constitutive heterochromatin (Extended Data Fig. 9a)^48–50^. This suggests that centromeric chromatin indeed resists sonication just like heterochromatin, reflecting a high level of compaction.

To assess the role of CENP-N in compacting CENP-A nucleosomes in vivo, we endogenously tagged both alleles of CENP-N with the auxin-inducible degron (AID) tag in cells expressing the F-box protein Tir1. This AID system enables targeting CENP-N for degradation upon the addition of auxin^18^, and this causes depletion of CENP-N from centromeres (Fig. 4d,e) and a reduction in long-term cell viability (Extended Data Fig. 9j–k). Transient degradation of CENP-N for 30 minutes caused pronounced changes in the migration of centromeric chromatin in sucrose gradients, assayed by CENP-A distribution. Most CENP-A chromatin from these cell lines now migrates with lower-density sucrose gradients (fractions 5–15, Fig. 4a, in orange), indicating that the loss of CENP-N renders centromeric chromatin more accessible to shearing by sonication. We tested whether transgenic expression of CENP-N rescues the effects of endogenous CENP-N degradation on CENP-A chromatin migration by introducing mRuby2-3×FLAG-tagged full-length CENP-N into AID-tagged CENP-N cells as a transgene (transgene wild-type (WT) CENP-N) under doxycycline induction. Upon doxycycline addition, we observed localization of transgenic CENP-N at centromeres by immunofluorescence using anti-Ruby antibody in cells depleted of endogenous CENP-N (Fig. 4d–f). Furthermore, transgenic CENP-N WT could rescue loss of cell viability of CENP-N AID cells (Extended Data Fig. 9j,k). Complementation of AID-CENP-N loss through WT CENP-N expression also rescues the migration of CENP-A nucleosomes in sucrose gradients to what is observed in unmanipulated cells (Extended Data Fig. 9b). The distribution of transgenic CENP-N in the gradient overlapped with the distribution of the CENP-A nucleosomes, indicating that transgenic CENP-N associates with CENP-A chromatin (Extended Data Fig. 9c).

We next tested the contribution of the α6 helix of CENP-N in the compaction of centromeric chromatin by complementing AID-CENP-N degradation with transgenic expression of mRuby2-3×FLAG-tagged K102A mutant of CENP-N or a mutant of CENP-N in which seven positively charged residues in the α6 helix were mutated to alanines (7-ala). Upon doxycycline addition, CENP-N-K102A or CENP-N-7-ala localized to centromeres in cells depleted of endogenous CENP-N (Fig. 4d–f), indicating that CENP-N with a mutated α6 helix retains the ability to target to CENP-A chromatin in cells. However, we observed that the 7-ala mutant localization was reduced in the presence of endogenous CENP-N (Fig. 4f, –IAA +Dox), suggesting reduced protein stability and/or CCAN interactions compared with WT CENP-N. Basal expression of the K102A mutant, in the absence of doxycycline induction, competed for the endogenous CENP-N and fully displaced endogenous CENP-N from centromeres upon doxycycline induction (Fig. 4e, –IAA –Dox, –IAA +Dox). This demonstrates that the nucleosome-binding activity of the K102A mutant efficiently competes for the limited number of CENP-N-binding sites at centromeres despite its inability to condense centromeric chromatin. Similar to what was observed for transgenic expression of WT CENP-N, transgenic expression of CENP-N-K102A and CENP-N-7-ala restored long-term viability in cells depleted of endogenous CENP-N (Extended Data Fig. 9j,k), indicating that the function of the kinetochore for chromosome segregation is intact in these mutants. This is in contrast to mutations in the CENP-N–CENP-A histone interface, which perturb CENP-N localization and cell viability^16,51^. However, complementation of AID-CENP-N depletion with the CENP-N α6 mutant did not rescue the increased susceptibility of centromeric chromatin to shearing caused by the degradation of endogenous WT CENP-N. Most CENP-A-containing chromatin was localized in fractions 5–15 in the presence of the CENP-N α6 mutants as compared with fractions 12–20 in the presence of WT CENP-N (Fig. 4a–c). We confirmed that transgenic CENP-N mutant proteins were indeed expressed, and that their distribution within the sucrose gradient overlaps with the corresponding CENP-A distribution (Extended Data Fig. 9d–f).

Like CENP-N, CENP-C also directly binds to CENP-A nucleosomes^51^. We found that the distribution of CENP-C in sucrose gradients is different in cell lines expressing CENP-N mutants compared with cell lines with WT CENP-N (Extended Data Fig. 9g–i). CENP-C migrated with low-density sucrose fractions that contain CENP-A and K102A CENP-N, but we also detected CENP-C in high-density sucrose fractions which were depleted in CENP-N but not in CENP-A (Extended Data Fig. 9h). These results suggest that there is a population of CENP-A nucleosomes not bound by CENP-N that sediments with compacted centromeric chromatin (for example, high-density sucrose fractions, Extended Data Fig. 9d,h, fractions 15–20) and that this compaction state corresponds to the presence of the CENP-C protein. Altogether, our in vivo studies validate the in vitro results to demonstrate that CENP-N plays an important role in compaction of CENP-A and H3 chromatin through its α-6 helix.

## Discussion

The specialized chromatin structure at the centromere, which is necessary for the assembly of the kinetochore, is controlled by two proteins (CENP-N and CENP-C) that specifically interact with CENP-A-containing nucleosomes. CENP-A nucleosomes are found only at centromeres, where they are interspersed with H3 nucleosomes along the centromeric DNA, but clustered in 3D. CENP-N promotes the stacking of nucleosomes in vitro and in vivo through a previously undescribed DNA interaction interface, and this has important implications for our understanding of higher order structure at the centromere and for the transmission of force on chromosomes exerted by mitotic spindle fibers. Our finding that the H4 tail contributes to nucleosome-nucleosome interactions is underscored by the fact that histone H4 at the centromere is not acetylated in its tail regions^52^. This is notable because previous data showed that acetylation of H4 at K16 precludes the formation of higher order structure in vitro and in vivo^53^. In addition, the H4 N-terminal tail trajectory is altered upon its interaction with CENP-N^16^, which could promote its interaction with either the acidic patch or DNA of an adjacent nucleosome.

The interactions between CENP-N and the CENP-A nucleosome that we observe here and in refs. ^16,17,22^ are different from those previously described for the yeast CCAN complex^28,27^ and two recent structures of the human CCAN complex^54,55^. No CENP-A-directed interaction with CENP-N was found in the yeast or human complex, unlike what has been demonstrated in three published structures of human CENP-N–CENP-A nucleosome complexes. Instead, a positively charged channel formed by the CENP-N/L dimer contacts DNA exiting from the nucleosome. This channel is independent of the α6 helix, which is instead positioned away from the CENP-A nucleosome. This suggests two models for the role of the α6 helix. In the first model, CENP-N incorporated into the CCAN provides a key structural element for binding of the DNA of the CENP-A nucleosome, while the α6 helix positioned away from the CENP-A nucleosome is available to interact with another CENP-A or H3 nucleosome. Alternatively, CENP-N may have two functions, one as a structural component of the CCAN that binds nucleosomal DNA through the CENP-N/L dimer and a second that bridges CENP-A or CENP-A and H3 nucleosomes that are distinct from those nucleosomes recognized by the CCAN. Either model for CENP-N could provide a condensation activity for centromeric chromatin. The α6 helix is not conserved between human and yeast, especially the positively charged residues that are implicated in the interaction with the second nucleosome in human CENP-N. This could reflect an evolutionary adaptation for stabilizing point versus regional centromeres.

While CENP-N is specific for CENP-A nucleosomes^56^, the interaction with the neighboring nucleosome is promiscuous with respect to histone content and DNA sequence and location. Thus, CENP-N can promote the close packing of CENP-A nucleosome and various surrounding nucleosomes, even including sub-nucleosomes (hexasomes or tetrasomes). CENP-N promotes the formation of stacks from mononucleosomes (reflecting the interaction of unconnected nucleosomes from different regions of the genome), as well as nucleosomal arrays, where it leads to the formation of a zig-zag two-start helix with a topology that is distinct from the canonical nucleosome fiber formed by linker histone H1. This is important as H1 is unable to bind to CENP-A nucleosomes. It is therefore possible that CENP-N acts as a centromere-specific ‘linker histone’ to promote the formation of centromere-specific chromatin higher order structure, which in turn serves as an interaction platform for a plethora of additional centromere-specific proteins.

Chromatin higher order structures are heterogeneous in vivo and can be influenced by a variety of factors. Linker histone H1 compacts chromatin by organizing extranucleosomal linker DNA. Heterochromatin protein-1 (HP1) promotes heterochromatin formation through reading the histone modification H3K9me3 as well as self-dimerization^57^. Here we report yet another chromatin compaction mechanism where CENP-N specifically reads the histone variant content of one nucleosome while interacting with the DNA of a neighboring nucleosome, to potentially form unique compact chromatin structures at the centromere.

### Reporting Summary

Further information on research design is available in the Nature Research Reporting Summary linked to this article.

## Online content

Any methods, additional references, Nature Research reporting summaries, source data, extended data, supplementary information, acknowledgements, peer review information; details of author contributions and competing interests; and statements of data and code availability are available at 10.1038/s41594-022-00758-y.

## Supplementary information

Supplementary Information Materials and Methods, Supplementary Note, legends for Extended Data figures and legends for Videos 1–3.

Reporting Summary

Supplementary Video 1 Simulation of the stacked-nucleosomes bound to two CENP-N. Shown is the DNA (white), CENP-N (purple), histone H3 (blue), histone H4 (green), histone H2A (yellow), and histone H2B (red). The simulation time evolution is presented in the bottom right-hand corner of the video.

Supplementary Video 2 Simulation of the stacked-nucleosomes bound to one CENP-N. Shown is the DNA (white), CENP-N (purple), histone H3 (blue), histone H4 (green), histone H2A (yellow), and histone H2B (red). The simulation time evolution is presented in the bottom right-hand corner of the video.

Supplementary Video 3 Simulation of the stacked-nucleosomes not bound to any CENP-N. Shown is the DNA (white), histone H3 (blue), histone H4 (green), histone H2A (yellow), and histone H2B (red). The simulation time evolution is presented in the bottom right-hand corner of the video.

## Data Availability

Cryo-EM maps have been deposited with the EMDB (EMD-26330; EMD-26331; EMD-26332; EMD-26333). PDB files of mono- and dinucleosome structures (with appropriate validation) have been deposited with the PDB (7U46, 7U47, 7U4D). Simulation trajectories are available on request. Source data for Figs. 1, 2 and 4, and Extended Data Figs. 5 and 9 are available with the paper on line. Source data are provided with this paper.

## Source data

Source Data Fig. 1 Raw statistical data for the plots in Fig1c,d
Source Data Fig. 2 Raw statistical data for the plots in Fig. 2b–d
Source Data Fig. 4 Raw statistical data for the plots in Fig4a–c,e,f
Source Data Fig. 4 Uncropped images for 4d
Source Data Extended Data Fig. 5 Raw statistical data for the plots in Extended Data Fig. 5a,b
Source Data Extended Data Fig. 5 Uncropped images for Extended Data Fig. 5c,d
Source Data Extended Data Fig. 9 Raw statistical data for the plots in Extended Data Fig. 9a–e,g–i,k
Source Data Extended Data Fig. 9 Uncropped images for Extended Data Fig. 9l
