# Supplementary material for: CENP-N promotes the compaction of centromeric chromatin
Source: Nat Struct Mol Biol. 2022 Apr 14;29(4):403–13. doi: 10.1038/s41594-022-00758-y (PMC9010303; doi:10.1038/s41594-022-00758-y)
Supplement: Supplementary file 1 — Materials and Methods, Supplementary Note, legends for Extended Data figures and legends for Videos 1–3. [file 41594_2022_758_MOESM1_ESM.pdf]

nature portfolio

https://doi.org/10.1038/s41594-022-00758-y

Supplementary information

CENP-N promotes the compaction of centromeric chromatin

In the format provided by the authors and unedited

Supplementary Materials for

CENP-N promotes the compaction of centromeric chromatin

Keda Zhou[1], Magda Gebala[2], Dustin Woods[3], Kousik Sundararajan[2], Garrett Edwards[1], Daniel Krzizike, Jeff Wereszczynski[4], Aaron Straight[2,*], and Karolin Luger[1, 5, *]

Correspondence to: karolin.luger@colorado.edu, astraigh@stanford.edu

This PDF file includes:

Materials and Methods
Supplementary Note
Figs. S1 to S10
Captions for Movies S1 to S3

Other Supplementary Materials for this manuscript include the following:

Movies S1 to S3

Materials and Methods

Protein expression and purification

CENP-N$^{1-289}$ was cloned into pACEBac1 with a 6-his tag at its C-terminus. It was expressed in Sf21 insect cells (Invitrogen, Thermo Fisher Scientific, USA), and purified through affinity tag (nickel beads), followed by size-exclusion column (S200) as described (*13*). Full length CENP-N:CENPL was a gift from Andrea Musacchio (MPI Dortmund). Histones H3, H4, H2A and H2B were purchased from 'The Histone Source' at Colorado State University. $(H3\text{-}H4)_2$ and H2A-H2B were refolded as described [1]. H2B T112C was labeled by Alexa 488 or Atto N 647 during the refolding procedure as previously described [1]. CENP-A and H4 were co-expressed from a bi-cistronic plasmid in BL21(DE3)pLysS cells. Two-step purification over hydroxyapatite and Hitrap SP FF yielded pure, folded $(CENP\text{-}A{:}H4)_2$ [2].

Nucleosome reconstitution

Nucleosomes were reconstituted using the salt dialysis method as described [1]. DNA, H2A-H2B dimer and $(CENP\text{-}A\text{-}H4)_2$ or $(H3\text{-}H4)_2$ tetramer were mixed at molar ratios of 1.0:2.4:1.2 in reconstitution buffer A (Tris-HCl pH 7.5, 2 M NaCl, 1 mM DTT, and 1 mM EDTA) and dialyzed against buffer A. Using a peristaltic pump (Econo pump, Bio-Rad, Richmond, CA), buffer B (Tris-HCl pH 7.5, 50mM NaCl, 1 mM DTT, and 1 mM EDTA) was exchanged with buffer A at the rate of 1.5 ml/minute overnight (>16 hours). The nucleosomes were then dialyzed against buffer B for 2 hours. Nucleosome quality was monitored by native PAGE.

Analytical Ultracentrifugation (AUC)

Sedimentation Velocity AUC (SV-AUC) was performed with absorbance optics (λ = 260 nm) to evaluate the homogeneity, size and shape of nucleosome and its complex with CENP-N. The nucleosome concentration in SV-AUC was fixed at 250 nM (OD260~0.5). CENP-N was added at a ratio of 3:1 or higher, as indicated. The buffer contained 20 mM Tris-Cl (pH7.5), 1 mM EDTA, 1 mM DTT and NaCl at different concentration (50 mM or 200 mM). For mono-nucleosome samples, the centrifugation speed was 30-35,000 rpm (72,576-98,784 g) at 20°C in a Beckman XL-A ultracentrifuge (An60Ti rotor).

UltraScan III version 4.0 [3] was used for the data analysis. 2-dimensional Spectrum Analysis (2DSA; [4,5]) subtracted time-invariant and radial-invariant noise contributions from the experimental AUC data. Enhanced van Holde-Weischet Analysis method was then performed to obtain Integral sedimentation coefficient distributions (G(s)) from noise-corrected experimental data. The AUC figures were Enhanced van Holde-Weischet plots [6, 7].

Electrophoretic Mobility Shift Assay (EMSA)

5% native PAGE was used to monitor the nucleosome quality as well as binding events between CENP-N and nucleosomes [8]. The buffer for binding assays contained 20 mM Tris-Cl (pH7.5), 1 mM EDTA, 1 mM DTT and 50 mM NaCl.

Forster Resonance Energy Transfer (FRET)

CENP-A or H3 nucleosomes were reconstituted with Alexa 488 or Atto N 647 labeled H2B (T112C). Alexa 488 served as the donor while Atto N 647 as the acceptor. Equal amount (250 nm) of donor nucleosome and acceptor nucleosome were mixed in the absence or presence of CENP-N (molar ratio to CENP-A nucleosome at 2:1 and 4:1). FRET signal was obtained with the excitation wavelength at 482(+/-16) nm and emission wavelength at 675(+/-50) nm. The

FRET value were then corrected as described [9]. The signal from the emissions of donor and acceptor in FRET channel were removed from the raw FRET value.

CryoEM sample and grid preparation

CENP-N and CENP-A mono-nucleosome were mixed at a 5:1 ratio in buffer containing 20 mM Tris-Cl (pH7.5), 1 mM EDTA, 1 mM DTT and 50 mM NaCl. The complex was concentrated to 1-2 µM before grid preparation. For grid plunge freezing, 4 µl sample was loaded on a holy carbon grid (Quantifoil 1.2/1.3 Au) and flash frozen using a Vitrobot Mark IV (ThermoFisher Scientific). Blotting time was 4 s with >90% humidity.

CENP-N and CENP-A nucleosome arrays ('601' sequence, 167bpx12mer) were mixed at a 5:1 (nucleosome) ratio in buffer containing 20 mM Tris-Cl (pH7.5), 1 mM EDTA, 1 mM DTT and 300 mM NaCl. The buffer for the mixture was gradually exchanged/dialyzed against the same buffer containing 50 mM NaCl (overnight at 4°C). The sample was then concentrated to 0.1-0.2 mg/ml before grid preparation. For grid plunge freezing, 4 µl sample was loaded on a holy carbon grid (C-Flat 2/2 Au) and flash frozen using a Vitrobot Mark IV (Thermo Scientific). Blotting time was 4 s with >90% humidity.

The structure of a crosslinked CENP-A nucleosomal array ('601' sequence, 167x12mer) was an accidental product of an attempt to GraFix (gradient fixation by glutaraldehyde) CENP-N and CENP-A nucleosome array in buffer containing 20 mM HEPES (pH7.5), 1 mM EDTA, 1 mM DTT and 200 mM NaCl overnight, which caused CENP-N to dissociate. Fractions were combined to be quenched by dialyzing against the buffer containing 20 mM Tris-Cl (pH7.5), 1 mM EDTA, 1 mM DTT and 50 mM NaCl for >2 hours. The sample was concentrated to 0.1-0.2 mg/ml before grid preparation. For grid plunge freezing, 4 µl sample was loaded on a holey carbon grid (C-Flat 2/2 Au) and flash frozen using a Vitrobot Mark IV (Thermo Scientific). Blotting time was 4 s with >90% humidity.

CryoEM images acquisition and analysis

CENP-N in complex with CENP-A mono-nucleosome (α-satellite DNA) was imaged at nominal magnification of 29000x on a FEI Titan Krios (300 kV), equipped with a Gatan K3 direct detector. Pixel size was 0.8211 Å. The movies were captured in counting mode with electron dose rate at 10 electrons per pixel per second for 7.2 s and 0.2 s per frame. Defocus range was −1.0 to −2.5 µm. SerialEM was used for all cryoEM data collections [10]. The raw movies were motion corrected, followed with Constant transfer function (CTF) estimation in cryoSPARC2 [11]. Nucleosome like particles were manually picked to generate the templates for template picking. 555,254 particles were picked. The box size of particle extraction was set at 400 pixels. One round of 2D classification was performed to remove the low resolution or non-nucleosome particles. Ab initio classification was used to generate the initial maps (3 classes). One class (class 1) was mainly at mono-nucleosome level. The other two classes (class 2&3) were both di-nucleosome organization. Class1 was continually processed by 2D classification as well as heterogeneous refinement to remove low resolution particles. Non-uniform refinement [12] was performed to obtain the highest resolution map for class1. For class2 and class3, they looked very similar to each other. Therefore, the particles from these two classes were combined to be processed by 2D classification and heterogeneous refinement to remove low resolution or unaligned particles. The model was finally refined by non-uniform refinement [12]. 3DFSC was used to evaluate and reflect the directional resolution [13]. The map for complex in di-nucleosome

form showed very heterogeneous resolutions in different direction due to particle orientation problems.

CENP-N in complex with CENP-A mono-nucleosome (601 DNA) was imaged at nominal magnification of 29000x on a FEI Titan Krios (300 kV), equipped with a Gatan K2 Summit direct detector. Pixel size was 1.02 Å. The movies were captured in super resolution mode with electron dose rate at 10 electrons per pixel per second for 8 s and 0.2 s per frame. Defocus range was −1.0 to −2.5 µm. The raw movies were motion corrected by Motioncor2 [14], followed with Constant transfer function (CTF) estimation through GCTF [15]. To classify nucleosome pairs in the stack, the size of pixel box was enlarged to 256 which was enough to include two nucleosomes. 2D classifications were performed in cryoSPARC2.0 to select the 2D classes representing nucleosome stacks. Ab initio reconstruction was used to generate the initial model, followed by two rounds of heterogenous refinement (two classes). Both classes were refined by non-uniform refinement [12]. 3DFSC analysis was performed to evaluate the directional resolution[13].

Chromatin fiber with CENP-N was imaged at nominal magnification 22500x by a FEI Titan Krios (300 kV), equipped with a Gatan K2 Summit direct detector. The images were captured in super resolution mode (pixel size 0.655 Å) with electron dose rate at 10 electrons per pixel per second for 10 s. Defocus range was −1.3 to −2.5 µm. The raw movies were motion corrected, followed with Constant transfer function (CTF) estimation in cryoSPARC2 [11]. Cryolo was used for particle picking [16]. Extracted particles were imported into cryoSPARC2. 2D classifications were performed to remove the junk particles. Ab-Initio reconstruction (2 classes) was performed to generate the initial map and remove the junk particles. Non-uniform refinement was used to do the final refinement [11]. 3DFSC was used to evaluate the global resolution [13].

The crosslinked chromatin fiber was imaged at nominal magnification of 22,500x on a FEI Titan Krios (300 Kv), equipped with a Gatan K3 direct detector. The images were captured in counting mode (pixel size 1.064) with electron dose rate at 10 electrons per pixel per second for 7.8 s (65 frames with 0.12 s per frame). Defocus range was −0.9 to −2.1 µm. The raw movies were imported and processed by motion correction and CTF estimation in cryoSPARC2. Folded chromatin particles were manually picked to generate the templates. 2D classifications were performed to remove the junk particles. Ab-Initio reconstruction (2 classes) was performed to generate the initial map and remove the junk particles. Non-uniform refinement was used to do the final refinement [12].

Modeling of the structures

The structure of CENP-N in complex with a mono-nucleosome (α-satellite DNA) was generated by combining CENP-N and histones from pdb:6c0w [17] and the α-satellite DNA from pdb: 1kx5[18]. DNA was shifted manually by 1 bp as indicated in supplementary text. ISOLDE was performed to refine the model[19,20]. The structure model for CENP-N in complex with di-nucleosome was initially generated by docking the model of CENP-N in complex with mono-nucleosome twice in the map. Although the map shows high heterogeneity on the resolution in different direction, it did not affect unambiguous docking of the model.

The structure model of CENP-A chromatin fiber with CENP-N was generated by placing 6 copies of pdb:6c0w into the map.

Generation of cell lines

CRISPR-Cas9 based genome engineering was used to tag CENP-N with AID-sfGFP at the C-terminus as descripted previously [8]. Briefly, native CENP-N locus in osTir1 Flp-In TRex-DLD1 cell line was tagged with AID-sfGFP in the C-terminus. Transgenic CENP-N constructs (WT full length CENP-N, CENP-N K102A, CENP-N 7-ala mutants) with C-terminus with tandem mRuby2 and 3xFlag were induced using FRT/Flp-mediated recombination of pcDNA5/FRT/TO-vector and selected with 100 µg/ml Hygromycin B. WT full length CENP-N was expressed from ASP 3329 [8]. CENP-N 7-ala mutation in CENP-N (K102A K105A N106A K109A K110A Q113A R114A) was introduced in ASP 3329 using Gibson Assembly (CENP-N 7-ala Plasmid ASP 4242; forward Gibson oligo: AGCTGCCAGGATCGCTGCAAAGCTGGCCGCGAACT-GCGCCATGTCGAACAGATCCACGTCCTCG reverse Gibson oligo: GCGCAGTTCGCG-GCCAGCTTTGCAGCGATCCTGGCAGCTGCGCTGAAGAACGTTACCGTTTCTTTTCG) and confirmed by sequencing. CENP-N K102A mutation in CENP-N was introduced in ASP 3329 using Gibson Assembly (CENP-N K102A Plasmid ASP 4243; forward Gibson oligo: GGACGTGGATCTGTTCGACATGGCGCAGTTCAAGAACAGCTTTAAAAAG reverse Gibson oligo: CGAGGACGTGGATCTGTTCGACATGGCGCAGTTCAAGAACAGCTT-TAAAAAGATC) and confirmed by sequencing. Full plasmid sequences are available from the Stanford Digital Repository (https://purl.stanford.edu/gz478gq3828).

Cell culture

All cell lines were grown in RPMI-1640 medium supplemented with 10 % fetal bovine serum (FBS), 100 U/mL penicillin/0.1 mg/mL streptomycin, 2 g/L sodium bicarbonate, and 2 µg/mL puromycin. Cells were tested for mycoplasma contamination at the start of cell line generation by PCR and conditions were monitored by the absence of cytoplasmic DNA staining by microscopy. Degradation of the endogenous AID-tagged CENP-N was carried out by adding 1 mM 3-Indoleacetic acid (IAA) (Sigma, # I2886), followed by 1 h incubation. Expression of transgenic CENP-N mutants was induced using 20 ng/mL doxycycline (Sigma, #D9891) accompanied by degradation of the endogenous CENP-N using 1 mM IAA, followed by 24 h incubation. All drugs were added directly to the media.

Preparation of crosslinked chromatin lysates

Cells were grown to 80% confluence in 150-mm dishes (10 plates per chromatin batch, 70 – 80 · 106 cells). Cells were trypsinized, twice pelleted at 1000 g for 5 min and washed in PBS buffer. Crosslinking was carried out by addition of formaldehyde (16 % formaldehyde (w/v), methanol-free, Thermo Scientific, #28908) to 10 mL of PBS buffer for 1 % formaldehyde final concentration, rocking for 10 min at room temperature. The crosslinking was quenched by addition of glycine (125 mM final concentration), followed by rocking for 5 min at room temperature. Subsequently, cells were pelleted at 1000 g for 5 min, washed in PBS and pelleted again at the same speed. The final pellet was snap-frozen in liquid nitrogen and stored in -80 ºC.

All subsequent steps to enrich for nuclei were carried out on ice or in 4 ºC – cold room, following a protocol developed by Becker (*40*). Cell pellets were thawed and incubated for 10 min in 1 mL Hypotonic Lysis Buffer (20 mM HEPES-KOH pH 7.5, 20 mM KCl, 1 mM EDTA, 10 % glycerol, 1 % IGEPAL CA-630, 0.25 % Triton-X, 1 mM DTT, 0.2 mM PMSF, Protease Inhibitor Cocktail (cOmplete, EDTA-free, Roche #11873580001)) and disrupted with 50 strokes in a 7 mL Dounce homogenizer (Wheaton, # 357542). Nuclei were pelleted at 1,300 g for 5 min in a swinging bucket, benchtop centrifuge, washed with hypotonic buffer, and pelleted again at the same speed. Pellets were resuspended in 1 mL Nuclear Wash Buffer (10 mM Tris-HCl pH

8.0, 200 mM NaCl, 1 mM EDTA, 0.5 mM EGTA, 1 mM DTT, 0.2 mM PMSF, Protease Inhibitor Cocktail) and incubated for 10 min, rocking at 4 ºC. Nuclei were pelleted at 1,300 g for 5 min and resuspended in 1.2 mL Sonication Lysis Buffer (10 mM Tris-HCl pH 8.0, 100 mM NaCl, 1 mM EDTA, 0.5 mM EGTA, 0.5 % N-lauroylsarcosine, 0.1 % sodium deoxycholate, 1 mM DTT, 0.2 mM PMSF, Protease Inhibitor Cocktail) and aliquoted into 400-µL samples (180-240 µg of DNA per sample). Sonication was carried out in 1.5 mL microtubes (Axygen®, MCT-150-C-S) using a Diagenode Bioruptor Plus UCD-300 (power HI, 20 cycles of 30 s on / 30 s off), samples were immersed in 4 ºC water bath. After sonication, lysates were centrifuged at 15,000 g for 15 min at 4 ºC to pellet debris. Supernatants were combined and subsequently loaded on sucrose gradients in aliquots of 300-400 µl per gradient.

Sucrose gradient sedimentation of chromatin

5 %–40 % linear sucrose gradients were poured into 12-mL centrifuge tubes (Beckman Coulter, #344059, 14 · 89 mm), using a Biocomp Gradient Master™. Gradients were prepared by layering bottom-up 6 mL of 5 % and 6 mL 40 % sucrose solution directly in the centrifuge tubes, capped, placed on a rotary plate on the Gradient Master and gently mixed by rotation at a 81.5º angle and a speed of 21 rpm. Sucrose solutions were prepared in 10 mM Tris-HCl, pH 8.0, supplemented with 100 mM NaCl, 1 mM EDTA, 0.5 mM EGTA, 1 % Triton-X, 0.1 % N-lauroylsarcosine, 1 mM DTT, 0.2 mM PMSF, Protease Inhibitor Cocktail. Sucrose concentrations were determined using a benchtop refractometer (Milton Roy Abbe).

Crosslinked and sheared chromatin was mixed with the 40 % sucrose solution for a 5 % final sucrose concentration and gently layered (300-400 µL, 200-360 µg of DNA) on the 5% - 40% gradient. Gradients were centrifuged using a Beckman SW 41 Ti rotor at 36,000 rpm for 4 h (4 ºC), with slow acceleration and deceleration settings. Subsequently, gradients were fractionated top-down using a micropipet, generating 24 fractions of 500 µL. To remove sucrose, fractions were buffer exchanged 3-4 times into Nuclear Wash Buffer and concentrated (100 µL final volume) using 500-µL centrifugal filters with 30K cut-off (Amicon Ultra, UFC 503096). No protein or DNA loss was detected in the flow through by Bradford assay or UV-absorbance. Fractions were de-crosslinked by supplementing with SDS (1 % final concentration), transferring to PCR tubes and heating to 70 ºC for at least 16 h using Peltier Thermal Cycler PTC-200 (MJ Research).

Western blotting

Protein concentration in fractions was quantified by Bradford assay (Bio-Rad Laboratories, #5000006). Protein samples were mixed with 4x SDS sample loading buffer (200 mM Tris-HCl, 400 mM DTT, 8 % (w/v) SDS, 30 % (w/v) glycerol, bromophenol blue) and heated to 90 ºC for 10 min. Samples were loaded (equal concentration of 20-40 µg of total protein load per lane) in SDS-PAGE 4 %-20 % Tris-glycine continuous protein gels and run in SDS-containing Tris-glycine-chloride buffer (50 mM Tris, 380 mM glycine, 1 % SDS). Samples were transferred in CAPS transfer buffer (10 mM 3-[cyclohexylamino]-1-propanesulfonic acid, pH 11.3, 0.1% SDS, and 20% methanol) onto polyvinylidene fluoride membrane (PVDF) (Bio-Rad Laboratories, #1620177) for 4 h at 400 mA, 4 °C. After the transfer, PVDF membranes were washed in TBS-T buffer (20 mM Tris, pH 7.5, 150 mM NaCl, 0.1% Tween-20) and blocked in 5% nonfat diary milk in TBS-T buffer for 30 min at room temperature. Subsequently, membranes were incubated with primary antibodies overnight at 4 ºC. Primary antibodies were diluted in 5% milk/TBS-T at the following concentrations: rabbit anti-CENP-A (1 µg/mL, custom), rabbit anti-CENP-C (2

μg/mL) rabbit anti-H3K9me3 (2 μg/mL, Abcam, ab 8898), rabbit anti-H4-antibodies (2 μg/mL, Abcam, ab 7311) and mouse anti-Flag M2 antibodies (F1804; Sigma-Aldrich). HRP-conjugated secondary antibodies (Bio-Rad Laboratories, anti-rabbit #170-6515, anti-mouse #170-6516) were diluted 1:4,000 in 5% milk/TBS-T. Blots were developed using Super-Signal West Pico Plus Chemiluminescent Substrate (Thermo Fisher Scientific #34577) and Pierce ECL Western Blotting Substrate (Thermo Fisher Scientific #32106), visualized using a film processor SRX-101A (Konica Minolta Medical and Graphic), and analyzed with an image processor GelAnalyzer 19.1 (GelAnalyzer.com) and Origin2020 (OriginLab). Antibodies against human CENP-A and CENP-C were generated by immunization of rabbits with GST fusions to CENP-A (amino acids 1-42, ASP426) and CENP-C (amino acids 1-537) in pGEX-6P. To affinity purify the antiserum we cleaved the antigen from GST using Prescission protease and coupled each antigen to NHS-Agarose (Affigel-10, Bio-Rad). Antibodies were bound to the column and eluted with 0.15 M NaCl, 0.2 M Glycine-HCl pH 2.3 followed by neutralization with 1M Tris pH 8.0. Antibodies were then dialyzed against 50% Glycerol in HEPES pH7.7, 150 mM KCl and stored at -20°C. Antibodies were verified by western blotting and immunofluorescence in human cells and selective degradation of reactive signal after AID degron induced destruction of each protein [8].

Immunofluorescence

Cells were seeded on poly-L-lysine coated glass coverslips and allowed to attach for 24 hours. Cells were then treated with or without 1 mM IAA and with or without 200 ng/mL doxycycline for 24 hours. Coverslips were then washed with PBS + 1 mM MgCl2 and CaCl2, permeabilized with PBKCl (139.7 mM KCl, 11.8 mM KH2PO4) with 0.5% Triton X-100 for 5 minutes and fixed in PBKCl/0.5% Triton X-100/3.7% formaldehyde for 10 minutes and blocked for at least 30 minutes in antibody dilution buffer (20 mM Tris HCl, pH 7.4, 150 mM NaCl, 0.1% Triton X-100, 2% bovine serum albumin, and 0.1% sodium azide). Coverslips were then incubated for 1 hour with antibody dilution buffer containing the following primary antibodies – Transgenic CENP-N was detected using RFP antibody pre-adsorbed (α-mCherry) (Rockland Inc. Cat. No. 600-401-379) at 1 ug/ml. Centromeres were detected using α-centromere antibody derived from human CREST patient serum (Antibodies, Inc. Cat. No. 15-234-0001) at 1:100 dilution. Primary antibodies were detected using Alexa-568 or -647 conjugated goat secondary antibodies (Molecular Probes). Endogenous CENP-N was imaged directly using sfGFP fluorescence. Nuclei (DNA) were stained using 10 ug/ml Hoechst 33258.

Imaging was performed using a DeltaVision Core deconvolution microscope (Applied Precision) with a Sedat quad-pass filter set (Semrock) and monochromatic solid-state illuminators. Images were acquired using a CoolSnap HQ CCD camera (Photometrics) as Z-stacks with Z-sections of 0.2 um steps using a 60x, 1.4 NA Plan Apochromat oil immersion objective (Olympus). Image analyses were done using centromere finder [21], available at http://cjfuller.github.io/imageanalysistools/). Quantification of microscopy experiments involved three independent experiments with at least 100 cells per coverslip/condition per experiment. Representative images in figures were generated from maximum intensity projections of deconvolved Z-stacks using softWoRx 4.1.0 software (Applied Precision).

Clonogenic Survival Assay

Clonogenic survival assay was performed as described [8]. Briefly, cells were seeded with 1000 cells/well in 6-well plates and allowed to attach for 24 hours. Cells were treated with 0.1 ug/ml

doxycycline and/or 0.1 mM IAA before fixing and staining with Crystal Violet solution (0.25% crystal violet in 3.5% formaldehyde and 72% methanol) for 30 minutes. Cells were then destained in water and allowed to air dry before being imaged (using a scanner). Cell viability was quantified using total intensity of crystal violet stain in each well from scanned images processed using ImageJ.

In Silico System Construction

Using the 6EQT initial coordinate structure [17] and the nucleosome dimer cryo-EM map, we created models for use in molecular dynamics simulations to understand the influence of CENP-N on the nucleosome dimer. We modeled the missing loops and tails in each system by using Modeller via the Chimera graphical user interface [22,23]. This resulted in the histone tails extended outward from each nucleosome with unique intrinsically disordered conformations. Once structures were assembled, the coordinates were aligned to the cryo-EM map using the Molecular Dynamics Flexible Fitting (MDFF) method within the NAMD 2.13 engine in implicit solvent [24]. After 300 ps of biasing, the system reached an equilibrium and could not be refined further. The structure from this point was used to seed simulations of the dimer containing one of the following: 2 CENP-N, 1 CENP-N, or no CENP-N. The latter two systems were created by removing the CENP-N coordinates from the initial structure file.

Simulation Methods

All systems were prepared with tleap from the AmberTools18 software package [25]). Each system was solvated in a TIP3P water box extending at least 10 Å from the solute [26,27]. Using Joung-Cheatham ions, the solvent contained 150 mM NaCl, and sodium cations to neutralize negative charges [28]. The AMBER14SB and BSC1 force fields were used for protein and DNA interactions, respectively [29]. All simulations were performed locally using the Amber18 engine on GeForce GTX 1080 GPUs via the Pinot computing cluster [25].

Systems were minimized twice for 10,000 steps, first with a 10 kcal·mol-1·Å-2 harmonic restraint applied to the solute and then followed by no restraints. Using the Langevin thermostat and an electrostatic cutoff distance of 10 Å with long range interactions treated with particle mesh Ewald calculations [30], systems were then heated in the NVT ensemble from a temperature of 5 K to 300 K over 5 ps with a 10 kcal/mol kcal·mol-1·Å-2 solute restraint [30]. This restraint was then reduced from 10 kcal/mol to 0 kcal/mol over 600 picoseconds in the NPT ensemble, followed by a 1 ns equilibration period to assess the system stability. Production runs with no restraints and a temperature of 300 K were also performed in the NPT ensemble using the Berendsen barostat. To allow for the extended timestep of 4 femtoseconds, we used Hydrogen Mass Repartitioning (HMR) which increases the mass of hydrogens while equally decreasing the parent atom masses to moderate the vibrations of high-frequency bonds [31-33]. Simulations required 100 ns of equilibration time to allow the tails to relax down onto the DNA of their respective nucleosomes. To boost the speed and overall efficiency, after equilibration, the solvent was removed from each system and then resolvated. Since the tails had collapsed, the overall dimensions of the solute were reduced translating to less water required upon solvation, resulting in a fewer number of particles and a boost to the overall simulation speed. Simulations of each system were performed in triplicate for 600 ns each, totaling in 5.40 µs. Allotting for the 100 ns mentioned above, we have amassed 4.50 µs of post-equilibration data.

Movies of the stacked-nucleosome simulations were created by using a combination of Visual Molecular Dynamics (VMD; [34] and FFmpeg [35]. VMD was used to generate the individual frames

which employed Tachyon ray tracing [36] to implement advanced rendering features such as ambient occlusion lighting. The frames were merged to produce the final video by using FFmpeg, a free and open-source command-line based video processing software [35].

Contact Analysis

Analysis was conducted using AmberTools18 from the AMBER18 software package [25]. Protein-DNA contacts were defined between the heavy atoms of residues within 4.0 Å of one another. The statistical inefficiency was calculated for each residue to determine the number of statically independent data points throughout each simulation.

Supplementary Note

2.68 Å structure of CENP-A α satellite nucleosome in complex with CENP-N^{NT}

The structures of CENP-N^{NT} in complex with CENP-A nucleosome containing the 601 nucleosome positioning sequence were reported by multiple labs including our own [17,37,38]. The structure of a complex consisting of CENP-N^{NT}, CENP-C^{CC} and CENP-A nucleosomes reconstituted on α satellite DNA was also solved [39]. In these structures, the resolution for the CENP-A nucleosome ranged from 3.5-4.0 Å, while CENP-N^{NT} was at 4.0-4.5 Å resolution, and the side chains of CENP-N were not discernable in the density. In our new map, the resolution of CENP-N was better than 3.5 Å (Fig S1C), revealing density for key CENP-N amino acid side chains that are involved in the interaction with CENP-A (Extended data fig.10B), and largely confirming the accuracy of our previous structure (6C0W).

The resolution of the nucleosome (reconstituted with a 147 bp palindromic DNA fragment derived from α-satellite DNA, as described in [18], pdb 1kx5) in our new map is ~2.8-3.0 Å (fig S1C, D), and reveals density for individual base-pairs of nucleosomal DNA. This allowed us to precisely position each nucleotide for nearly the entire length of the DNA (Extended data fig.10D), which was not possible before. When attempting to fit the DNA derived from 1kx5 into the map, we observed a staggered 1 bp 'stretching' of DNA density around SHL ±2 compared to the input model from 1KX5 (bp 12-20; central 'dyad' base pair designated as 0, fig Extended data fig.10E). That is, the crystal structure has one extra base pair in each strand (staggered by 5 bp) for which there is no cryoEM density. This suggests that the 'natural' length of DNA organized by α-satellite DNA (and likely also by other DNA) is 145 bp rather than 147 bp as suggested by the crystal structures. The compression of DNA observed in the crystal structure compared to the cryoEM structure is caused by the end-to-end stacking of DNA in the crystal lattice [40]. This further suggests that the 'stretching' of DNA required for crystal lattice formation with 145 bp DNA [41] is the 'relaxed', natural in-solution state for most nucleosomal DNA sequences. Of note, the resolution of our earlier structure did not allow us to address this issue with 601 DNA in 6C0W. Our maps further confirm SHL ±2 as a 'soft-spot' to absorb DNA length variability [42].

Extended data figure 1. CryoEM analysis of CENP-N in complex with CENP-A mono-nucleosomes (reconstituted with 147 bp palindromic α satellite DNA). **A)** Raw cryoEM micrograph. Stacks are indicated by red lines. Size bar is 50 nm. **B)** 2D classifications of particles at the mono-nucleosome level. **C)** Local resolution of the 3D map for CENP-N in complex with CENP-A mono-nucleosomes. The color key indicates the resolution. **D)** 3DFSC analysis of reconstructed 3D map.

Extended data figure 2. CryoEM analysis of CENP-N mediated stacks of CENP-A mono-nucleosomes reconstituted with palindromic α-satellite DNA. **A)** Flow-chart of the analysis of di-nucleosomes in the stacks. **B)** 2D classification of di-nucleosomes with CENP-N. **C)** Local resolution of the 3D map for stacked CENP-A nucleosomes in complex with CENP-N. **D)** 3DFSC analysis of the reconstructed 3D map. The plot reveals significant orientation bias in the map reconstruction. Therefore, the estimated global resolution of 3.54 Å does not represent the overall map quality. In some orientations, the resolution is closer to 10 Å.

Extended data figure 3. CryoEM analysis of CENP-N mediated stacks of CENP-A mono-nucleosomes reconstituted with 601 DNA (*21*). **A)** Representative cryoEM micrograph. Stacks are highlighted by red lines. Size bar is 50 nm**. B)** 2D classification of the di-nucleosome with CENP-N in the stacks. **C)** Flow-chart on the analysis of di-nucleosomes in the stacks. **D)** Comparison of di-nucleosome maps derived for α satellite and 601 CENP-A nucleosomes.

Extended data figure 4. MD simulations of stacked nucleosomes with 0, 1, or 2 CENP-N. **A)** Diagram of the points used to construct stacked-nucleosome sampling graphs as depicted in Figure 1C, in addition to nucleosome parameters calculated for stacked-nucleosome simulations. Nucleosomes (blue) are shown in face-on (left) and profile (right) viewpoints, with DNA represented in a darker blue. Dyad points and their opposing points are represented as small red and green circles, respectively. CENP-N is shown in purple to provide a point-of-reference. **B)** From left to right, six histograms of stacking parameters for di-nucleosome systems: Shift, Slide, Rise (top), and Tilt, Roll, Twist (bottom), in analogy to the parameters used to describe the geometry of the DNA double helix. Histograms do not contain the first 100 ns of simulation time which was allotted for each system to achieve equilibration.

Extended data figure 5. Solution assays for CENP-N induced CENP-A nucleosome stacking. **A)** FRET analysis of homotypic CENP-A mono-nucleosome (CA) or H3 mono-nucleosome (H3) interactions in the absence or presence of CENP-N. Donor is a mono-nucleosomes containing Alexa 488 labeled H2B; Acceptor is a mono-nucleosome containing Atto N 647 labeled H2B. 250 nM donor and acceptor nucleosome; FRET intensity in dependence of [CENP-N]. Final concentration for NaCl is around 100 mM. Error bars from three independent measurements. Data are presented as mean values +/- SD. **B)** Salt concentration affects nucleosome stack formation. AUC analysis (van Holde-Weischet plots) of CENP-N in complex with CENP-A mono-nucleosomes at 60 and 200 mM NaCl). CN: CENP-N$^{1-289}$ in complex with CENP-A mono-nucleosome. CA_MN: CENP-A mono-nucleosome. **C)** CENP-N mutant (K102A) binds to CENP-A nucleosomes as well as wild-type CENP-N (5% native PAGE). 250 nM CENP-A nucleosome was combined with CENP-N at ratios of 2:1, 4:1 and 8:1 in buffer containing 50

mM NaCl, 20 mM Tris-HCl (pH 7.8), 1 mM EDTA, 1 mM DTT. **D)** deletion of the H4 N-terminal tail (Δ19) does not affect the specific interaction between CENP-N and CENP-A nucleosomes. CENP-N was mixed with CENP-A nucleosome containing full length H4 or (Δ19) H4 at a 2:1 ratio in the same buffer as in A).

Extended data figure 6. Residue contacts of the CENP-N α6-helix with DNA of the DNA-directed nucleosome from simulations containing one (A) and two CENP-N (B). CENP-N 1 and CENP-N 2 are distinguished by the binding orientation of the α6-helix with the DNA grooves. CENP-N 1 (blue) binds directly into the DNA minor groove while CENP-2 (red) does not. Protein-DNA contacts were defined between the heavy atoms of residues within 4.0 Å of one another. . Standard errors were derived using n=15, where n is the number of statistically independent data points in each window as was determined by calculating the statistical inefficiency.

Extended data figure 7. CryoEM analysis of 12mer nucleosomal arrays in presence of CENP-N. **A)** raw cryoEM image of CENP-N in complex with 12-207mer 601 array. Size bar is 50 nm. **B)** raw cryoEM image of CENP-N in complex with 12-167mer array. Size bar is 50 nm. **C)** 2D classification of CENP-N in complex with 12-167mer array. **D)** Local resolution map of chromatin fiber. Color key indicates the resolution. **E)** 3DFSC analysis of cryoEM electron map

Extended data figure 8. CENP-N affects the folding of chromatin arrays. **A)** Comparison between tetra-nucleosomes in a chromatin fiber with CENP-N, and of a canonical tetra-nucleosome. Analysis of the relative orientation of the nucleosomes, in analogy to DNA base pair analysis, is shown in the middle panel, with numbers for the CENP-N array in red, and tetranucleosome array in black. **B)** CryoEM analysis of crosslinked chromatin arrays without CENP-N, raw cryoEM image. Size bar is 100 nm **C)** 2D classification of CENP-N in complex with 12-167mer array. **D)** Low resolution 3D cryoEM map illustrates ladder-like arrangement of nucleosomes.

Extended data figure 9. Western blot detection of centromeric proteins across 5-40% sucrose gradients and clonogenic survival assay. A) Comparison of CENP-A (gray) and H3K9me3 (lilac) distribution in the presence of endogenous WT-CENP-N. **B)** CENP-A distribution in the presence of endogenous (gray) and transgenic (blue) WT-CENP-N. **C)** Comparison of CENP-A (blue) and CENP-N (orange) in the presence of WT CENP-N. **D)** Comparison of CENP-A (blue) and CENP-N (yellow) in the presence of K102A CENP-N. **E).** Comparison of CENP-A (pink) and CENP-N (green) distribution in the presence of 7ala CENP-N. **F)** Comparison of CENP-A (gray) and CENP-C (red) distribution in the presence of endogenous WT-CENP-N. **G)** CENP-N (orange) and CENP-C (red) distribution in the presence of endogenous WT-CENP-N **H**) CENP-N (yellow) and CENP-C (green) distribution in the presence of K102A CENP-N. **I**) CENP-N (yellow) and CENP-C (green) distribution in the presence of 7ala CENP-N. **J)** Representative crystal violet-stained colonies from clonogenic survival assay showing viable CENP-N AID cells with the indicated transgenic CENP-N variant treated with or without 0.1 mM IAA and doxycycline for 14 days. After treatment of 1000 seeded cells/well, surviving colonies were fixed and stained with crystal violet stain. **K)** Quantification of average percentage survival (average crystal violet stain intensity) of CENP-N AID cells (except CENP-N 7-ala) from 3 biological replicates normalized to untreated cells.

(CENP-N 7-ala survival data is from single replicate). Data are presented as mean values +/- SD. **L)** Western blot of CENP-A and H4 histone distribution across 5-40 % sucrose gradient. Presence of CENP-A signal indicates fractions containing the centromeric chromatin whereas H4 signal represents the overall amount of chromatin loaded onto a SDS gel.

Extended data figure 10. The structure of CENP-A α satellite nucleosome in complex with CENP-N^{NT}. A) 2.65 Å cryoEM map of CENP-A α satellite nucleosome in complex with CENP-N^{NT}. The components are colored as indicated. **B) D**ensity (with model) of the interface between CENP-N and histones. **C)** Density (with model) of the interface between CENP-N and nearby DNA. **D)** The model to density of DNA. **E)** α satellite DNA is compressed by one base pair in the crystal structure of the nucleosome (pdb 1KX5), compared to our cryoEM structure where DNA ends are unconstrained.

Movie S1. Simulation of the stacked-nucleosomes bound to two CENP-N. Shown is the DNA (white), CENP-N (purple), histone H3 (blue), histone H4 (green), histone H2A (yellow), and histone H2B (red). The simulation time evolution is presented in the bottom right-hand corner of the video.

Movie S2. Simulation of the stacked-nucleosomes bound to one CENP-N. Shown is the DNA (white), CENP-N (purple), histone H3 (blue), histone H4 (green), histone H2A (yellow), and histone H2B (red). The simulation time evolution is presented in the bottom right-hand corner of the video.

Movie S3. Simulation of the stacked-nucleosomes not bound to any CENP-N. Shown is the DNA (white), histone H3 (blue), histone H4 (green), histone H2A (yellow), and histone H2B (red). The simulation time evolution is presented in the bottom right-hand corner of the video.

1 Muthurajan, U. M. *et al.* In Vitro Chromatin Assembly: Strategies and Quality Control. *Methods Enzymol* **573**, 3-41, doi:10.1016/bs.mie.2016.01.002 (2016).
2 Guse, A., Fuller, C. J. & Straight, A. F. A cell-free system for functional centromere and kinetochore assembly. *Nat Protoc* **7**, 1847-1869, doi:10.1038/nprot.2012.112 (2012).
3 Demeler, B. & G., G. in *Analytical Ultracentrifugation: Instrumentation, Software, and Applications.* (eds S. Uchiyama, W. F. Stafford, & T. Laue) 119-143 (Springer, 2016).
4 Brookes, E., Cao, W. & Demeler, B. A two-dimensional spectrum analysis for sedimentation velocity experiments of mixtures with heterogeneity in molecular weight and shape. *Eur Biophys J* **39**, 405-414, doi:10.1007/s00249-009-0413-5 (2010).
5 Brookes, E., Demeler, B. & Rocco, M. Developments in the US-SOMO bead modeling suite: new features in the direct residue-to-bead method, improved grid routines, and influence of accessible surface area screening. *Macromol Biosci* **10**, 746-753, doi:10.1002/mabi.200900474 (2010).
6 Edwards, G. B., Muthurajan, U. M., Bowerman, S. & Luger, K. Analytical Ultracentrifugation (AUC): An Overview of the Application of Fluorescence and Absorbance AUC to the Study of Biological Macromolecules. *Curr Protoc Mol Biol* **133**, e131, doi:10.1002/cpmb.131 (2020).

7 Demeler, B. & van Holde, K. E. Sedimentation velocity analysis of highly heterogeneous systems. *Anal Biochem* **335**, 279-288 (2004).
8 Cao, S., Zhou, K., Zhang, Z., Luger, K. & Straight, A. F. Constitutive centromere-associated network contacts confer differential stability on CENP-A nucleosomes in vitro and in the cell. *Mol Biol Cell* **29**, 751-762, doi:10.1091/mbc.E17-10-0596 (2018).
9 Hieb, A. R., D'Arcy, S., Kramer, M. A., White, A. E. & Luger, K. Fluorescence strategies for high-throughput quantification of protein interactions. *Nucleic Acids Res* **40**, e33, doi:gkr1045 [pii]10.1093/nar/gkr1045 (2012).
10 Mastronarde, D. N. Automated electron microscope tomography using robust prediction of specimen movements. *J Struct Biol* **152**, 36-51, doi:10.1016/j.jsb.2005.07.007 (2005).
11 Punjani, A., Rubinstein, J. L., Fleet, D. J. & Brubaker, M. A. cryoSPARC: algorithms for rapid unsupervised cryo-EM structure determination. *Nat Methods* **14**, 290-296, doi:10.1038/nmeth.4169 (2017).
12 Punjani, A., Zhang, H. & Fleet, D. J. Non-uniform refinement: adaptive regularization improves single-particle cryo-EM reconstruction. *Nat Methods* **17**, 1214-1221, doi:10.1038/s41592-020-00990-8 (2020).
13 Tan, Y. Z. *et al.* Addressing preferred specimen orientation in single-particle cryo-EM through tilting. *Nat Methods* **14**, 793-796, doi:10.1038/nmeth.4347 (2017).
14 Zheng, S. Q. *et al.* MotionCor2: anisotropic correction of beam-induced motion for improved cryo-electron microscopy. *Nat Methods* **14**, 331-332, doi:10.1038/nmeth.4193 (2017).
15 Zhang, K. Gctf: Real-time CTF determination and correction. *J Struct Biol* **193**, 1-12, doi:10.1016/j.jsb.2015.11.003 (2016).
16 Wagner, T. *et al.* SPHIRE-crYOLO is a fast and accurate fully automated particle picker for cryo-EM. *Commun Biol* **2**, 218, doi:10.1038/s42003-019-0437-z (2019).
17 Pentakota, S. *et al.* Decoding the centromeric nucleosome through CENP-N. *elife* **6:e33442** (2017).
18 Davey, C. A. & Richmond, T. J. DNA-dependent divalent cation binding in the nucleosome core particle. *Proc Natl Acad Sci U S A* **99**, 11169-11174. (2002).
19 Croll, T. I. ISOLDE: a physically realistic environment for model building into low-resolution electron-density maps. *Acta Crystallogr D Struct Biol* **74**, 519-530, doi:10.1107/S2059798318002425 (2018).
20 Trabuco, L. G., Villa, E., Schreiner, E., Harrison, C. B. & Schulten, K. Molecular dynamics flexible fitting: a practical guide to combine cryo-electron microscopy and X-ray crystallography. *Methods* **49**, 174-180, doi:10.1016/j.ymeth.2009.04.005 (2009).
21 Moree, B., Meyer, C. B., Fuller, C. J. & Straight, A. F. CENP-C recruits M18BP1 to centromeres to promote CENP-A chromatin assembly. *J Cell Biol* **194**, 855-871, doi:jcb.201106079 [pii]10.1083/jcb.201106079 (2011).
22 Sali, A. & Blundell, T. L. Comparative protein modelling by satisfaction of spatial restraints. *J Mol Biol* **234**, 779-815, doi:10.1006/jmbi.1993.1626 (1993).
23 Pettersen, E. F. *et al.* UCSF Chimera--a visualization system for exploratory research and analysis. *J Comput Chem* **25**, 1605-1612, doi:10.1002/jcc.20084 (2004).
24 Phillips, J. C. *et al.* Scalable molecular dynamics on CPU and GPU architectures with NAMD. *J. Chem. Phys.* **153** (2020).
25 Case, D. A. *et al.* AMBER 2018. *University of California, San Francisco, 2018* (2018).

26 Jorgensen, W. L., Chandrasekhar, J. & Madura, J. D. Comparison of simple potential functions for simulating liquid water. *J. Chem. Phys.* **79**, 926 (1983).
27 Mahoney, M. W. & Jorgensen, S. L. A five-site model for liquid water and the reproduction of the density anomaly by rigid, nonpolarizable potential functions. *J. Chem. Phys.* **112**, 8910 (2000).
28 Joung, I. S. & Cheatham, T. E., 3rd. Determination of alkali and halide monovalent ion parameters for use in explicitly solvated biomolecular simulations. *J Phys Chem B* **112**, 9020-9041, doi:10.1021/jp8001614 (2008).
29 Maier, J. A. *et al.* ff14SB: Improving the Accuracy of Protein Side Chain and Backbone Parameters from ff99SB. *J Chem Theory Comput* **11**, 3696-3713, doi:10.1021/acs.jctc.5b00255 (2015).
30 Hoover, W. G. Canonical dynamics: Equilibrium phase-space distributions. *Phys Rev A Gen Phys* **31**, 1695-1697, doi:10.1103/physreva.31.1695 (1985).
31 Feenstra, K. A., Hess, B. & Berendsen, H. J. C. Improving efficiency of large time-scale molecular dynamics simulations of hydrogen-rich systems. *Journal of computational chemistry* **20**, 786-798 (1999).
32 Hopkins, C. W., Le Grand, S., Walker, R. C. & Roitberg, A. E. Long-Time-Step Molecular Dynamics through Hydrogen Mass Repartitioning. *J Chem Theory Comput* **11**, 1864-1874, doi:10.1021/ct5010406 (2015).
33 Loubet, B., Kopec, W. & Khandelia, H. Accelerating All-Atom MD Simulations of Lipids Using a Modified Virtual-Sites Technique. *J Chem Theory Comput* **10**, 5690-5695, doi:10.1021/ct500100f (2014).
34 Humphrey, W., Dalke, A. & Schulten, K. VMD: visual molecular dynamics. *J Mol Graph* **14**, 33-38, 27-38, doi:0263785596000185 [pii] (1996).
35 Tomar, S. Converting video formats with FFmpeg. *Linux Journal* **10** (2006).
36 Stone, J. An Efficient Library for Parallel Ray Tracing and Animation. *Masters thesis* **Computer Science Department, University of Missouri-Rolla** (1998).
37 Chittori, S., Hong, J., Bai, Y. & Subramaniam, S. Structure of the primed state of the ATPase domain of chromatin remodeling factor ISWI bound to the nucleosome. *Nucleic Acids Res* **47**, 9400-9409, doi:10.1093/nar/gkz670 (2019).
38 Tian, T. *et al.* Molecular basis for CENP-N recognition of CENP-A nucleosome on the human kinetochore. *Cell Res* **28**, 374-378, doi:10.1038/cr.2018.13 (2018).
39 Allu, P. K. *et al.* Structure of the Human Core Centromeric Nucleosome Complex. *Curr Biol* **29**, 2625-2639 e2625, doi:10.1016/j.cub.2019.06.062 (2019).
40 Suto, R. K. *et al.* Crystal Structures of Nucleosome Core Particles in Complex with Minor Groove DNA-binding Ligands. *J Mol Biol* **326**, 371-380. (2003).
41 Vasudevan, D., Chua, E. Y. D. & Davey, C. A. Crystal structures of nucleosome core particles containing the '601' strong positioning sequence. *J Mol Biol* **403**, 1-10, doi:10.1016/j.jmb.2010.08.039 (2010).
42 Edayathumangalam, R. S., Weyermann, P., Dervan, P. B., Gottesfeld, J. M. & Luger, K. Nucleosomes in Solution Exist as a Mixture of Twist-defect States. *J Mol Biol* **345**, 103-114 (2005).
