# Supplementary material for: CENP-N promotes the compaction of centromeric chromatin
Source: Nat Struct Mol Biol. 2022 Apr 14;29(4):403–13. doi: 10.1038/s41594-022-00758-y (PMC9010303; doi:10.1038/s41594-022-00758-y)
Supplement: Supplementary file 2 — Reporting Summary [file 41594_2022_758_MOESM2_ESM.pdf]

nature portfolio

Corresponding author(s): Karolin Luger
Aaron Straight

Last updated by author(s): 2022/02/26

Reporting Summary

Nature Portfolio wishes to improve the reproducibility of the work that we publish. This form provides structure for consistency and transparency in reporting. For further information on Nature Portfolio policies, see our Editorial Policies and the Editorial Policy Checklist.

Statistics

For all statistical analyses, confirm that the following items are present in the figure legend, table legend, main text, or Methods section.

n/a	Confirmed	
☐	☒	The exact sample size (*n*) for each experimental group/condition, given as a discrete number and unit of measurement
☐	☒	A statement on whether measurements were taken from distinct samples or whether the same sample was measured repeatedly
☒	☐	The statistical test(s) used AND whether they are one- or two-sided<br>*Only common tests should be described solely by name; describe more complex techniques in the Methods section.*
☒	☐	A description of all covariates tested
☒	☐	A description of any assumptions or corrections, such as tests of normality and adjustment for multiple comparisons
☐	☒	A full description of the statistical parameters including central tendency (e.g. means) or other basic estimates (e.g. regression coefficient) AND variation (e.g. standard deviation) or associated estimates of uncertainty (e.g. confidence intervals)
☒	☐	For null hypothesis testing, the test statistic (e.g. *F*, *t*, *r*) with confidence intervals, effect sizes, degrees of freedom and *P* value noted<br>*Give P values as exact values whenever suitable.*
☒	☐	For Bayesian analysis, information on the choice of priors and Markov chain Monte Carlo settings
☒	☐	For hierarchical and complex designs, identification of the appropriate level for tests and full reporting of outcomes
☒	☐	Estimates of effect sizes (e.g. Cohen's *d*, Pearson's *r*), indicating how they were calculated

Our web collection on statistics for biologists contains articles on many of the points above.

Software and code

Policy information about availability of computer code

Data collection	cryoEM data was collected by SerialEM software. Florescence polarization assay data was collected by CLARIOstar.
Data analysis	CryoEM data analysis was performed by cryoSPARC 2.0,UCSF Chimera, PHENIX and Coot.<br>AUC data was analyzed by UltraScan III version 4.0.<br>Image analyses were performed using "centromere finder" software described in Moree et al 2011, available at http://cjfuller.github.io/imageanalysistools/<br>Western blotting: data analyzed with an image processor GelAnalyzer 19.1 (GelAnalyzer.com) and Origin2020 (OriginLab).

For manuscripts utilizing custom algorithms or software that are central to the research but not yet described in published literature, software must be made available to editors and reviewers. We strongly encourage code deposition in a community repository (e.g. GitHub). See the Nature Portfolio guidelines for submitting code & software for further information.

Data

Policy information about availability of data

All manuscripts must include a data availability statement. This statement should provide the following information, where applicable:

- Accession codes, unique identifiers, or web links for publicly available datasets
- A description of any restrictions on data availability
- For clinical datasets or third party data, please ensure that the statement adheres to our policy

Provide your data availability statement here.

Field-specific reporting

Please select the one below that is the best fit for your research. If you are not sure, read the appropriate sections before making your selection.

☒ Life sciences ☐ Behavioural & social sciences ☐ Ecological, evolutionary & environmental sciences

For a reference copy of the document with all sections, see nature.com/documents/nr-reporting-summary-flat.pdf

Life sciences study design

All studies must disclose on these points even when the disclosure is negative.

Sample size	In the AUC and FP assays, the concentrations for substrates and titrated proteins were determined based on the initial estimation of binding affinity from preliminary data.<br>For imaging: three biological replicates with > 75 cells in each replicate<br>Sample size for imaging was determined based on standardization experiments and published examples for observing differences in centromere localization of proteins. Sample size of at least 75 cells sufficiently covers cells in different stages of cell cycle.
Data exclusions	Low quality data from cryoEM was excluded in cryoSPARC2.0 to reach high resolution with criteria established in cryoSPARC.<br>For imaging: No data were excluded<br>For western blotting: no data were excluded
Replication	FP: two biological replicates with 2 technical replicates each.<br>For imaging: three biological replicates<br>For cell viability assay: 3 biological replicates with 2 technical replicates each<br>Western blotting: 2 biological replicates with 2 technical replication each<br>All attempts to replicate were successful; results and trends were consistent between replicates.
Randomization	For imaging and cell viability assay: All samples were treated similarly, and each replicate contained all conditions being compared. Sample allocation was randomized at the beginning of each experiment. For any given replicate, cells for each condition were sourced from the same parent population of cells.
Blinding	For imaging: Data acquisition and analyses were performed using identical parameters across all biological conditions being compared. Group allocation is not applicable for the treatment of cells from the same general population. During data collection and analyses, investigators were blinded to the sample conditions.

Reporting for specific materials, systems and methods

We require information from authors about some types of materials, experimental systems and methods used in many studies. Here, indicate whether each material, system or method listed is relevant to your study. If you are not sure if a list item applies to your research, read the appropriate section before selecting a response.

Materials & experimental systems

n/a	Involved in the study	
☐	☒	Antibodies
☐	☒	Eukaryotic cell lines
☒	☐	Palaeontology and archaeology
☒	☐	Animals and other organisms
☒	☐	Human research participants
☒	☐	Clinical data
☒	☐	Dual use research of concern

Methods

n/a	Involved in the study	
☒	☐	ChIP-seq
☒	☐	Flow cytometry
☒	☐	MRI-based neuroimaging

Antibodies

Antibodies used	For imaging: RFP antibody - Rockland Inc. Cat. No. 600-401-379, human CREST patient serum - Antibodies, Inc. Cat. No. 15-234-0001<br>Western blotting: rabbit anti-CENP-A (1 µg/mL, custom), rabbit anti-CENP-C (2 µg/mL, custom), rabbit anti-H3K9me3 (2 µg/mL, Abcam, ab 8898), rabbit anti-H4-antibodies (2 µg/mL, Abcam, ab 7311) and mouse anti-Flag M2 antibodies (F1804; Sigma-Aldrich). HRP-conjugated secondary antibodies (Bio-Rad Laboratories, anti-rabbit #170-6515, anti-mouse #170-6516)Alexa Fluor 568 conjugated goat anti-rabbit (1 ug/ml, A11036; Invitrogen), Alexa Fluor 647 conjugated goat anti-human (1 ug/ml, A21445; Invitrogen)
Validation	RFP antibody - validated by manufacturer (https://rockland-inc.com/store/Antibodies-to-GFP-and-Antibodies-to-RFP-600-401-379-O4L_24299.aspx), used in many previous publications including "Control of feeding by Piezo-mediated gut mechanosensation in Drosophila.;2021;ELife;Min S et al.";.<br>CREST patient serum - Localization in imaging as expected for centromere, used in many previous publications including "Mechanism

of centromere recruitment of the CENP-A chaperone HJURP and its implications for centromere licensing; 2019; Nature Communications; Pan D et al.;"

Eukaryotic cell lines

Policy information about cell lines

Cell line source(s)	DLD-1 OsTIR1 FlpIn cells were a gift from the Don W. Cleveland Lab, UCSD. SF21 was purchased from INVITROGEN.
Authentication	STR profiling has not been done.
Mycoplasma contamination	Cell lines were tested against Mycoplasma using PCR Mycoplasma Test Kit (PromoCell). No Mycoplasma was detected
Commonly misidentified lines (See ICLAC register)	no commonly misidentified lines was used in this study.
