# Supplementary material for: CENP-N promotes the compaction of centromeric chromatin
Source: Nat Struct Mol Biol. 2022 Apr 14;29(4):403–13. doi: 10.1038/s41594-022-00758-y (PMC9010303; doi:10.1038/s41594-022-00758-y)

Fig4D_1_NoAddback_Untreated_Ch1_Hoechst.jpg (1/1)

960 x 960

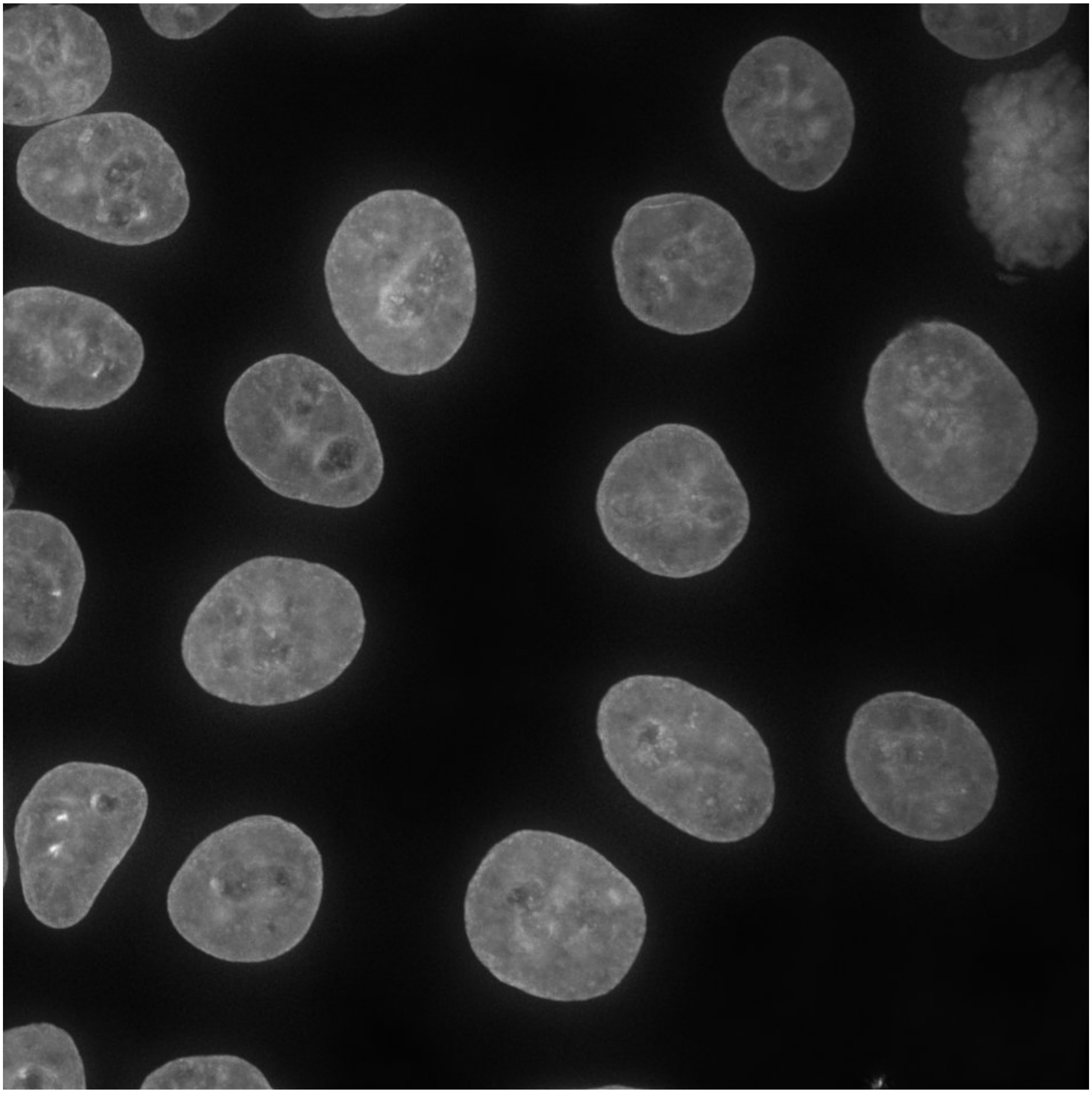

Fig4D_1_NoAddback_Untreated_Ch2_CREST.jpg (1/1)

960 x 960

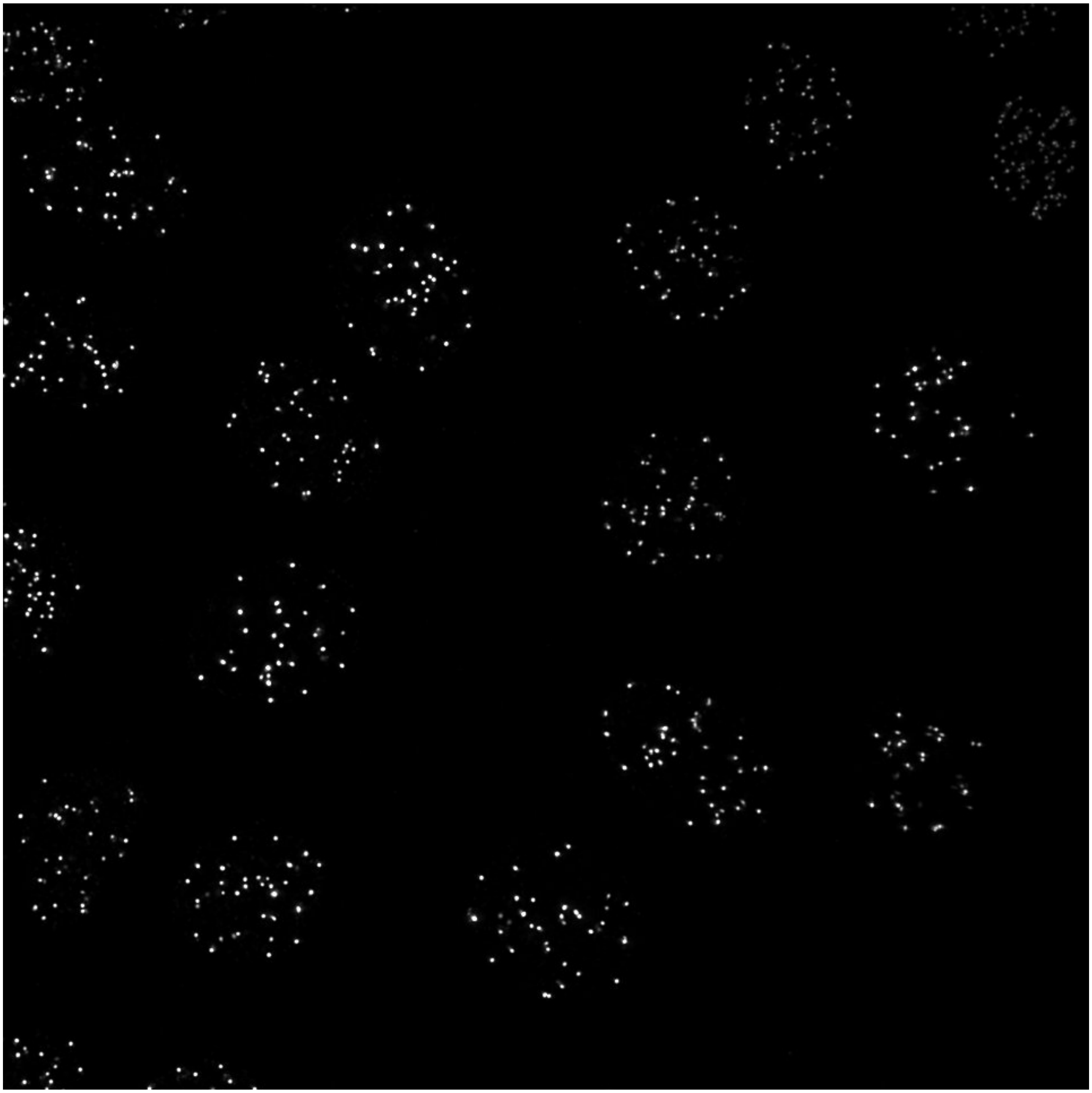

Fig4D_1_NoAddback_Untreated_Ch3_GFP.jpg (1/1)

960 x 960

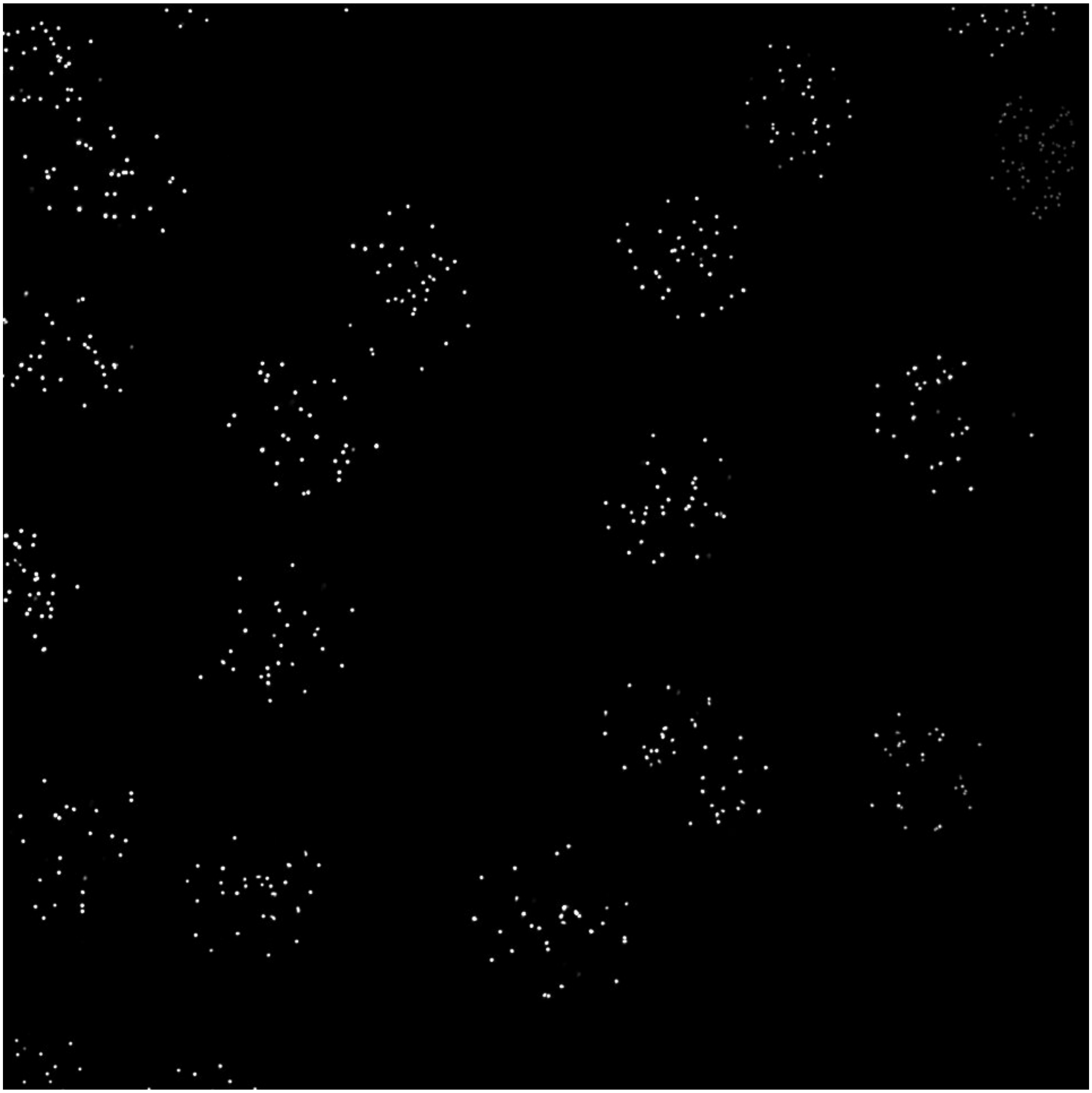

Fig4D_1_NoAddback_Untreated_Ch4_mRuby.jpg (1/1)

960 x 960

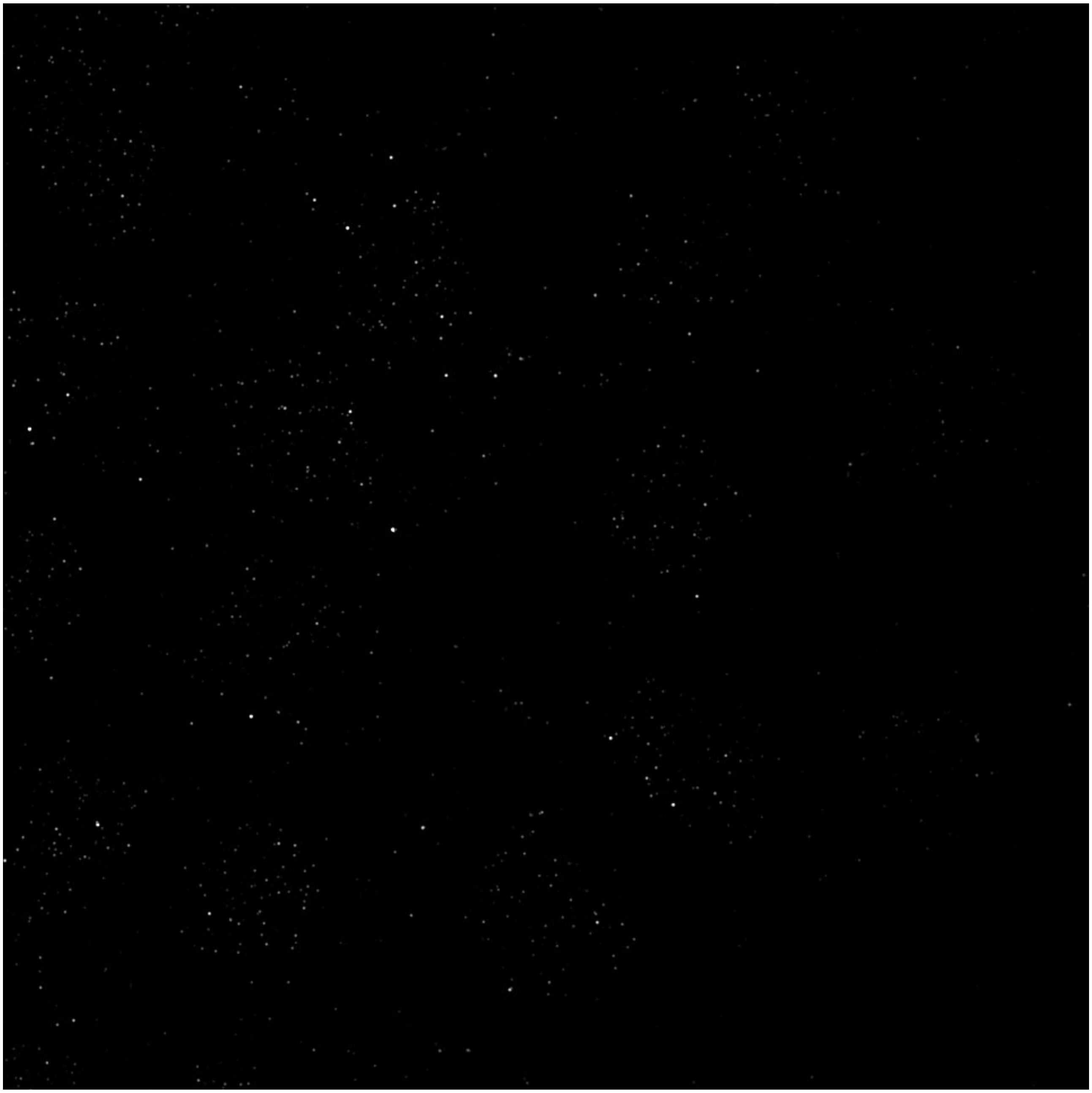

Fig4D_2_NoAddback_IAA_DOX_Ch1_Hoechst.jpg (1/1)

960 x 960

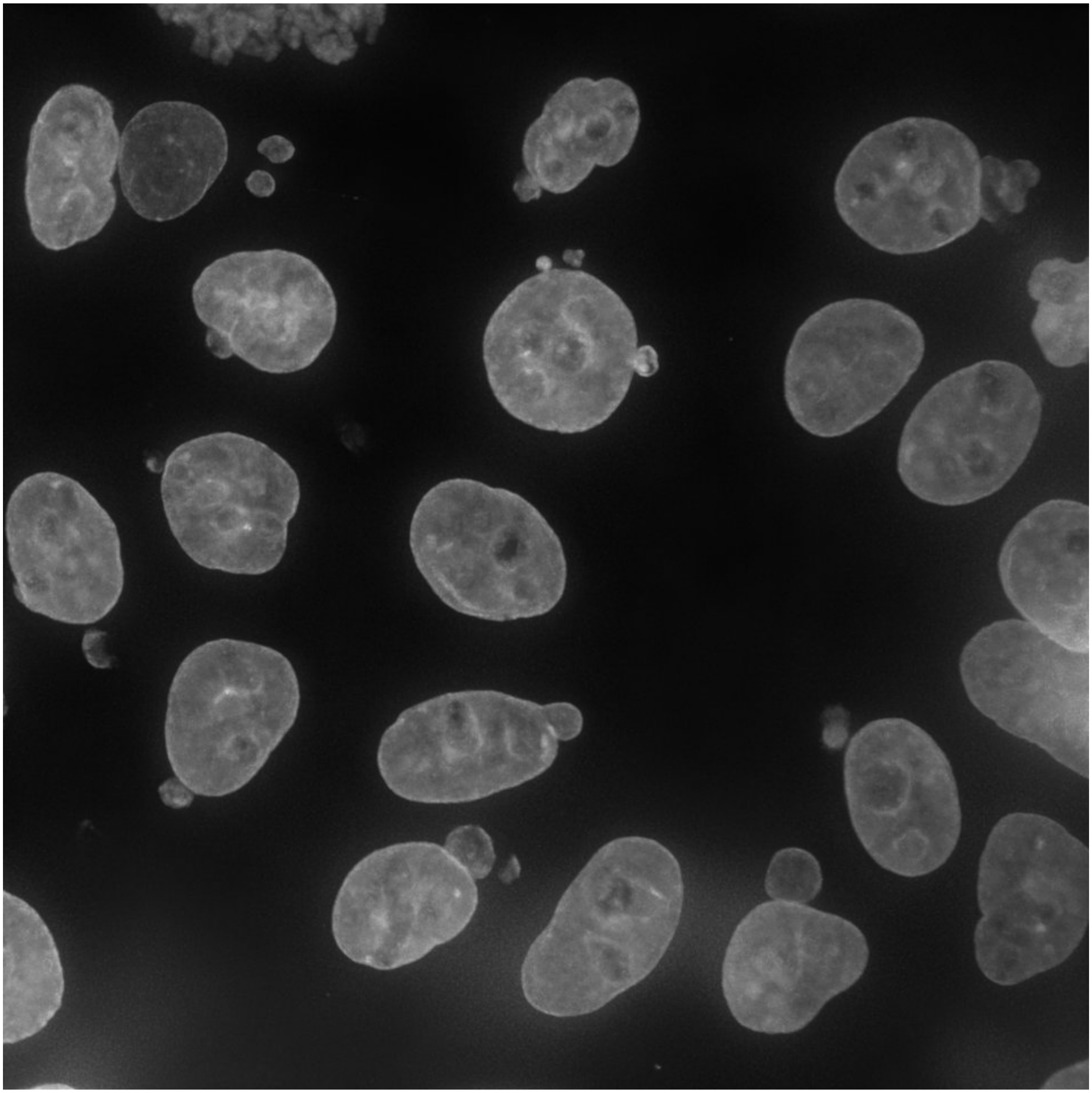

Fig4D_2_NoAddback_IAA_DOX_Ch2_CREST.jpg (1/1)

960 x 960

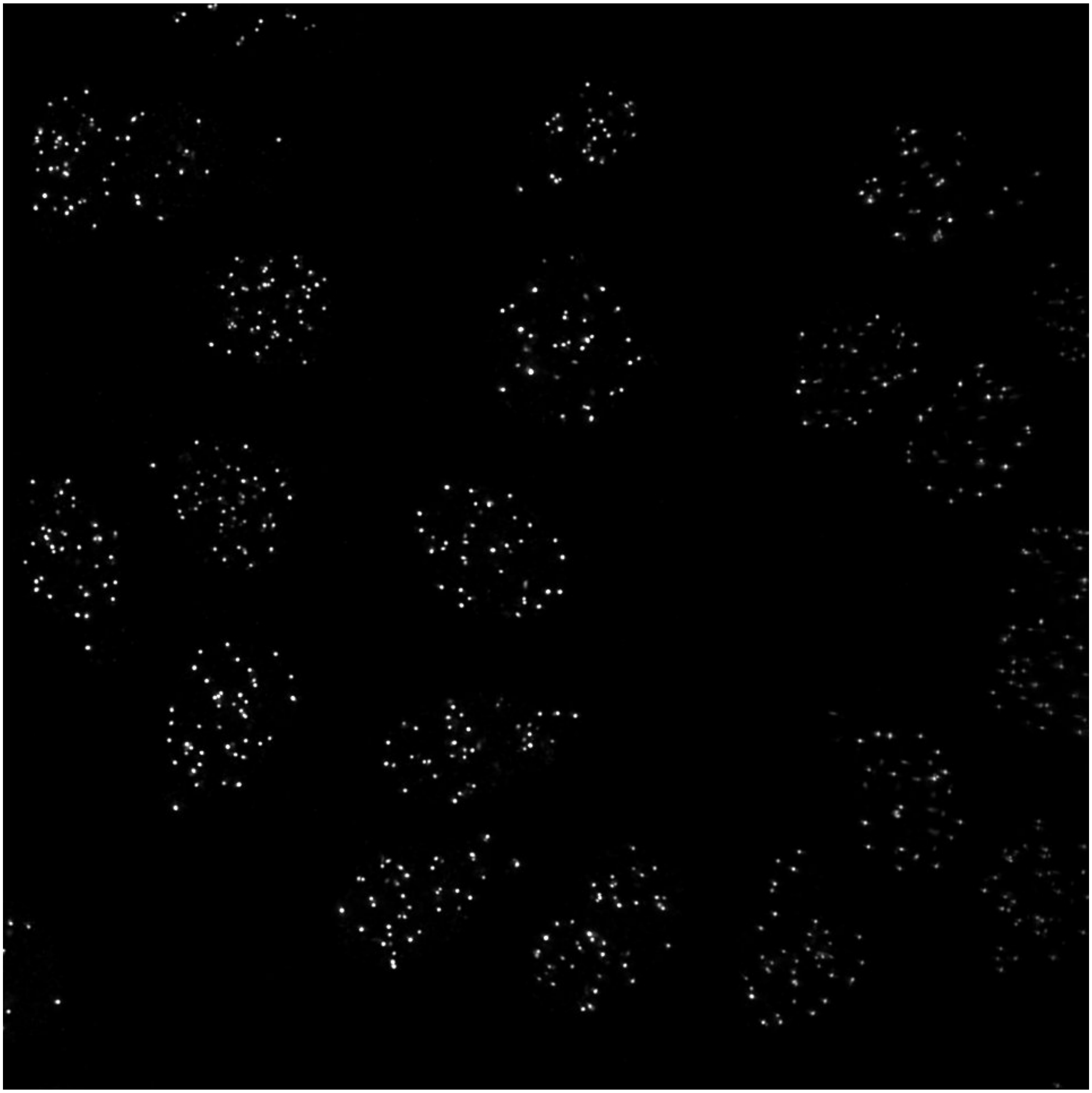

Fig4D_2_NoAddback_IAA_DOX_Ch3_GFP.jpg (1/1)
960 x 960

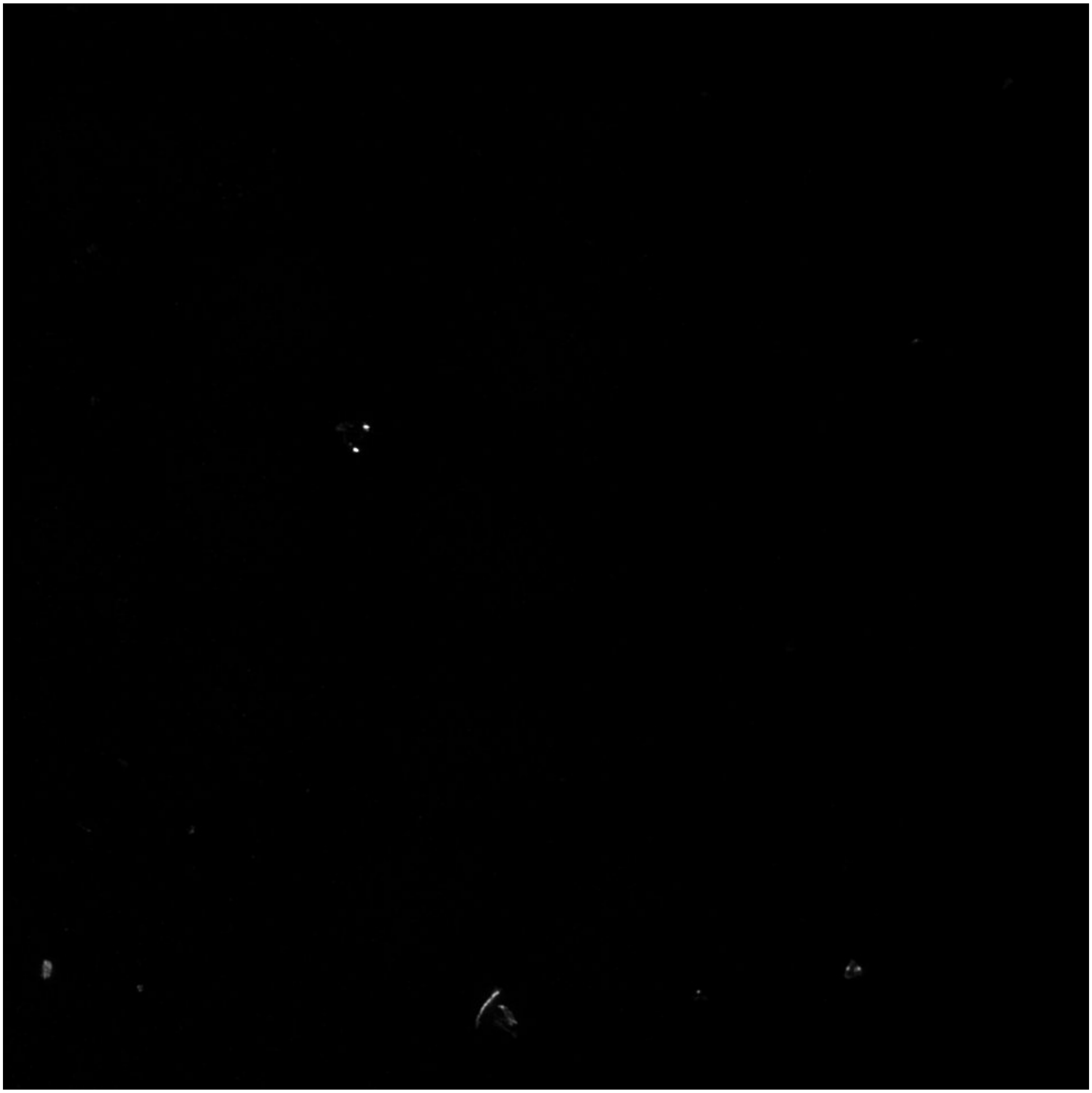

Fig4D_2_NoAddback_IAA_DOX_Ch4_mRuby.jpg (1/1)

960 x 960

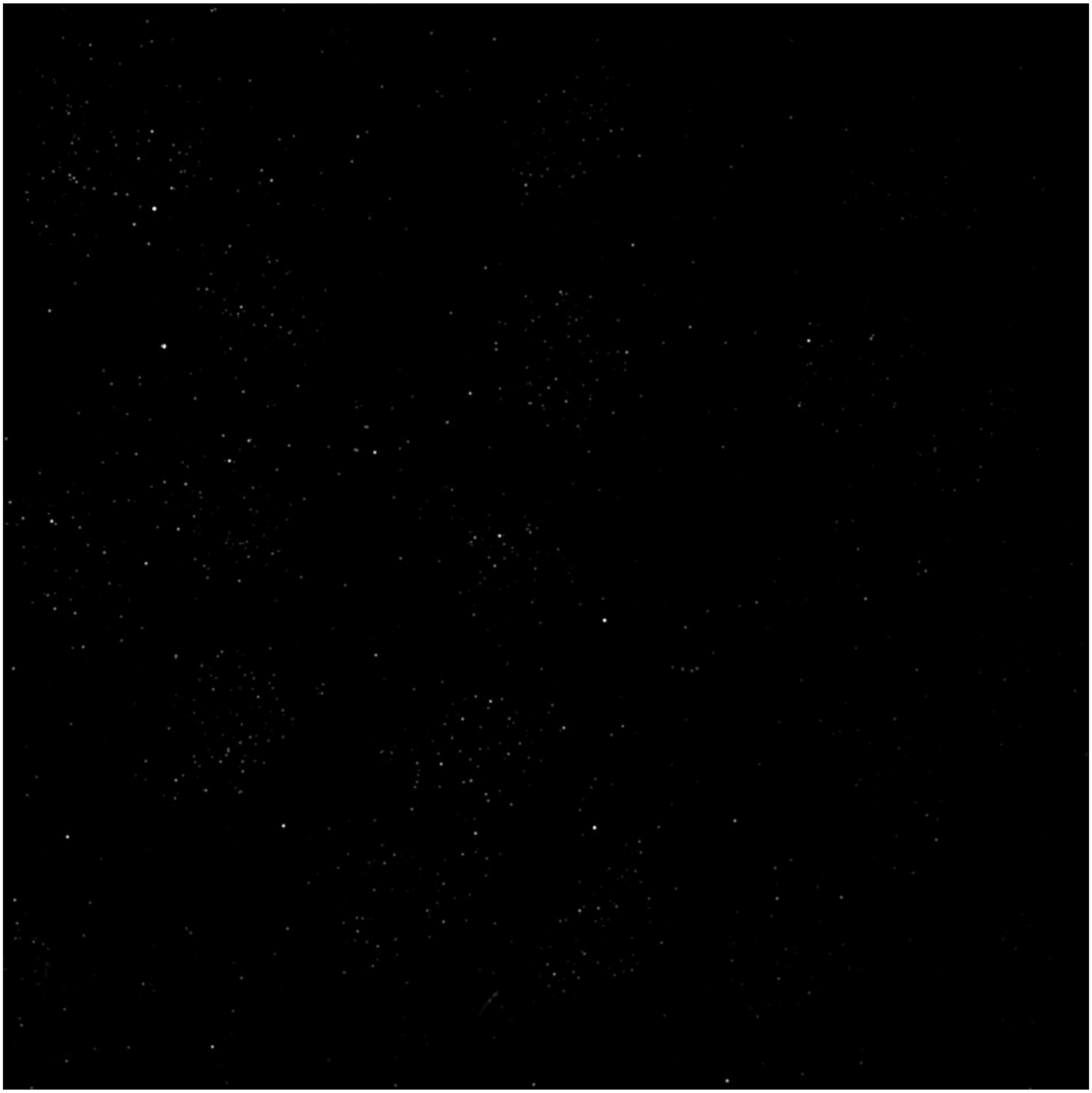

Fig4D_3_WT_Untreated_Ch1_Hoechst.jpg (1/1)
960 x 960

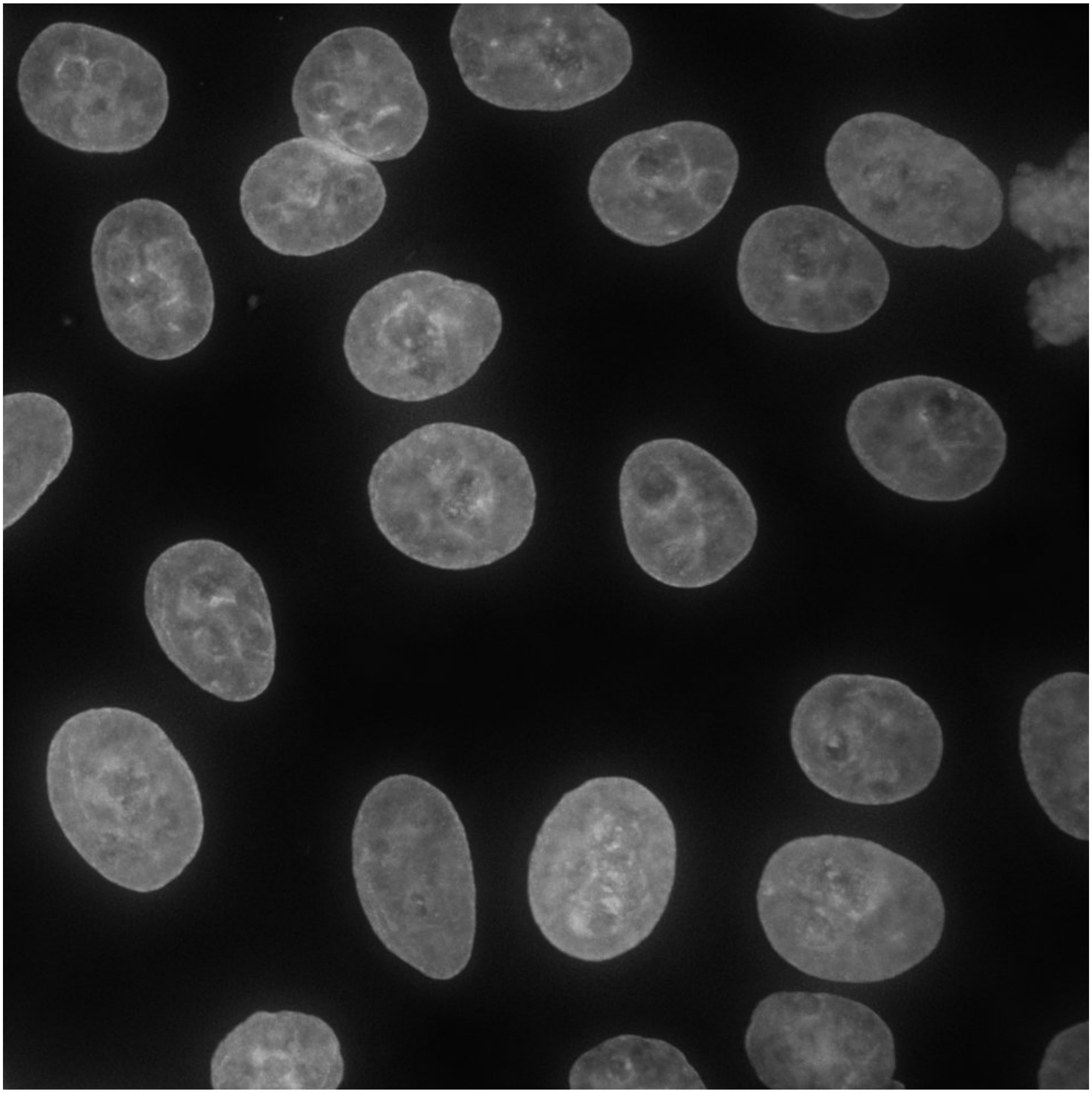

Fig4D_3_WT_Untreated_Ch2_CREST.jpg (1/1)
960 x 960

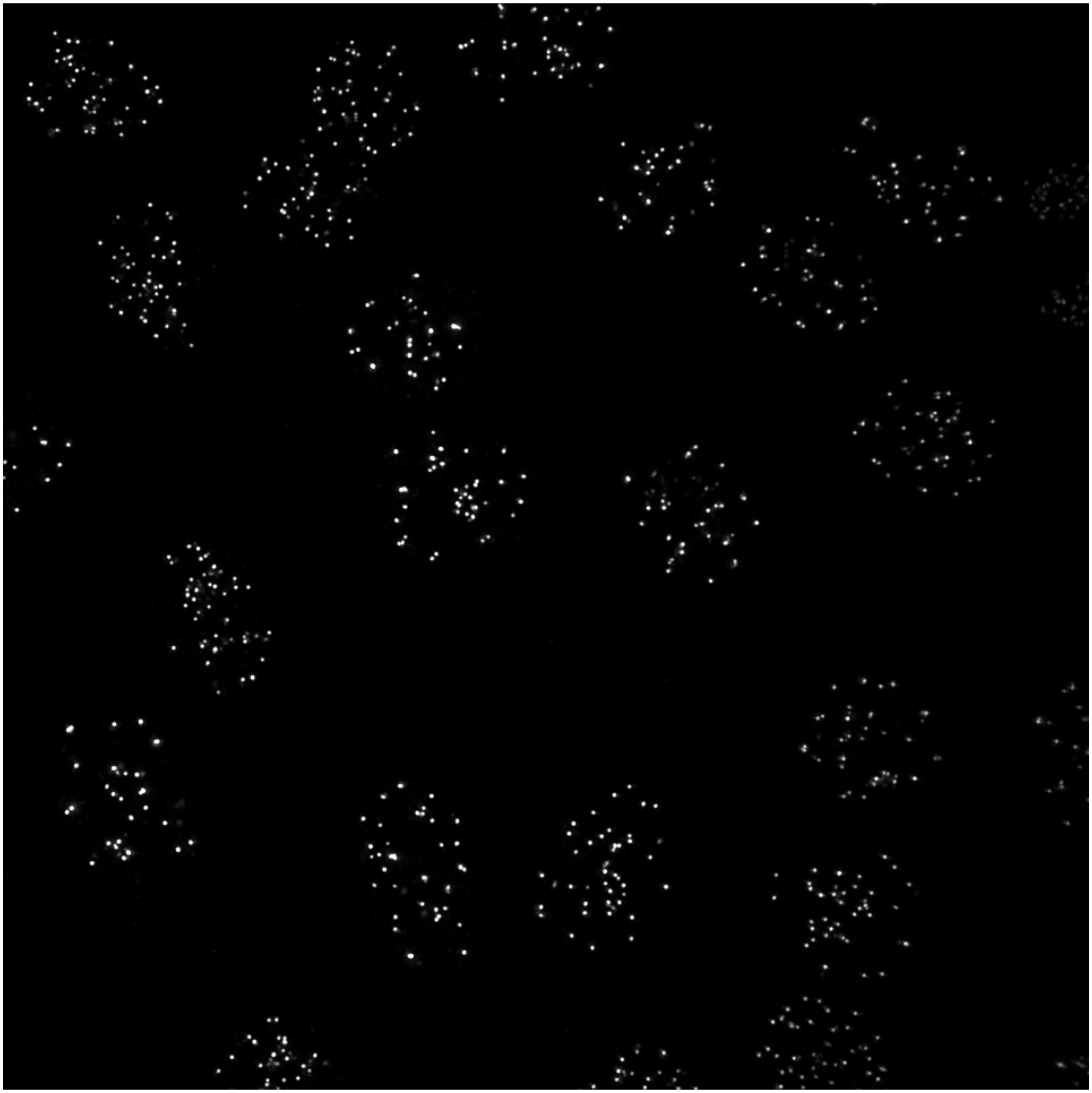

Fig4D_3_WT_Untreated_Ch3_GFP.jpg (1/1)

960 x 960

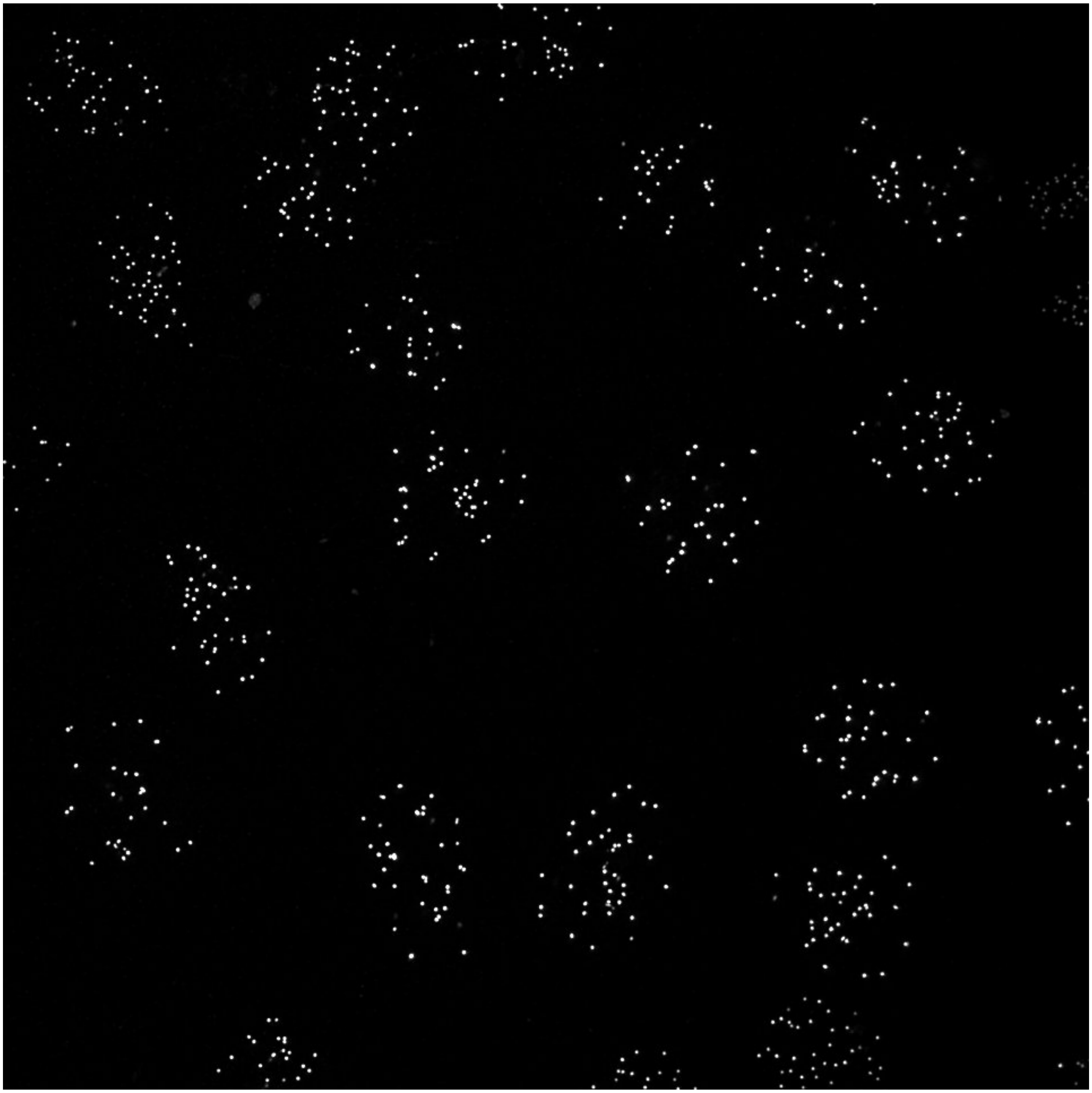

Fig4D_3_WT_Untreated_Ch4_mRuby.jpg (1/1)

960 x 960

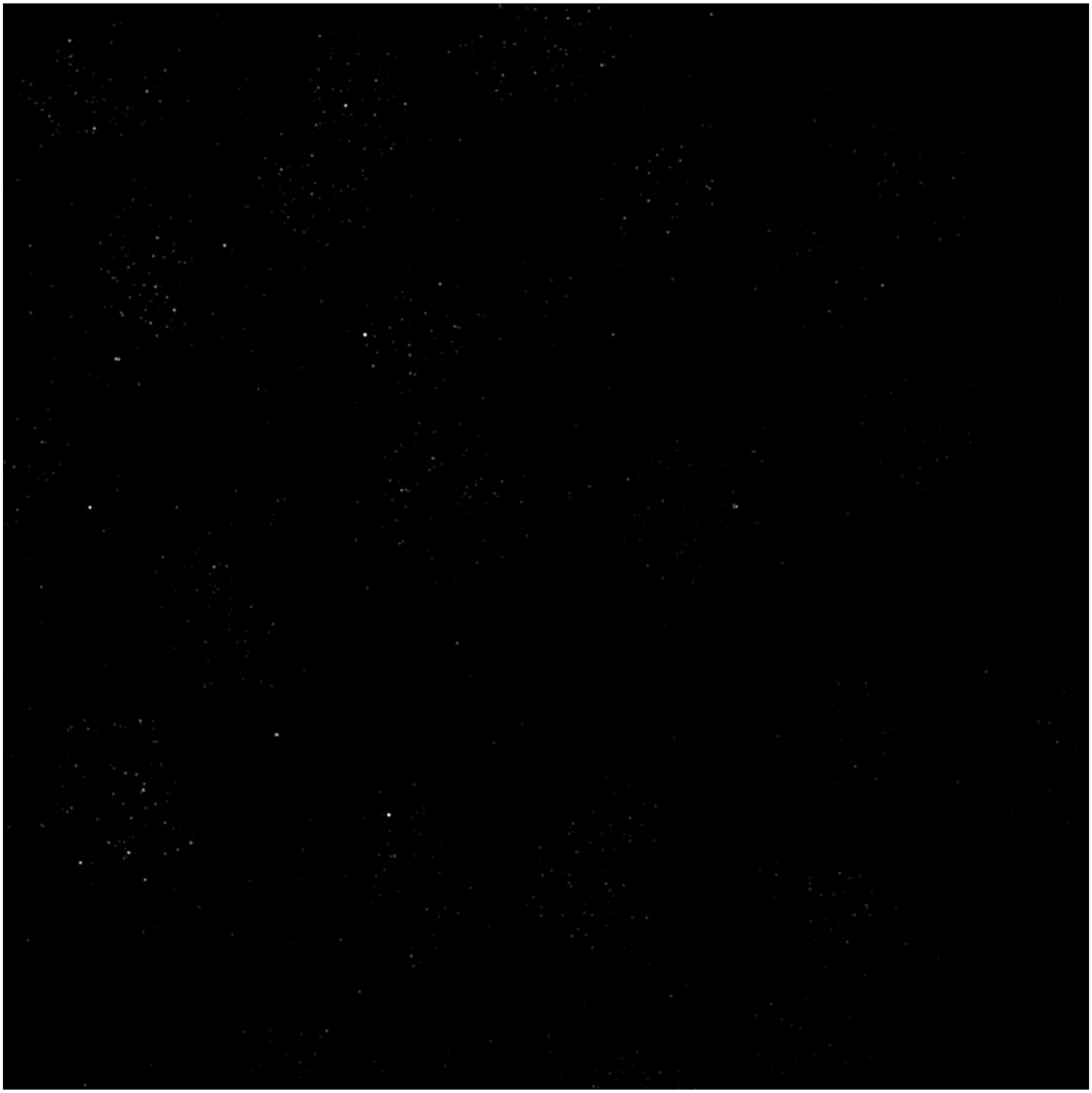

Fig4D_4_WT_IAA_DOX_Ch1_Hoechst.jpg (1/1)

960 x 960

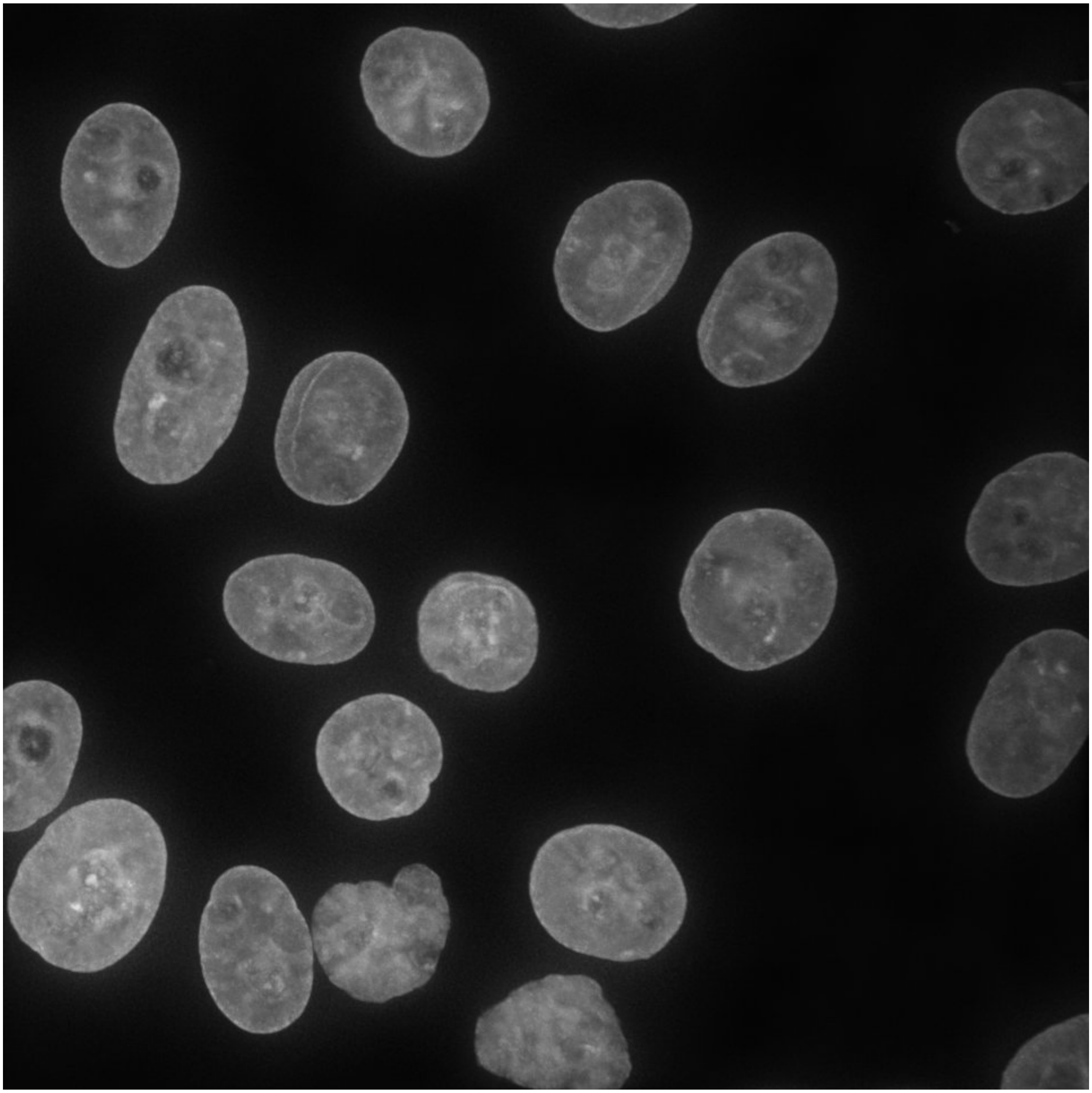

Fig4D_4_WT_IAA_DOX_Ch2_CREST.jpg (1/1)

960 x 960

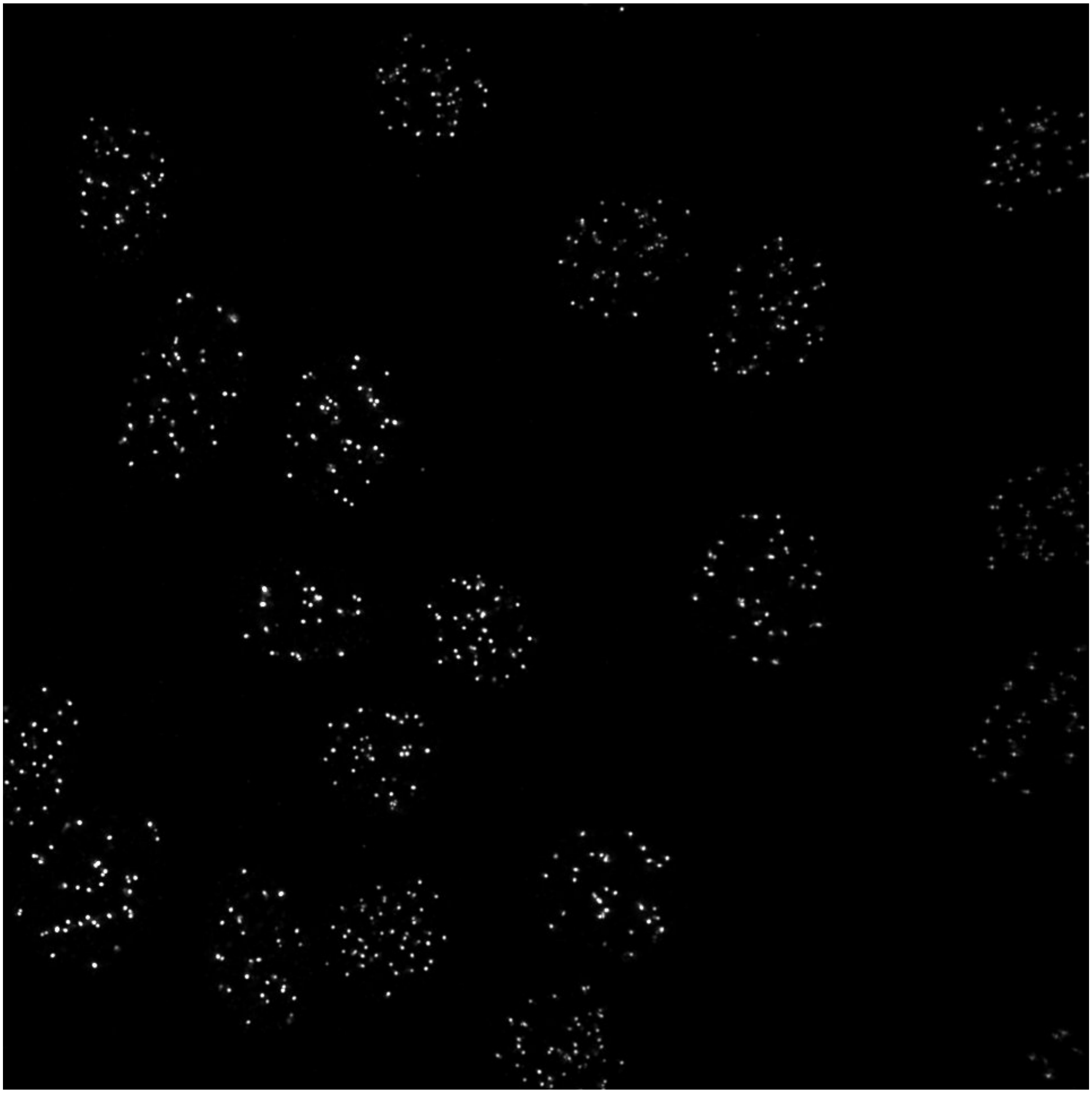

Fig4D_4_WT_IAA_DOX_Ch3_GFP.jpg (1/1)

960 x 960

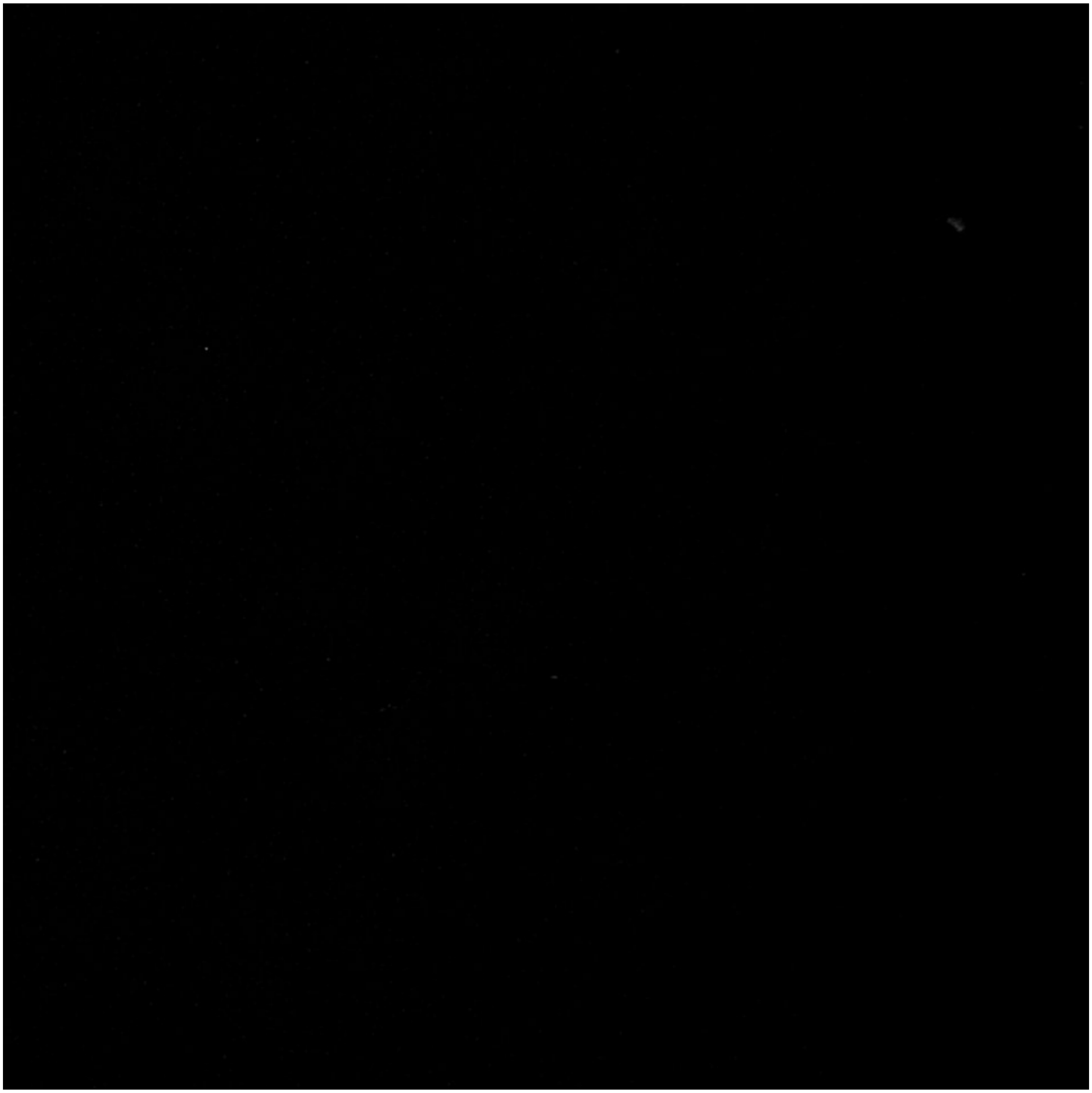

Fig4D_4_WT_IAA_DOX_Ch4_mRuby.jpg (1/1)
960 x 960

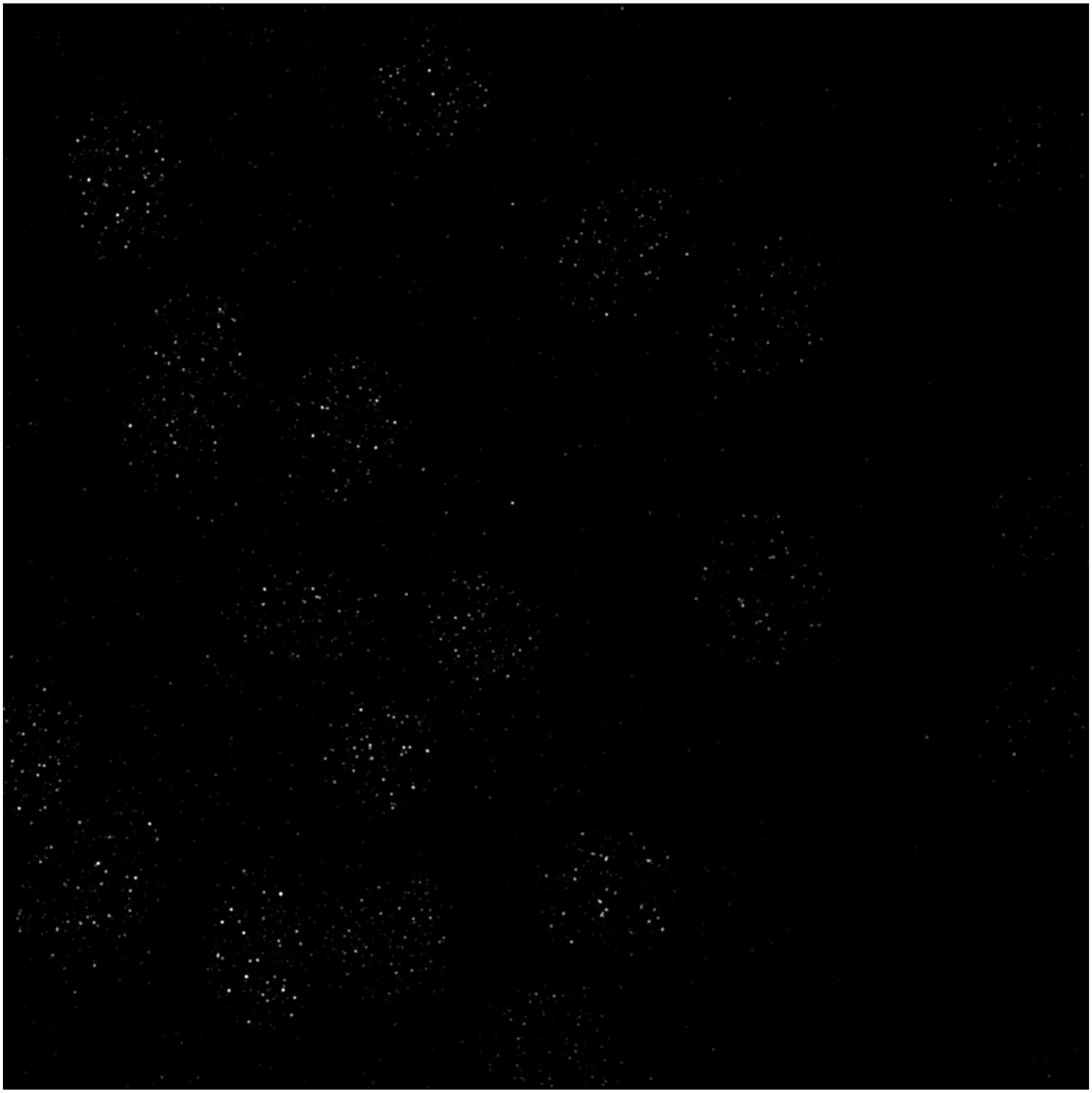

Fig4D_5_7ala_Untreated_Ch1_Hoechst.jpg (1/1)
960 x 960

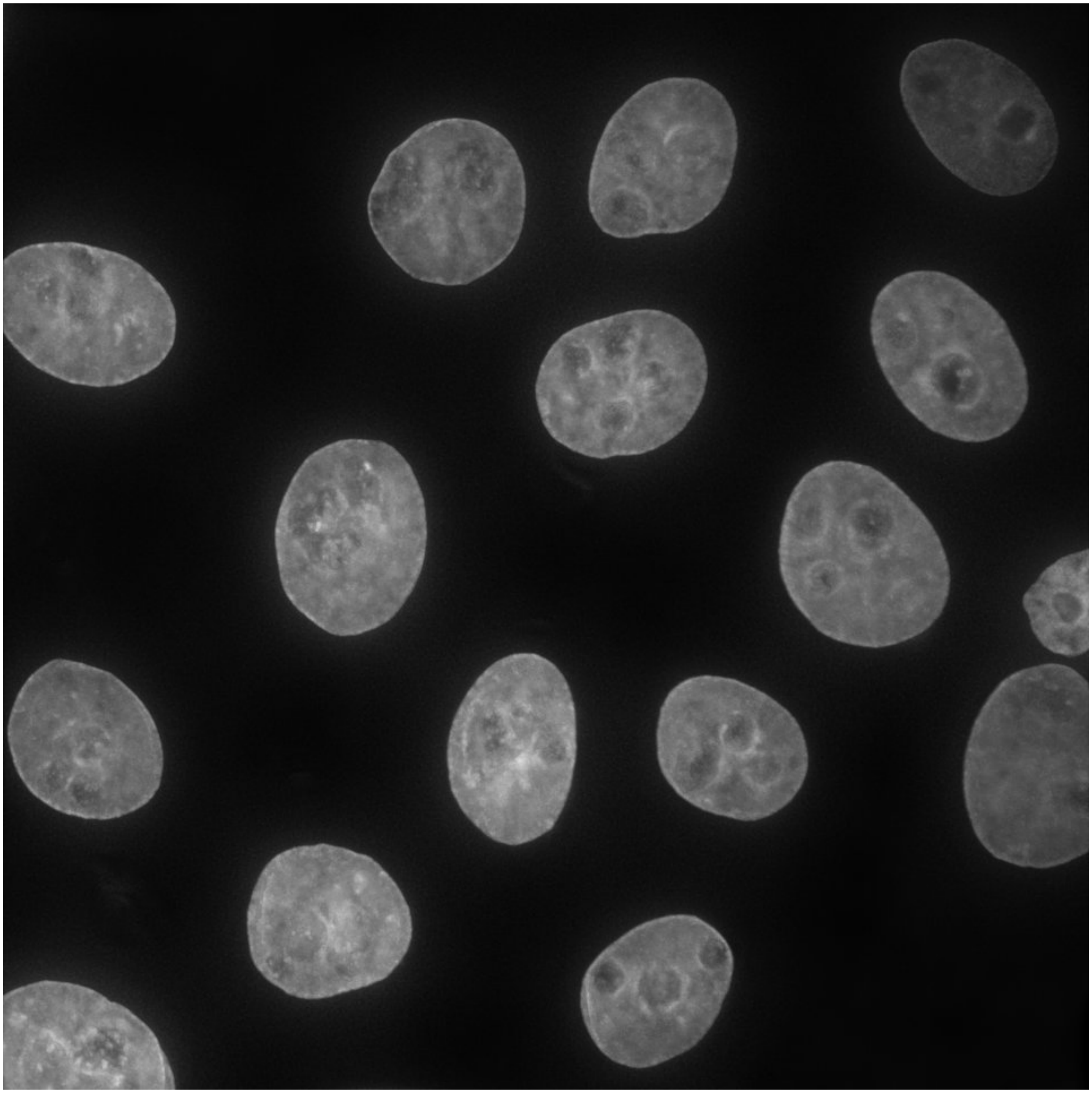

Fig4D_5_7ala_Untreated_Ch2_CREST.jpg (1/1)

960 x 960

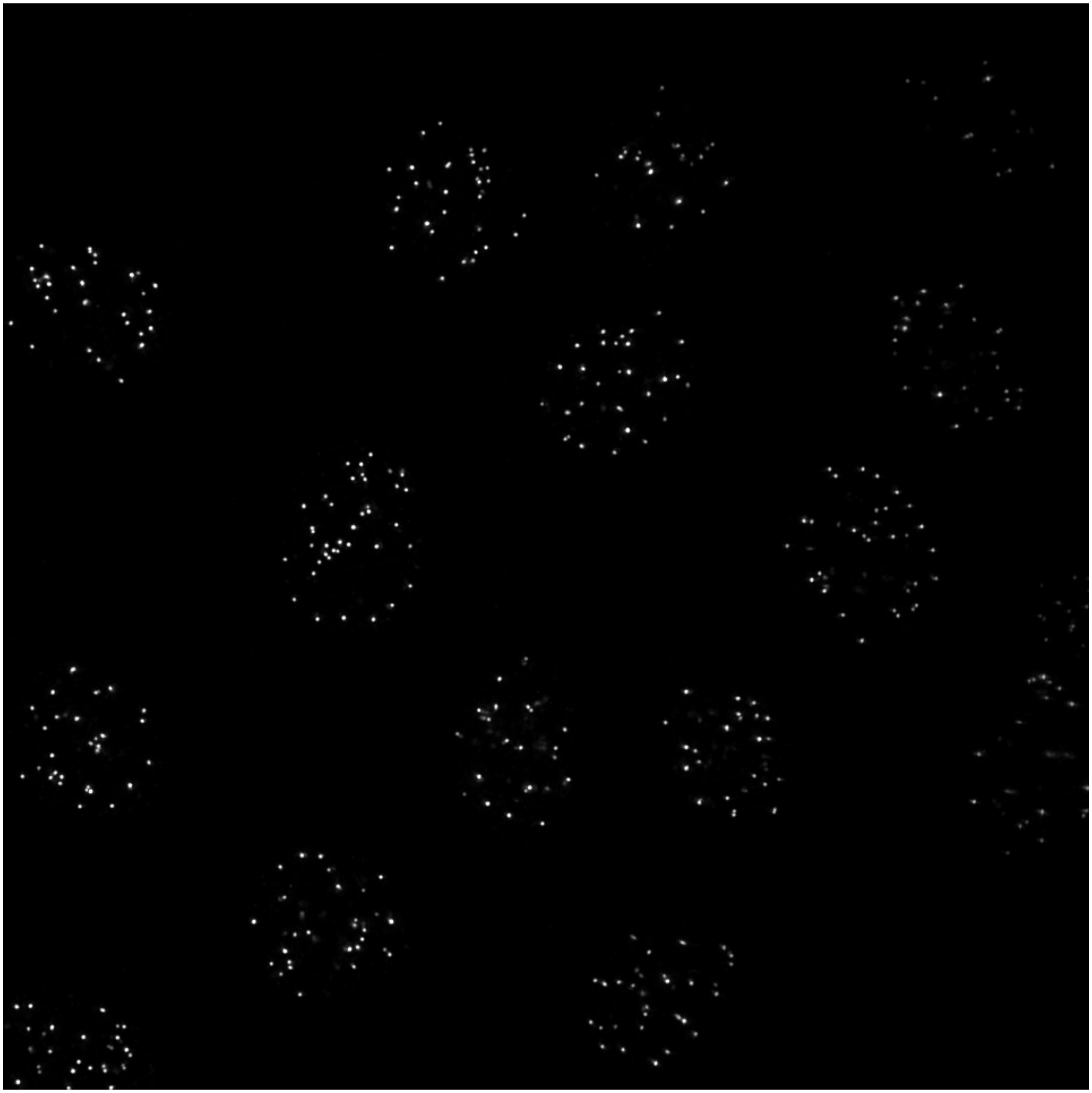

Fig4D_5_7ala_Untreated_Ch3_GFP.jpg (1/1)
960 x 960

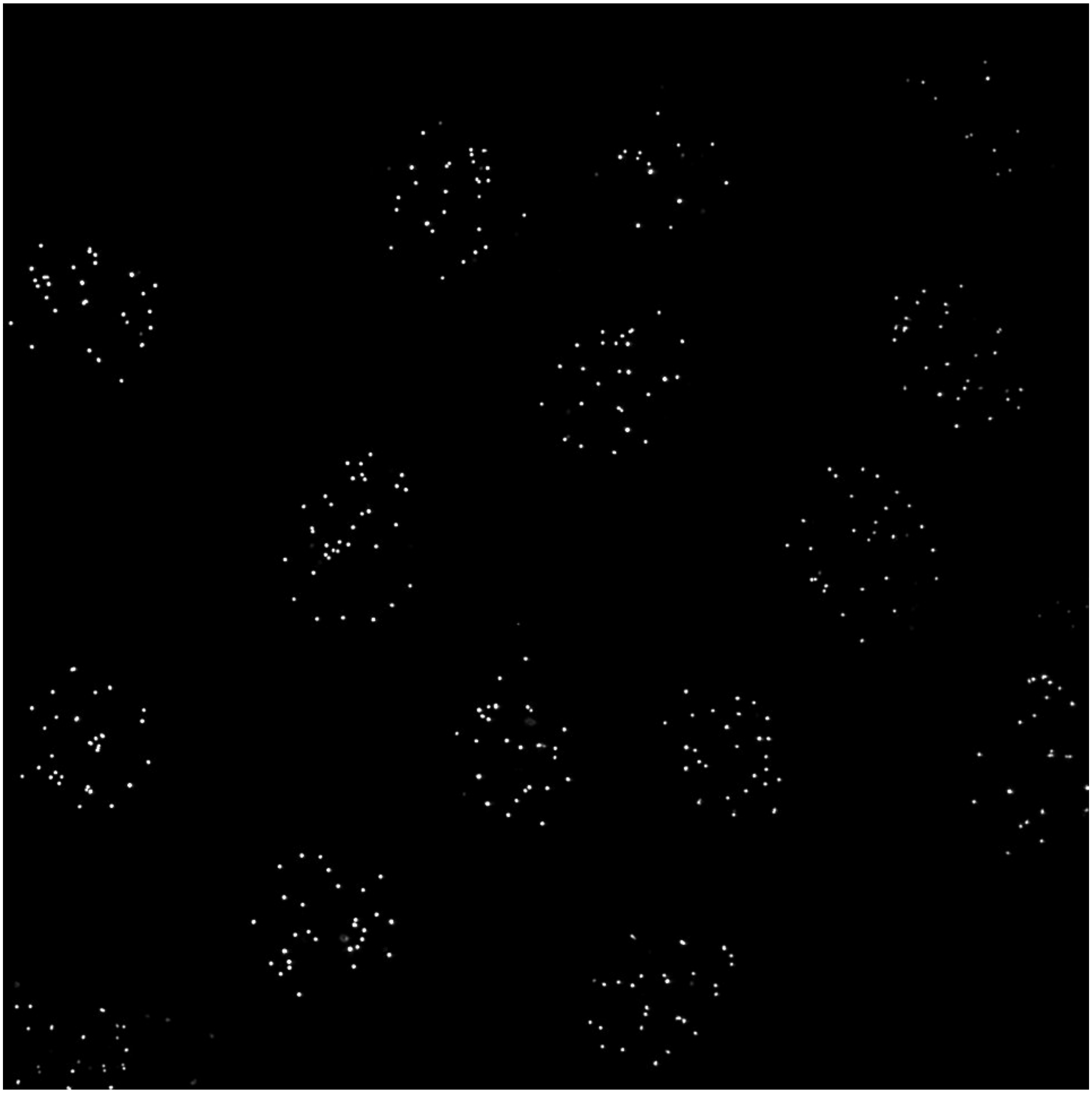

Fig4D_5_7ala_Untreated_Ch4_mRuby.jpg (1/1)

960 x 960

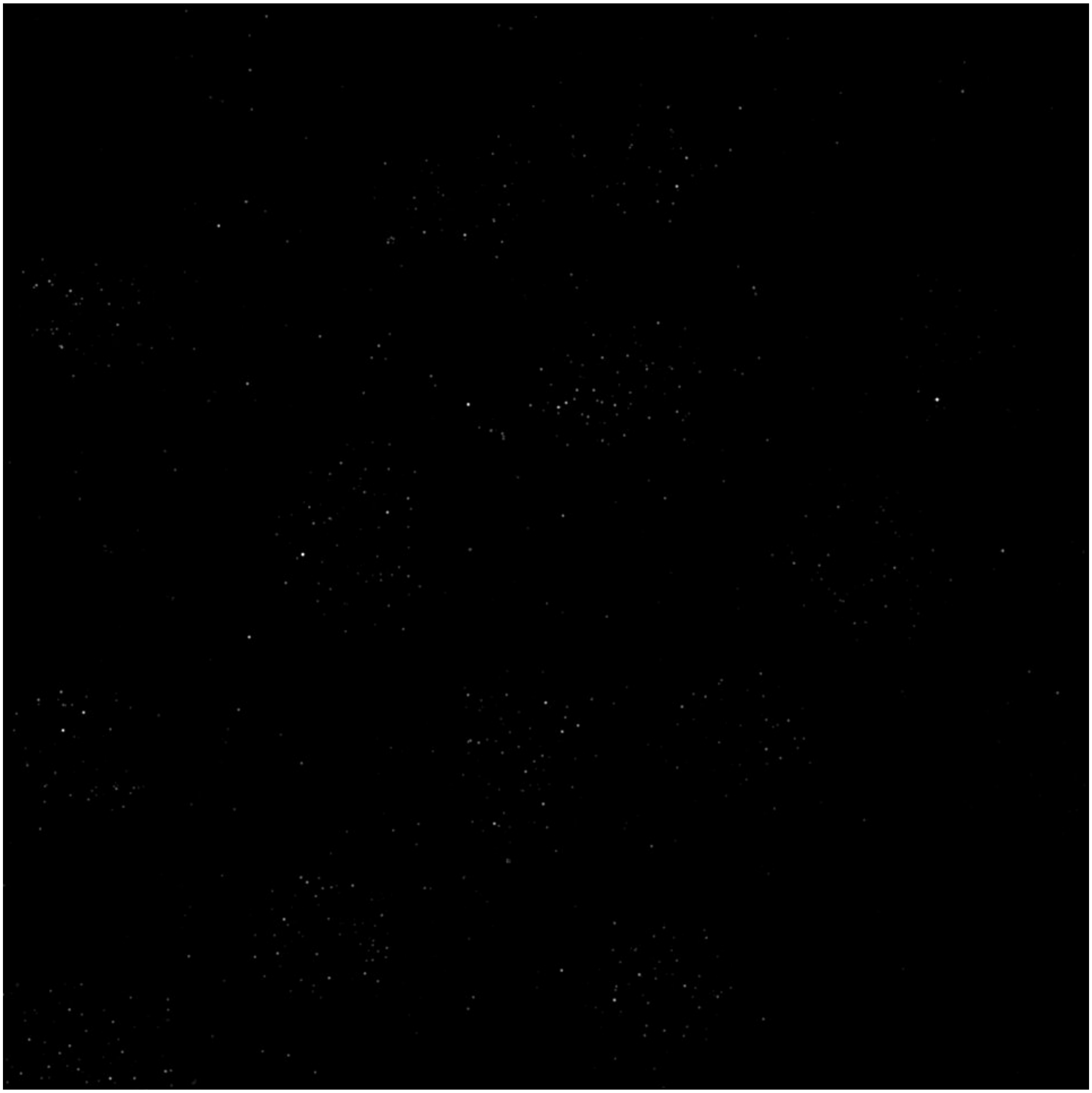

Fig4D_6_7ala_IAA_DOX_Ch1_Hoechst.jpg (1/1)
960 x 960

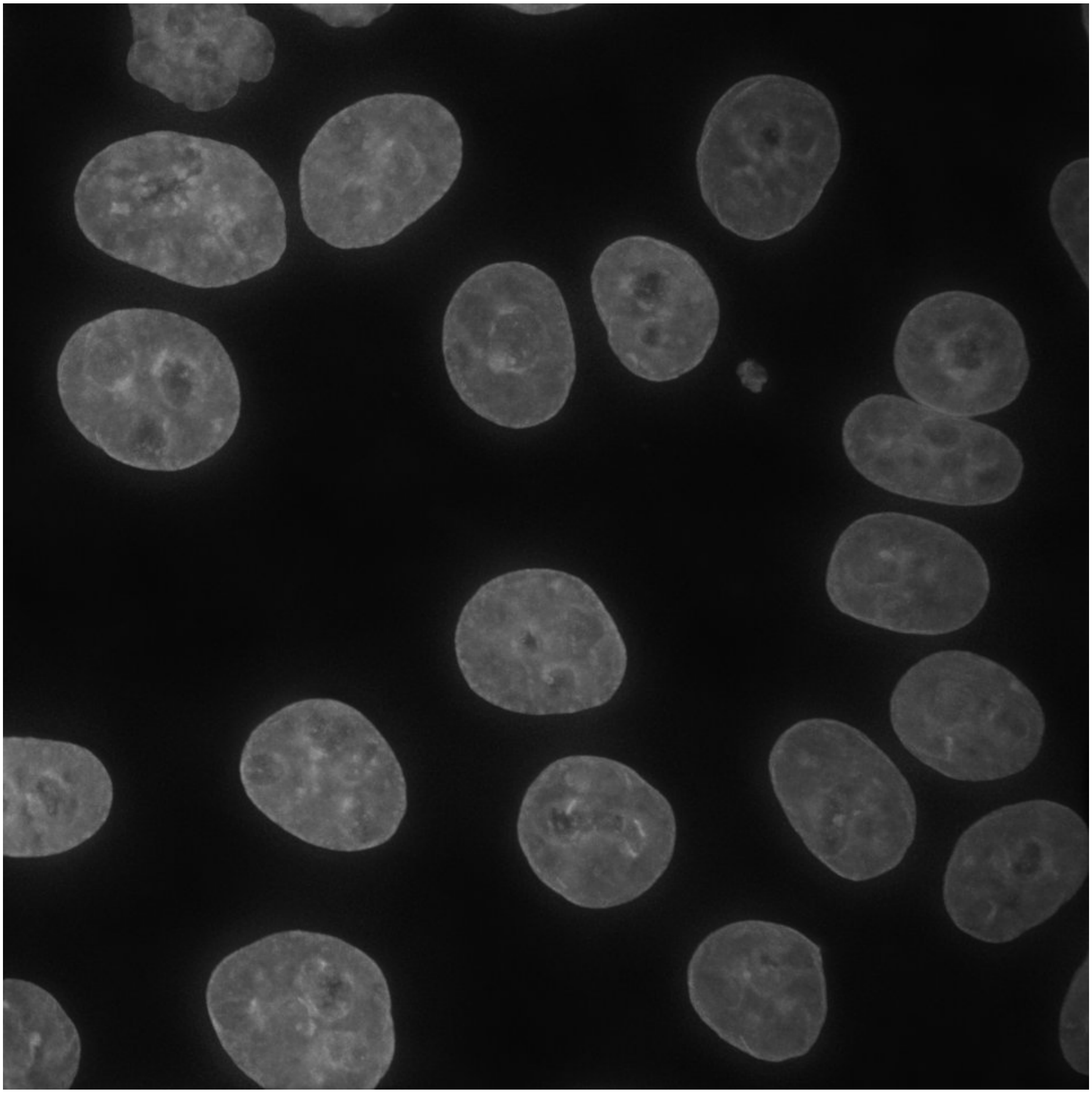

Fig4D_6_7ala_IAA_DOX_Ch2_CREST.jpg (1/1)
960 x 960

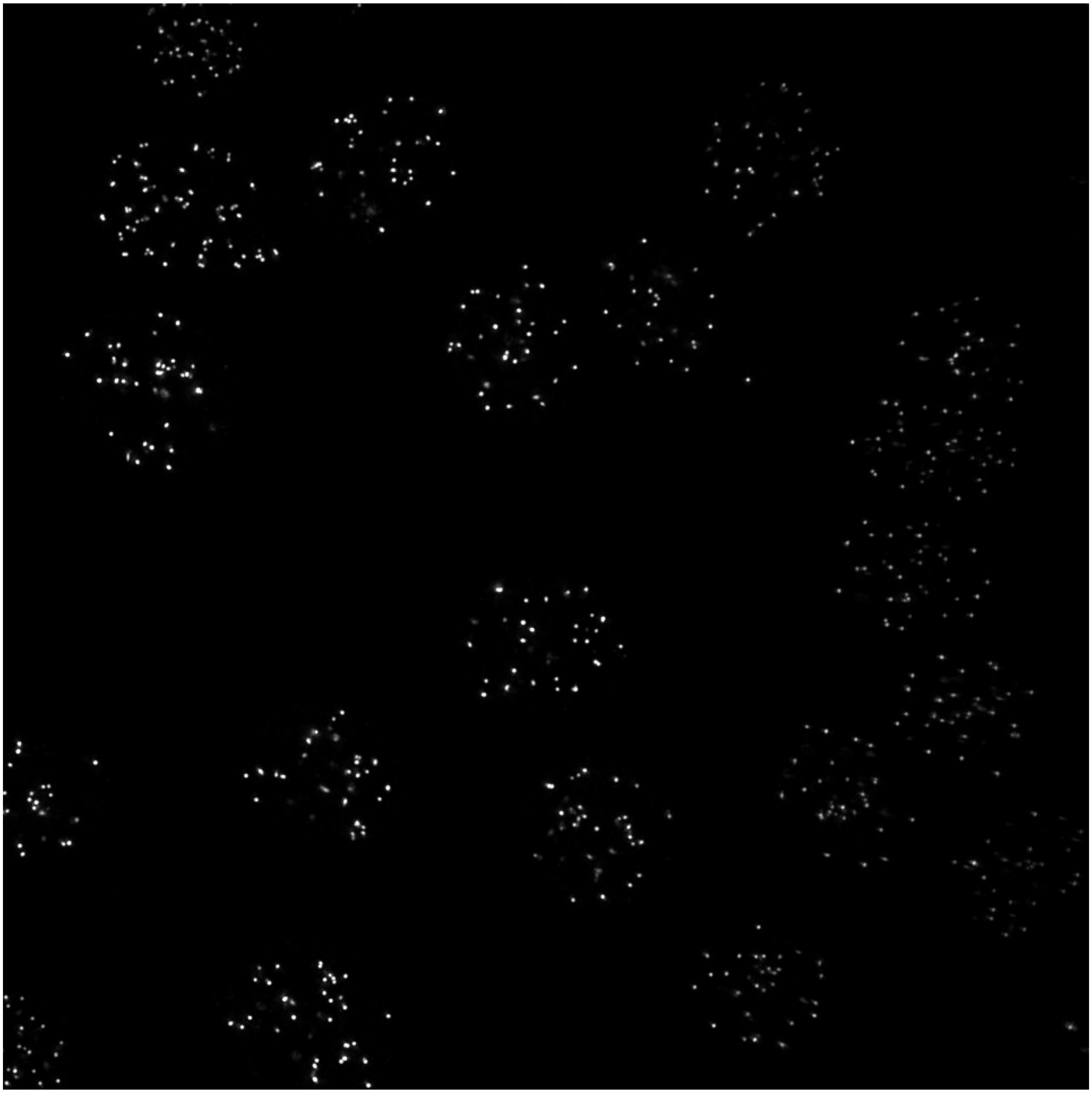

Fig4D_6_7ala_IAA_DOX_Ch3_GFP.jpg (1/1)

960 x 960

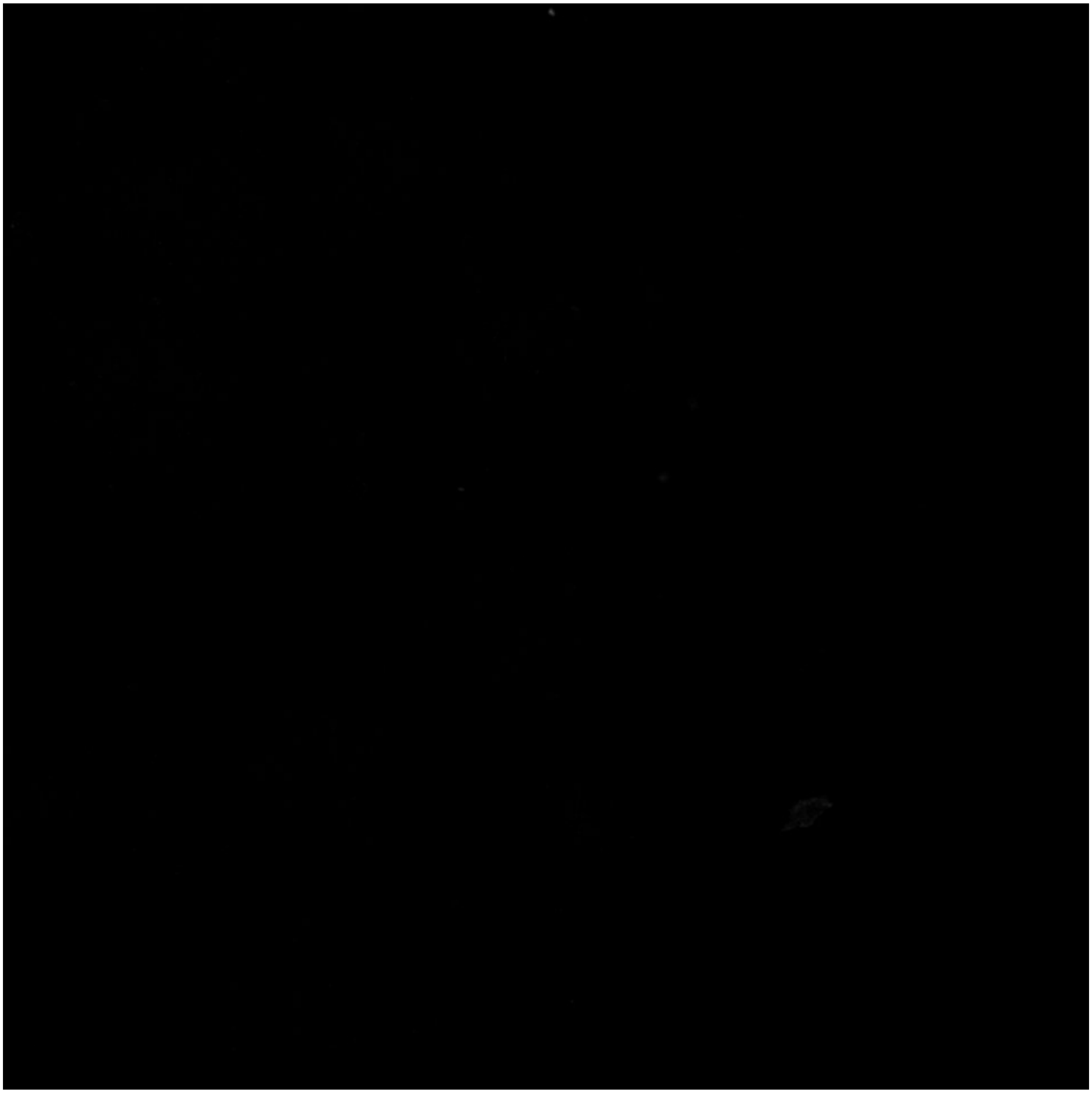

Fig4D_6_7ala_IAA_DOX_Ch4_mRuby.jpg (1/1)

960 x 960

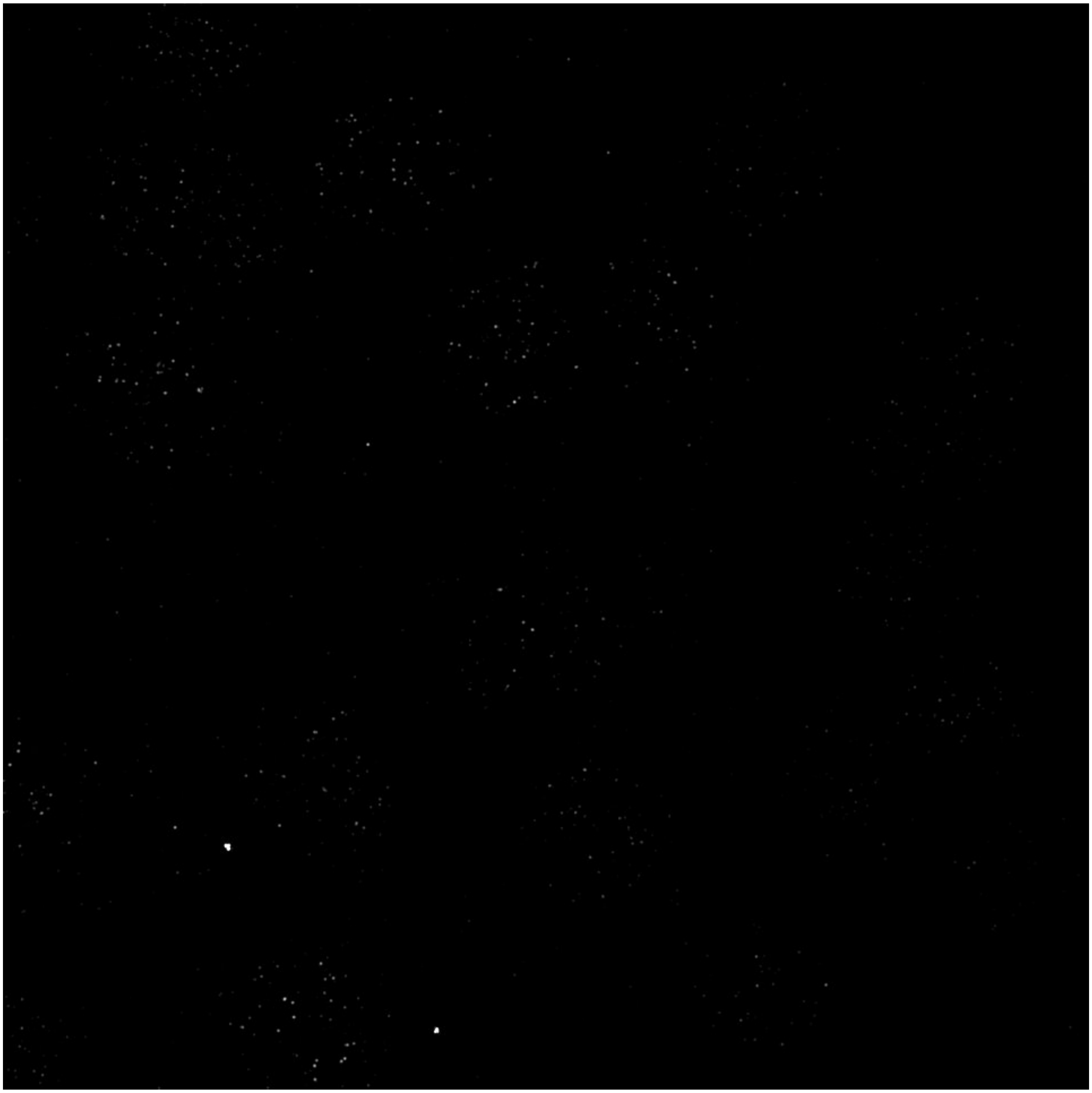

Fig4D_7_K102A_Untreated_Ch1_Hoechst.jpg (1/1)

960 x 960

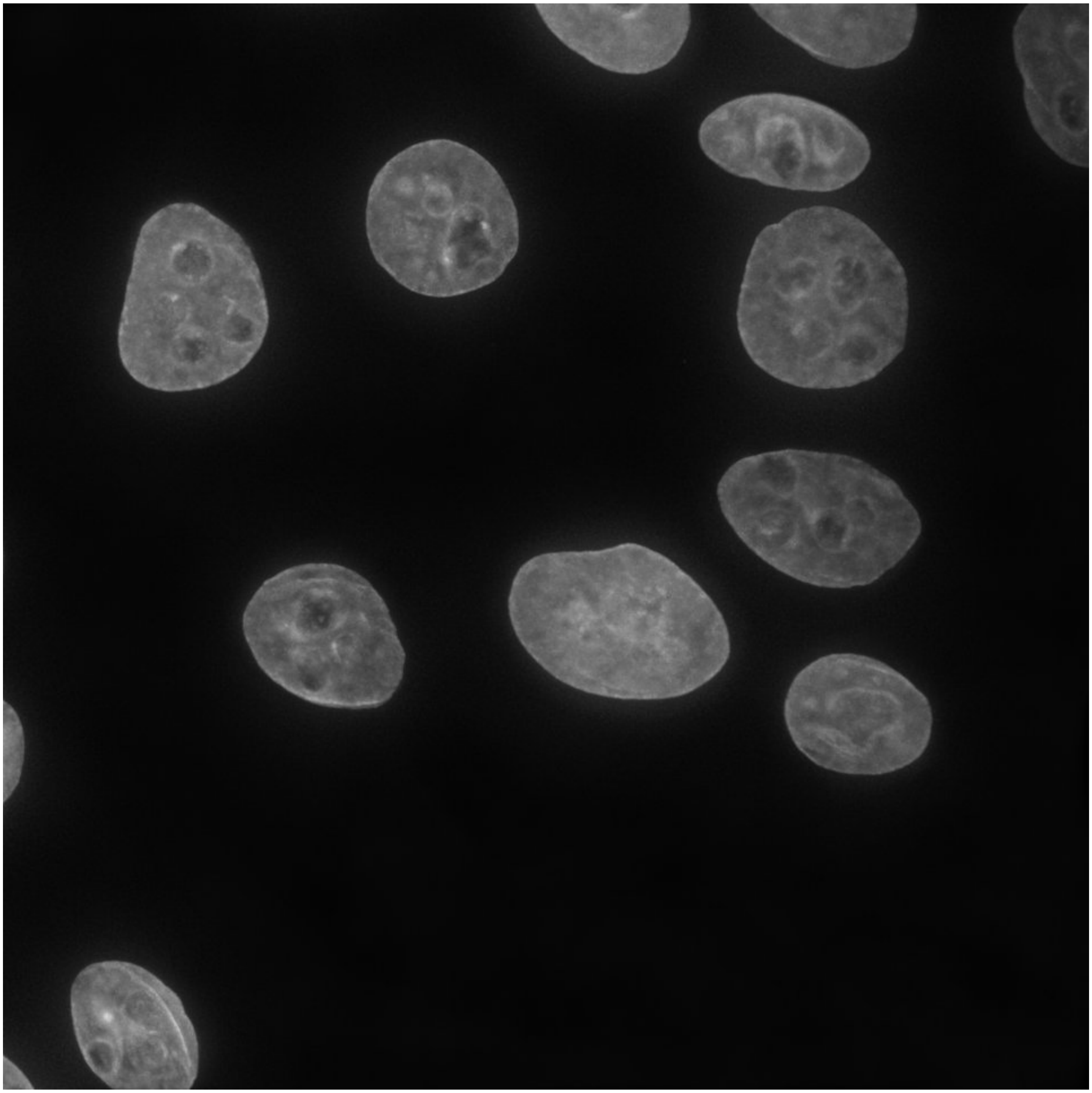

Fig4D_7_K102A_Untreated_Ch2_CREST.jpg (1/1)

960 x 960

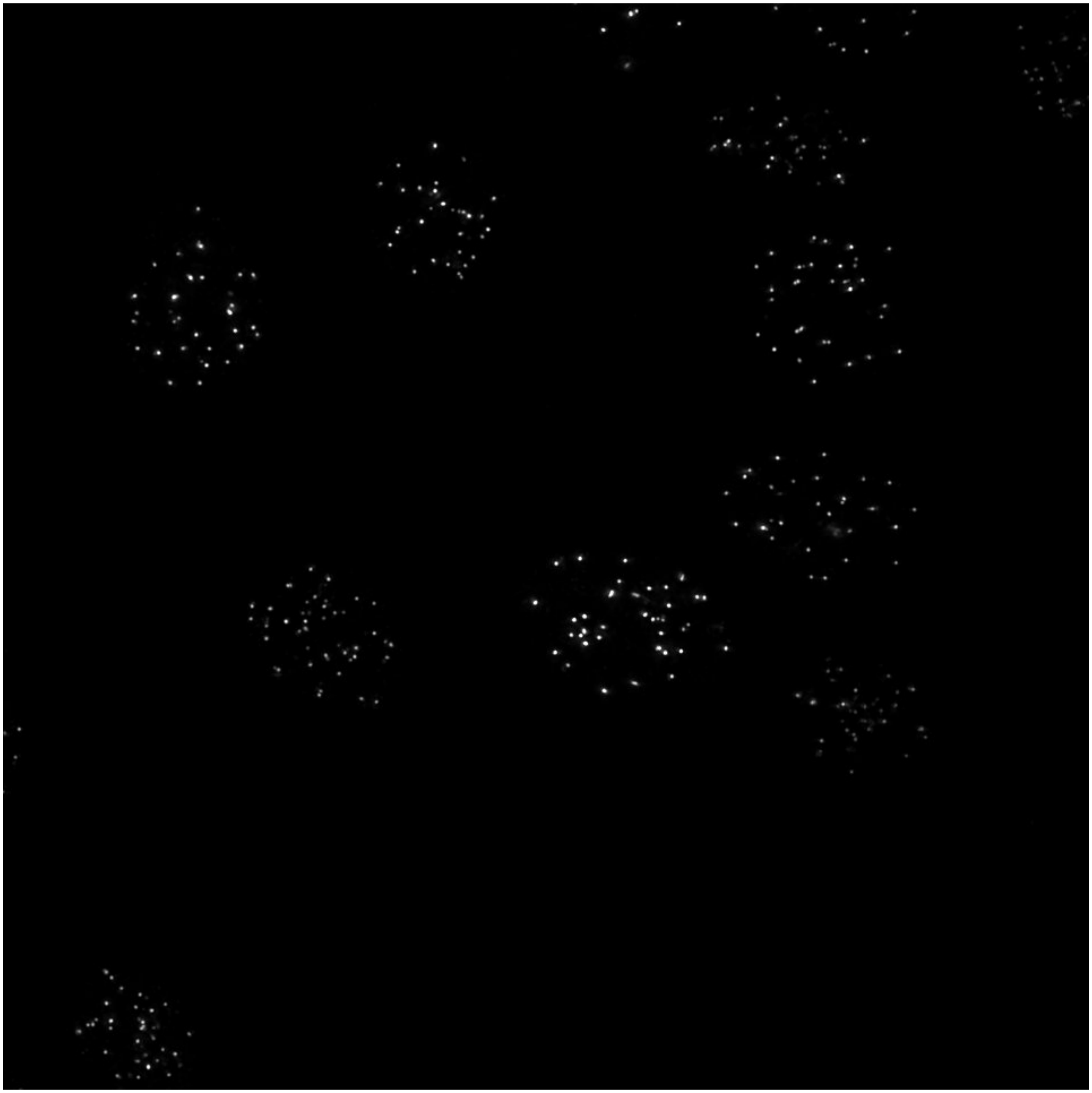

Fig4D_7_K102A_Untreated_Ch3_GFP.jpg (1/1)

960 x 960

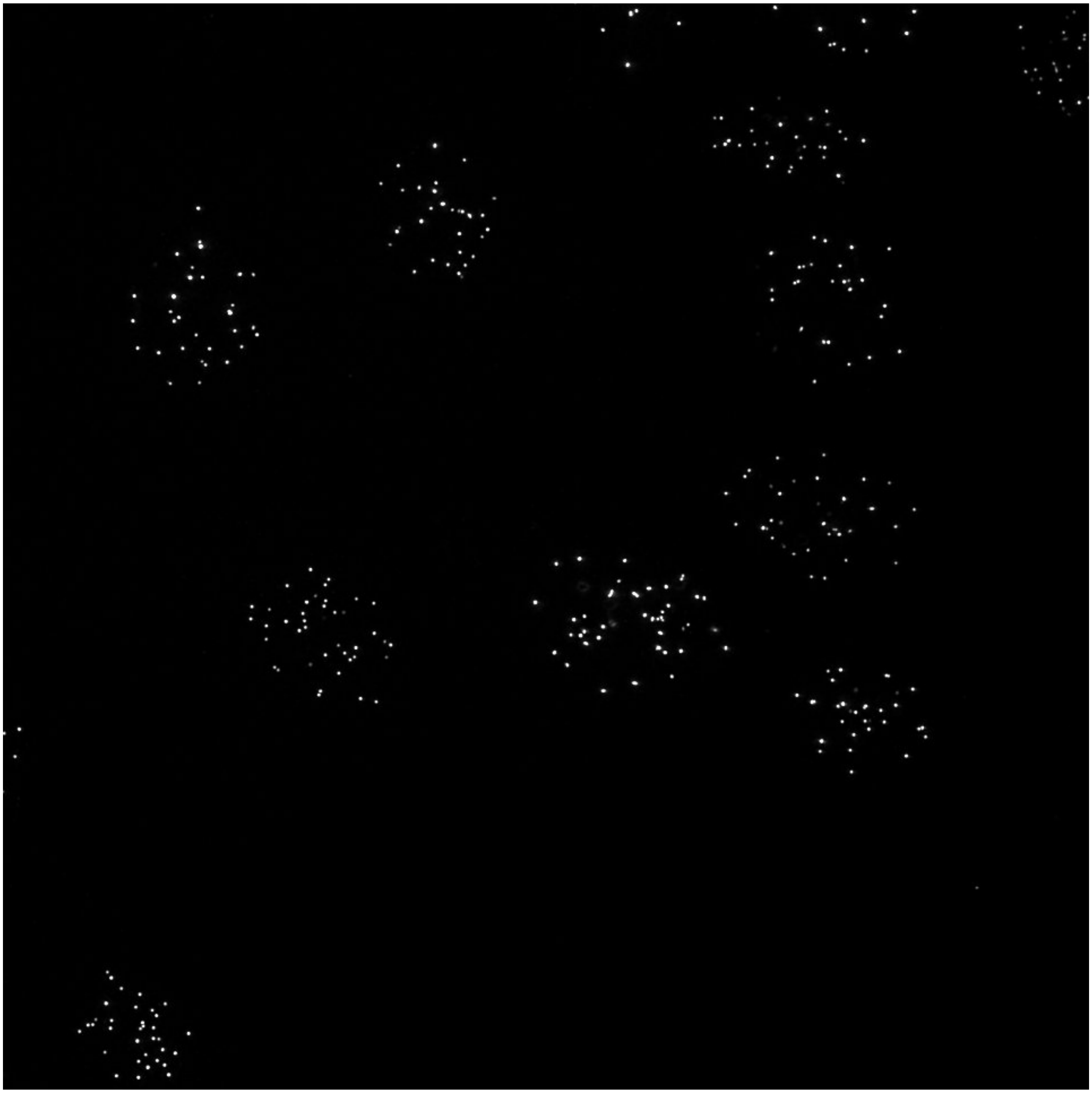

Fig4D_7_K102A_Untreated_Ch4_mRuby.jpg (1/1)
960 x 960

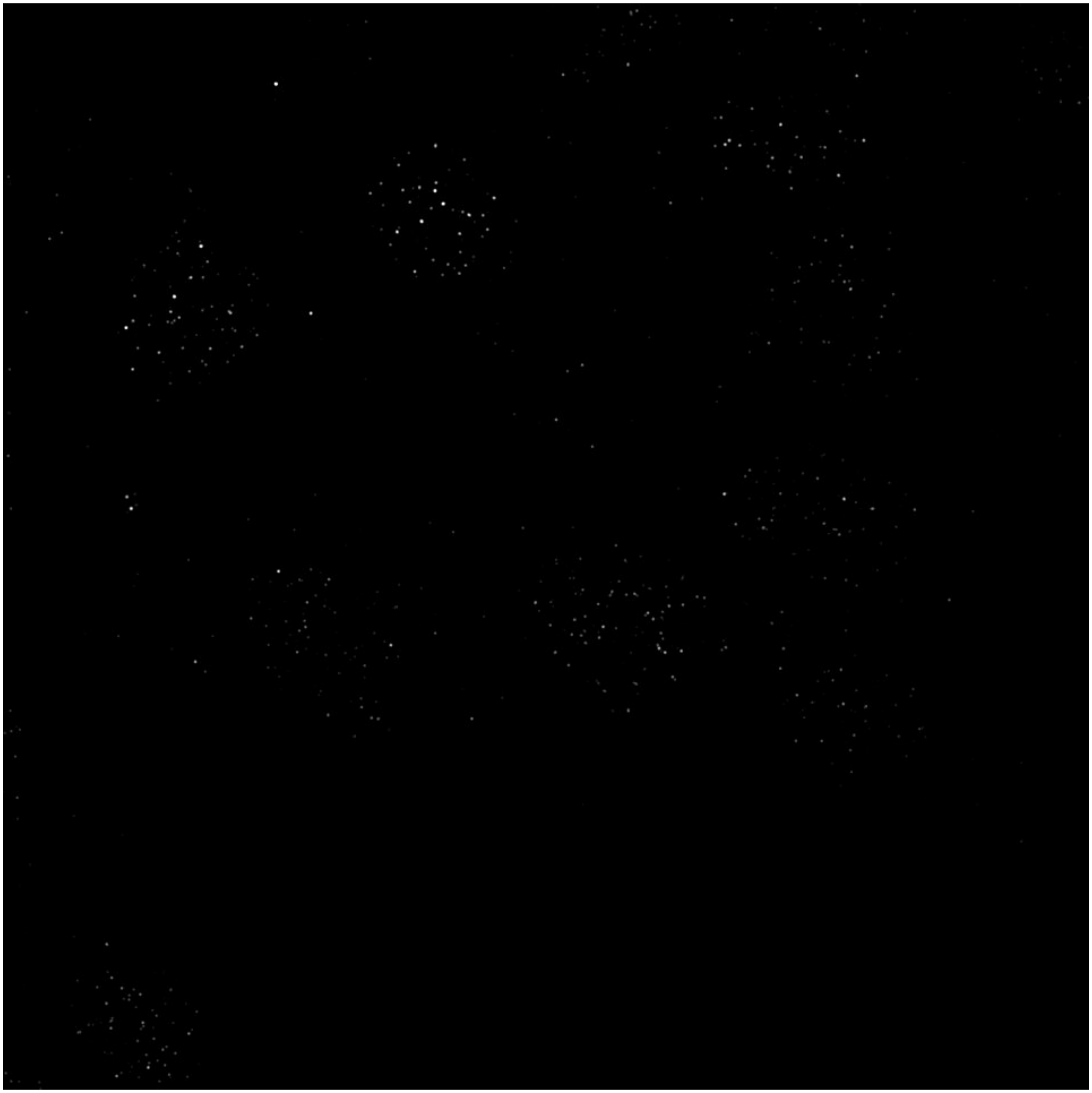

Fig4D_8_K102A_IAA_DOX_Ch1_Hoechst.jpg (1/1)

960 x 960

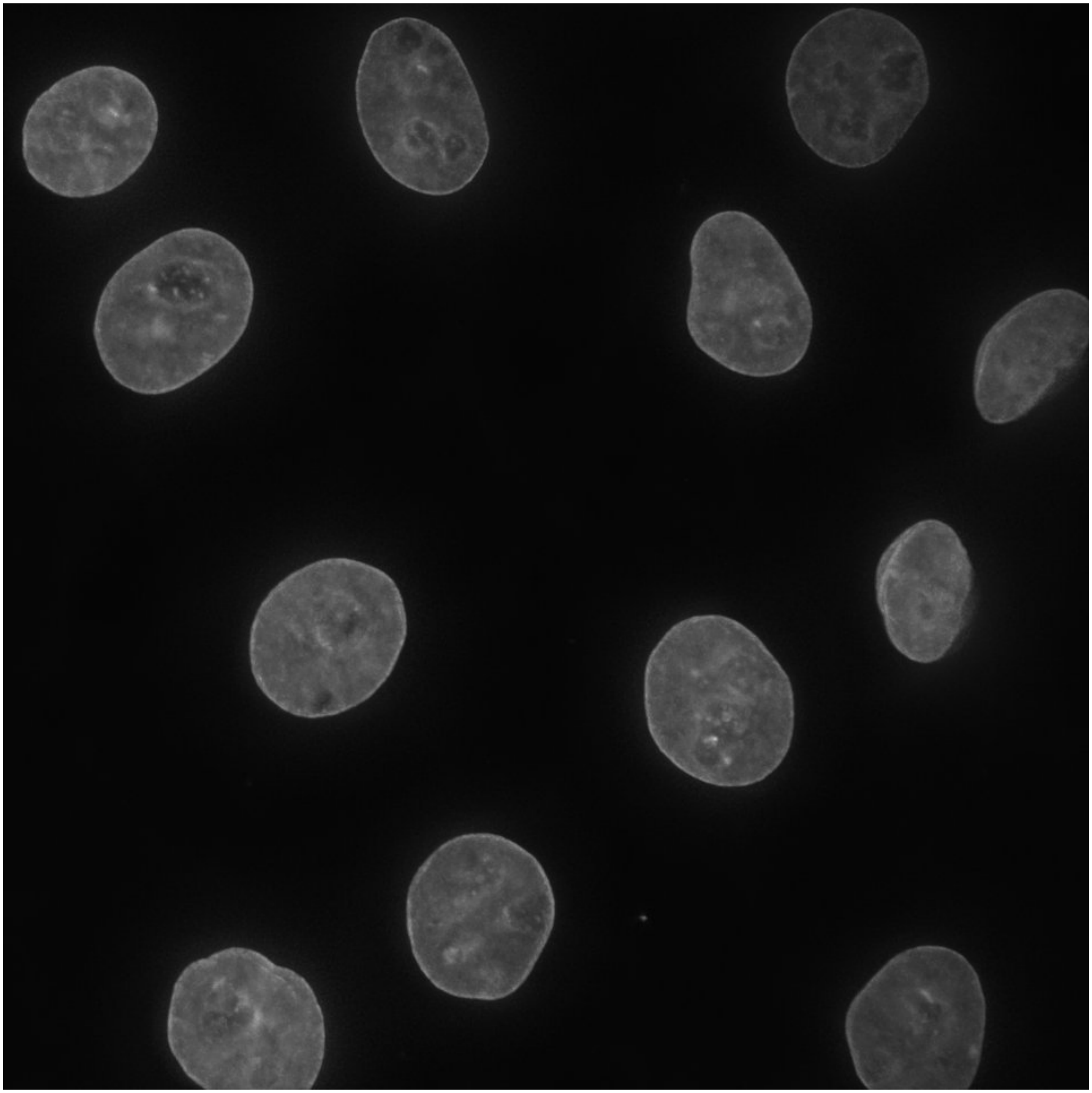

Fig4D_8_K102A_IAA_DOX_Ch2_CREST.jpg (1/1)

960 x 960

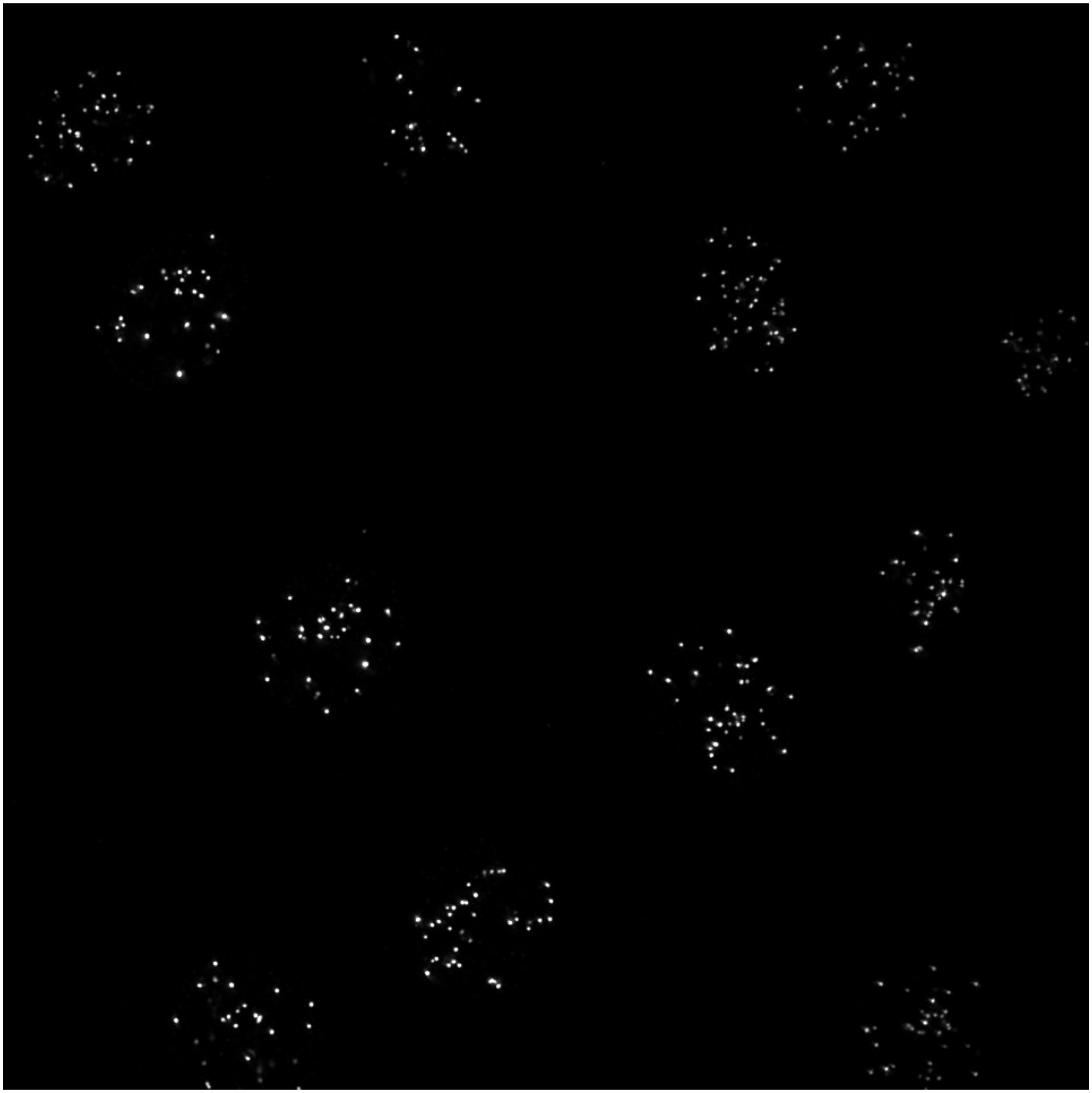

Fig4D_8_K102A_IAA_DOX_Ch3_GFP.jpg (1/1)

960 x 960

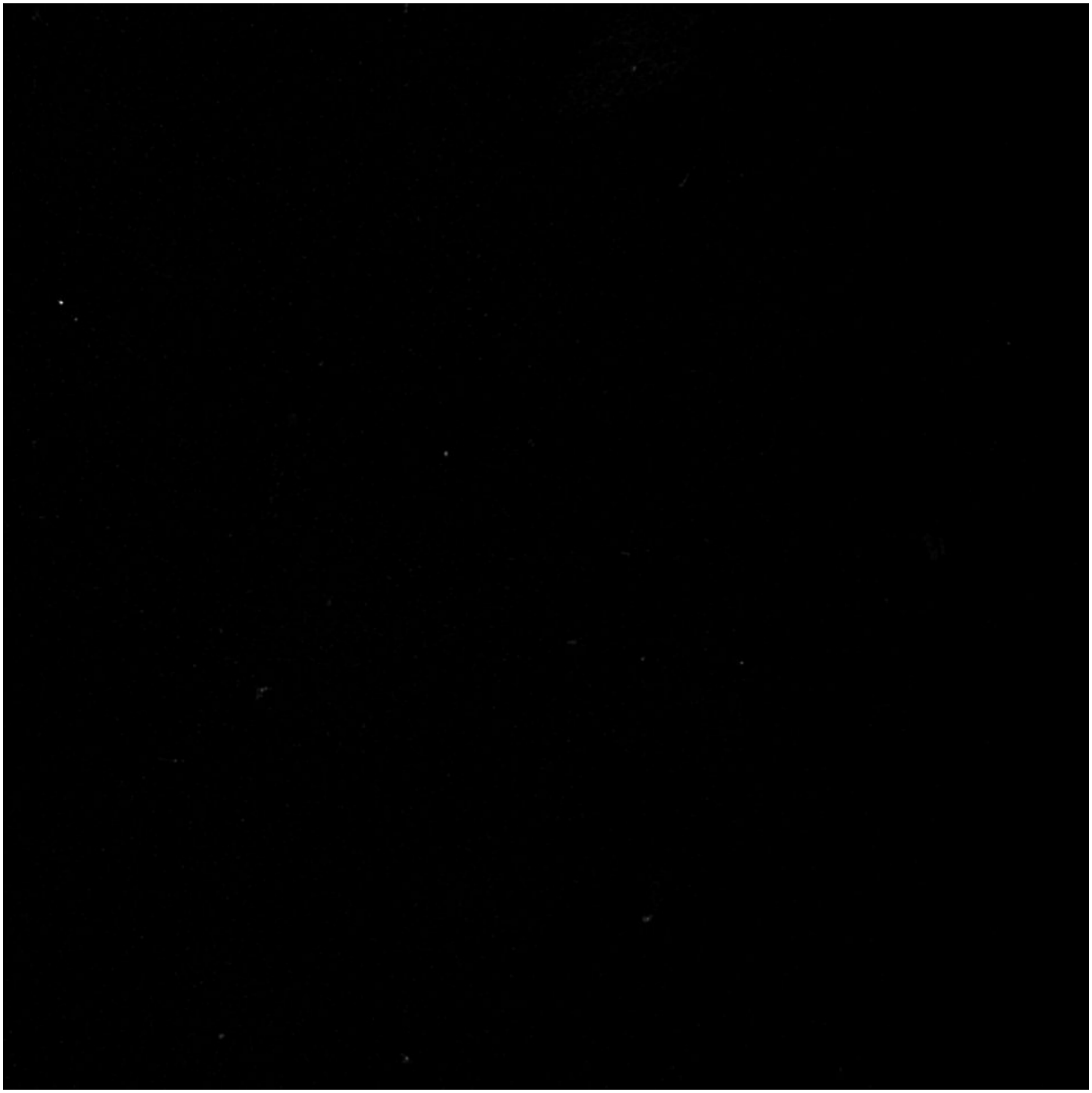

Fig4D_8_K102A_IAA_DOX_Ch4_mRuby.jpg (1/1)

960 x 960

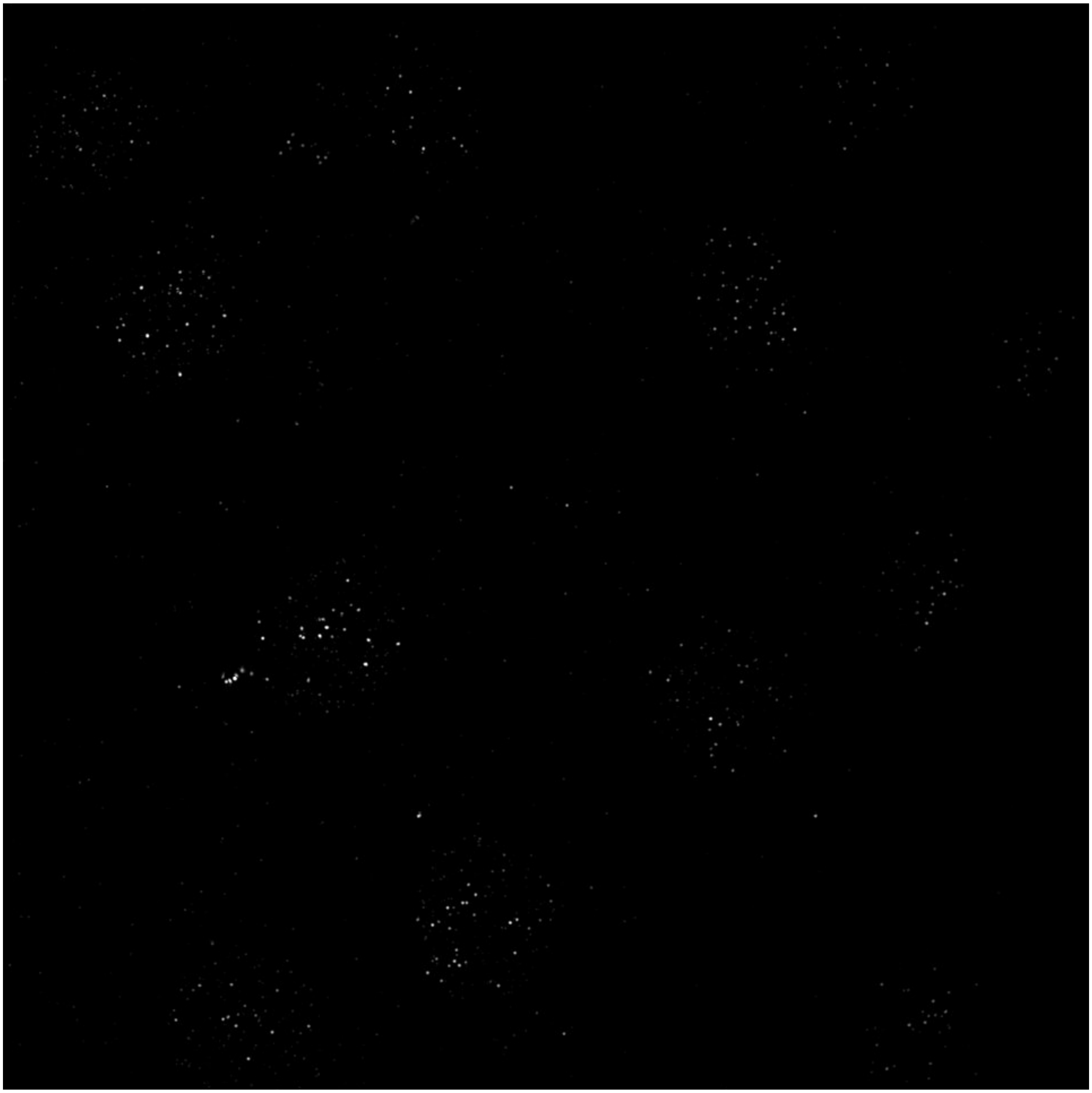

Supplement: Source Data Fig. 4 — Uncropped images for 4d [file 41594_2022_758_MOESM9_ESM.pdf]
