# Supplementary figures and images for: CENP-N promotes the compaction of centromeric chromatin
Source: Nat Struct Mol Biol. 2022 Apr 14;29(4):403–13. doi: 10.1038/s41594-022-00758-y (PMC9010303; doi:10.1038/s41594-022-00758-y)

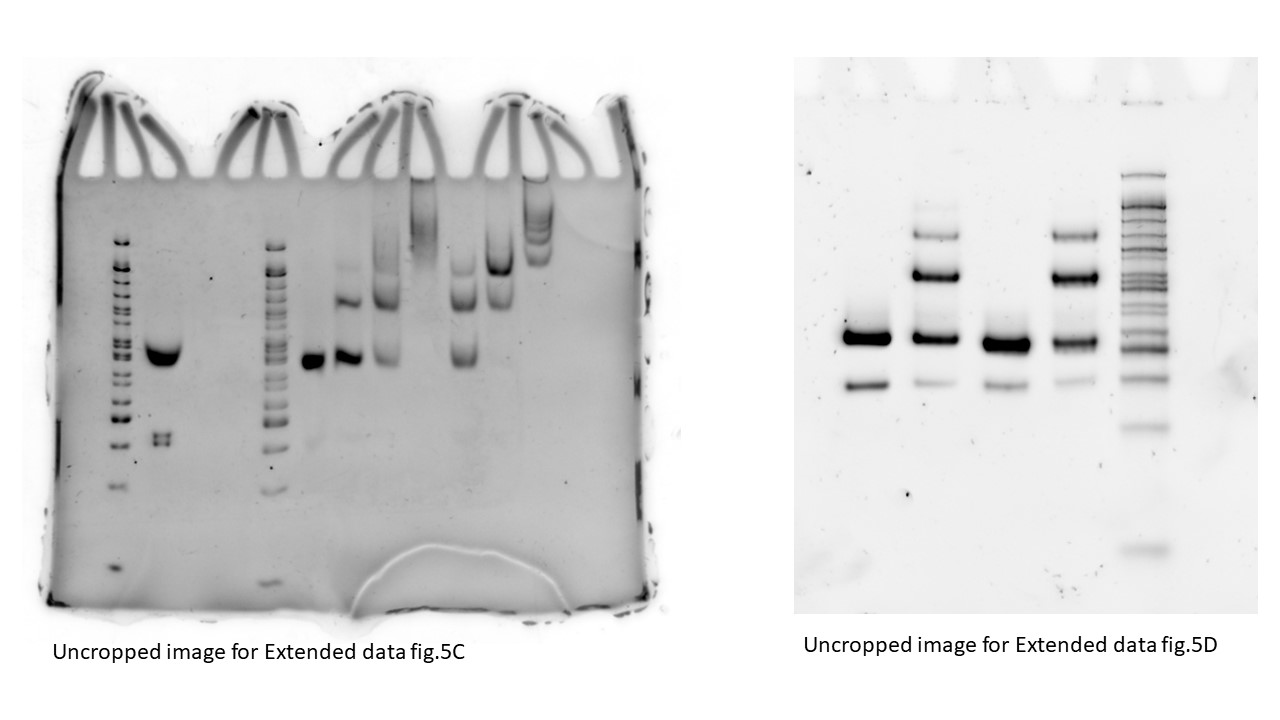

Supplement: Source Data Extended Data Fig. 5 — Uncropped images for Extended Data Fig. 5c,d [file 41594_2022_758_MOESM11_ESM.jpg]

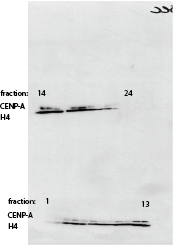

Supplement: Source Data Extended Data Fig. 9 — Uncropped images for Extended Data Fig. 9l [file 41594_2022_758_MOESM13_ESM.png]
